# Fine-tuning thiosemicarbazones with heterocyclic substituents: identification of styryl-dependent cysteine reactivity and potent anti-cancer activity

**DOI:** 10.1039/d6sc02195f

**Published:** 2026-07-15

**Authors:** Tharushi P. Wijesinghe, Mahendiran Dharmasivam, Busra Kaya, Paul V. Bernhardt, Masnun Naher, Des R. Richardson

**Affiliations:** a Centre for Cancer Cell Biology and Drug Discovery, Institute for Biomedicine and Glycomics, School of Environment and Science, Griffith University Parklands Drive Gold Coast Queensland 4222 Australia d.richardson@griffith.edu.au m.dharmasivam@griffith.edu.au; b School of Chemistry and Molecular Biosciences, University of Queensland Brisbane 4072 Australia; c Department of Pathology and Biological Responses, Nagoya University Graduate School of Medicine Nagoya 466-8550 Japan

## Abstract

The di-2-pyridylketone thiosemicarbazone analogue, DpC, progressed to clinical trials due to its favorable anti-cancer properties. However, trials were discontinued due to patient muscle pain linked to oxy-myoglobin oxidation. The more recent PPP4pT thiosemicarbazone and PPP4pSe selenosemicarbazone analogues successfully inhibited oxy-myoglobin oxidation but exhibited low solubility and reduced anti-proliferative activity. Herein, five new thiosemicarbazones bearing less-lipophilic heterocyclic substituents (morpholine, thiomorpholine, methyl-piperazine, pyrrolidine, and thiazolidine; the PPP4HAT analogues) demonstrated enhanced anti-cancer efficacy (IC_50_: 0.005–0.015 µM) and improved solubility. We report, for the first time, that l-cysteine selectively reacts with the α,β-unsaturated styryl moiety (CH=CH) of the thiosemicarbazones, forming Michael-type adducts. In contrast, the saturated non-styryl analogue (CH_2_–CH_2_) remained unreactive. Unexpectedly, Fe(iii) complexes of this series were less effective at suppressing oxy-myoglobin oxidation than the PPP4pT and PPP4pSe analogues, with docking simulations suggesting differences in predicted heme accessibility and ligand orientation. This study provides insights into important structure–activity relationships of thiosemicarbazones.

## Abstract 2

Novel PPP4HAT analogues with improved solubility and potency reveal structure–activity relationships and selective cysteine reactivity *via* Michael addition.

## Graphical abstract

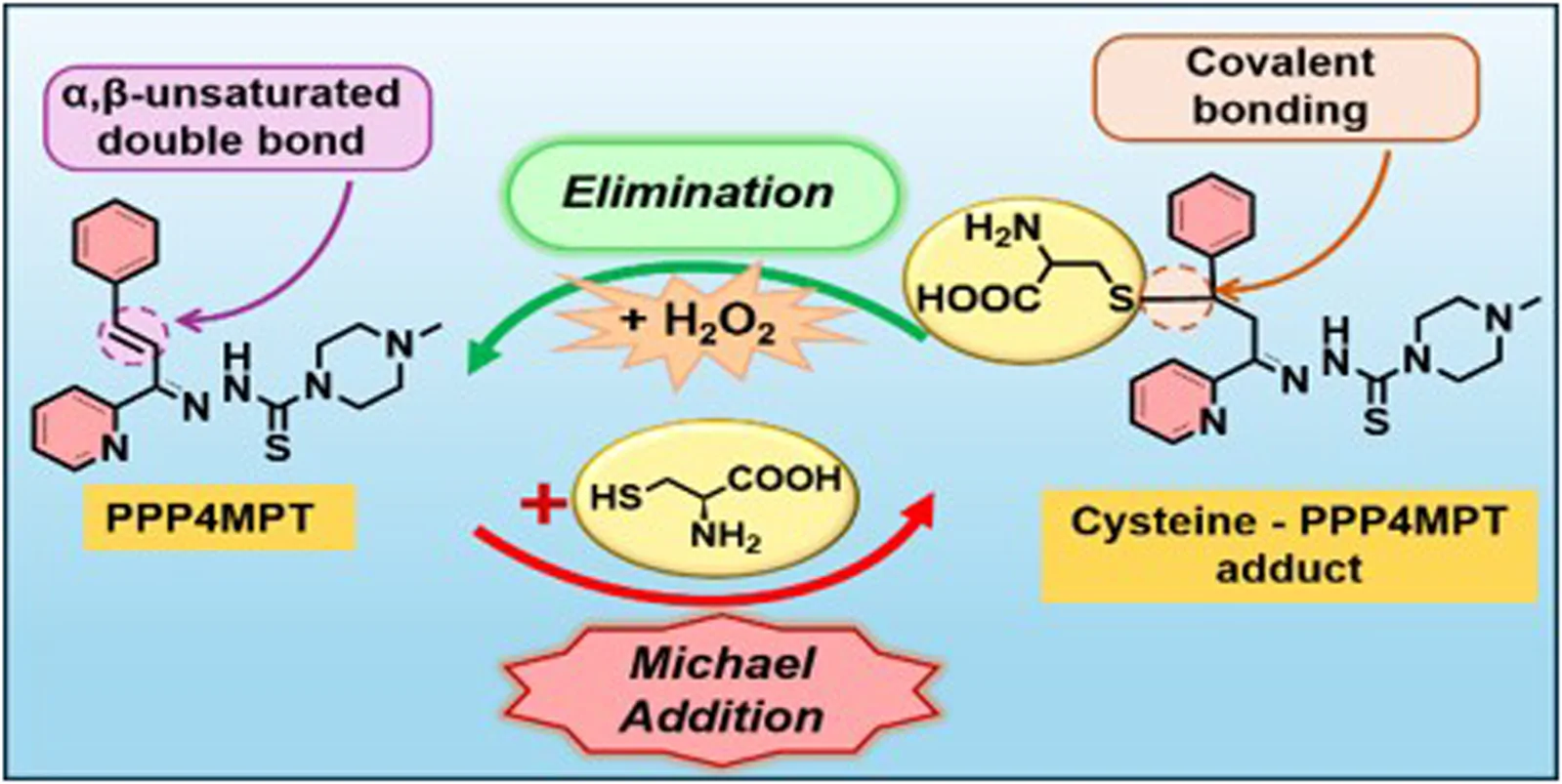

## Introduction

Lung cancer is the leading cause of cancer-related death globally, accounting for the highest incidence (12.4% of all cancers) and mortality (18.7% of all cancers) amongst both males and females.^1,2^ Lung cancer is categorized into two main subtypes: small-cell lung cancer (SCLC) and non-small-cell lung cancer (NSCLC).^3^ SCLC is the more aggressive type, leading to a poor prognosis.^4^ Among women, breast cancer is the leading cause of cancer death, accounting for ∼24% of cases.^1,5^ Breast cancer is classified according to the expression of estrogen receptor-α (ERα), progesterone receptor, and human epidermal growth factor receptor 2 (HER2),^6^ resulting in four major subtypes: (1) luminal A, positive for ERα and/or progesterone receptor, but negative for HER2; (2) luminal B, ERα and HER2 positive; (3) HER2-enriched type that is characterized by HER2 overexpression; and (4) triple-negative breast cancer (TNBC), which lacks expression of all three receptors,^7,8^ and is the most aggressive type.^9^

**Fig. 1:**
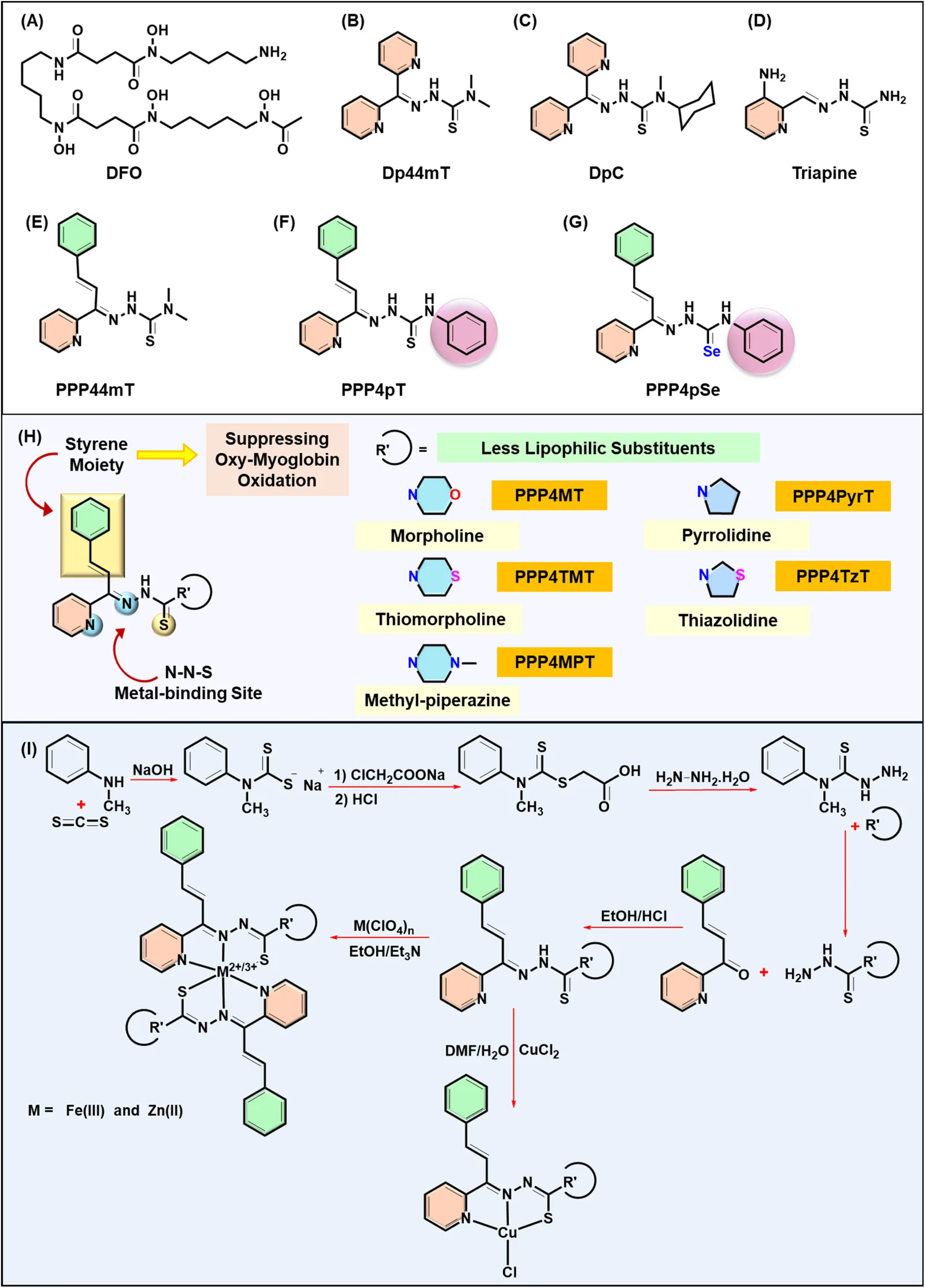
Line drawings of the structures of: (A) DFO; (B) Dp44mT; (C) DpC; (D) Triapine; (E) PPP44mT; (F) PPP4pT; (G) PPP4pSe; (H) key design features of the PPP4HAT heterocyclic-amine-substituted thiosemicarbazones; and (I) Synthetic route of the PPP4HAT series analogues and their Fe(iii), Cu(ii), and Zn(ii) complexes.

Considering the poor prognosis and limited treatment options for the later stages of these cancers, we sought to explore the efficacy of specifically designed metal-binding ligands against these malignancies, as it is a novel treatment approach for cancer.^10–14^ The sensitivity of tumors to this latter therapy is due to their high iron and copper requirements, which are crucial for cell cycle progression, proliferation, and metastasis.^15^ Due to tumor cell susceptibility to iron depletion, the classical iron chelator, desferrioxamine (DFO; Fig. 1A), has demonstrated some anti-proliferative activity against tumor cells, despite being developed for iron-overload treatment.^16,17^

Thiosemicarbazones are a ligand class that targets cancer cells and exhibits much greater anti-tumor activity relative to DFO.^11,18^ Particularly, the lead agents of the well-characterized di-2-pyridylketone thiosemicarbazone (DpT) series, namely di-2-pyridylketone-4,4-dimethyl-3-thiosemicarbazone (Dp44mT; Fig. 1B), and di-2-pyridylketone-4-cyclohexyl-4-methyl-3-thiosemicarbazone (DpC; Fig. 1C), have demonstrated marked anti-cancer activity against a variety of tumor types, including lung and breast cancer *in vitro* and *in vivo*.^14,19–27^

The selective anti-cancer activity of Dp44mT and DpC is mediated *via* the unique “double punch” mechanism.^23–25^ Regarding the “first punch,” these ligands bind intracellular iron (Fe) and copper (Cu), which are essential for cancer cell proliferation, inhibiting their growth.^23,25^ Subsequently, the iron and particularly copper-bound metal–ligand complexes undergo redox cycling through the Fenton reaction.^23,25^ This reaction constitutes the “second punch”, leading to the generation of cytotoxic reactive oxygen species (ROS) that induce lysosomal membrane permeabilization and cell death.^23,25^ Furthermore, due to their ability to bind intracellular iron pools, these ligands also up-regulate N-myc downstream regulated gene-1 (NDRG1), a potent metastasis suppressor,^28^ that acts on multiple signaling pathways.^29,30^ Notably, their lysosomal targeting properties and ability to act as *P*-glycoprotein (Pgp) substrates overcome Pgp-mediated resistance.^20,31,32^ Recently, this latter mechanism has been used by others to develop a different class of therapeutics.^33^ Hence, our DpT ligands target the “triad of death” in cancer, namely primary tumor growth, metastasis, and drug resistance *via* their multiple mechanisms of action.^34^

Due to the valuable properties of the DpT ligands, they have been studied extensively *in vitro* and *in vivo*, revealing that although the first-generation agent, Dp44mT, demonstrates potent anti-tumor efficacy, it caused cardiotoxicity in mice at higher doses.^19,24,35–37^ The second-generation lead agent, DpC, overcame this side effect while demonstrating superior anti-cancer activity, selectivity, efficient oral bioavailability, and optimal pharmacokinetics compared to Dp44mT.^14,38,39^ As a result, DpC progressed into phase I multicenter clinical trials (NCT02688101) in 2016.^18,34^ Unfortunately, the clinical trials were discontinued due to anecdotal muscle pain after DpC treatment.^18,40^

Experimental studies suggest this latter adverse effect may be attributed to the oxidation of oxy-myoglobin (oxy-Mb) to deleterious met-myoglobin (met-Mb) in muscle tissue, due to the high redox activity of thiosemicarbazone Fe complexes.^41^ In fact, the Fe(iii) complexes of the first and second generation compounds, Dp44mT and DpC, and other clinically trialed thiosemicarbazones, such as 3-aminopyridine-2-carboxaldehyde thiosemicarbazone (Triapine; Fig. 1D) and *N*′-(5,6-dihydroquinolin-8-yl)-4-(pyridin-2-yl)piperazine-1-carbothiohydrazide (COTI-2), have been demonstrated to induce oxy-Mb oxidation.^40–42^ This finding could explain the met-hemoglobinemia reported in patients treated with Triapine, with muscle pain also being reported in some patients.^43,44^

To prevent oxidation of oxy-Mb, a third generation of thiosemicarbazones, namely the 3-phenyl-1-(2-pyridinyl)-2-propen-1-one-thiosemicarbazone (PPPT) series, was designed with the addition of a styrene moiety.^40^ This substitution lowered the redox potential and caused steric blockade between the Fe(iii) complex and the heme plane of oxy-Mb and oxy-hemoglobin (oxy-Hb).^40^ Due to this modification, the Fe(iii) complex of the lead compound, (*E*)-3-phenyl-1-(2-pyridinyl)-2-propen-1-one-4,4-dimethyl-3-thiosemicarbazone (PPP44mT; Fig. 1E), led to decreased levels of oxy-Mb and oxy-Hb oxidation compared to the Fe(iii) complexes of Dp44mT and DpC.^40^

While PPP44mT reduced oxy-Mb oxidation levels, its inability to completely inhibit met-Mb formation led to the development of a fourth series of thiosemicarbazones, the phenyl-1-(2-pyridinyl)-2-propen-1-one-4-phenyl-3-thiosemicarbazone (PPP4pT; Fig. 1F) series.^42^ This series of ligands was developed by including an *N*^4^-phenyl group, while preserving the styrene group.^42^ Although the bulky and lipophilic styryl and phenyl groups of PPP4pT created a steric blockade that inhibited oxy-Mb oxidation, these same characteristics decreased solubility.^42^

The fifth generation of compounds was designed with greater structural alterations, where the thiocarbonyl moiety was replaced with selenocarbonyl, resulting in a *N*,*N*,*Se* coordination sphere.^45,46^ In addition, this (*E*)-3-phenyl-1-(2-pyridinyl)-2-propen-1-one-4-phenyl-3-selenosemicarbazone (PPP4pSe; Fig. 1G) series incorporated a modification to the styrene moiety with the addition of a methyl group.^45^ Multiple PPP4pSe series Fe(iii) complexes completely inhibited the oxidation of oxy-Mb to met-Mb, surpassing all previous generations of thiosemicarbazones from our laboratory.^45^ Although the poor solubility observed with PPP4pT was partly resolved in its selenosemicarbazone counterparts, namely the PPP4pSe series, the latter agents demonstrated reduced anti-proliferative activity compared to earlier thiosemicarbazone series.^45^

Herein, we describe the development of the sixth-generation thiosemicarbazones (Fig. 1H) incorporating five- or six-membered heterocyclic amine groups to prevent oxy-Mb oxidation and enhance their potent anti-cancer activity, while improving solubility. The novel (*E*)-3-phenyl-1-(2-pyridinyl)-2-propen-1-one-heterocyclic amine thiosemicarbazone (PPP4HAT) series preserves the styrene moiety from the third-generation PPPT series, while five different heterocyclic amines were introduced in place of the dimethylamino group (Fig. 1H).^40^ Ligands of this novel series, especially (*E*)-3-phenyl-1-(2-pyridinyl)-2-propen-1-one-methylpiperazine-1-thiosemicarbazone (PPP4MPT), demonstrated pronounced anti-proliferative activity and greater solubility than the previous PPP4pT series. We report that the α,β-unsaturated styryl moiety (CH=CH) of these thiosemicarbazones forms Michael-type adducts with physiologically abundant l-cysteine (l-Cys) but not glutathione (GSH), while structurally analogous saturated (CH_2_–CH_2_) ligands remained unreactive. Unexpectedly, the PPP4HAT series exhibited increased oxy-Mb oxidation relative to the PPP4pT and PPP4pSe analogues. This increased oxy-Mb oxidation was consistent with the docking simulations, which suggested differences in the predicted orientation and accessibility to the heme environment. This study delineates key structure–activity relationships in thiosemicarbazones.

## Results and discussion

### Chemical characterization

#### Synthesis of the PPP4HAT series and its Fe(iii), Cu(ii) and Zn(ii) complexes

The sixth-generation thiosemicarbazone analogues were synthesized *via* Schiff base condensation between the parent chalcone, 3-phenyl-1-(pyridin-2-yl)prop-2-en-1-one, and a series of heterocyclic amine-substituted thiosemicarbazides in the presence of HCl as a catalyst (Fig. 1I).^40,42,45^ Five new ligands were synthesized with different saturated nitrogen heterocycles, including morpholine, thiomorpholine, methyl-piperazine, pyrrolidine, and thiazolidine (Fig. 1H). Three metal complexes, Fe(iii), Cu(ii), and Zn(ii), were also prepared and characterized for each ligand using standard methods (Fig. 1I).^40,42,47^

#### X-ray crystallography

**Fig. 2:**
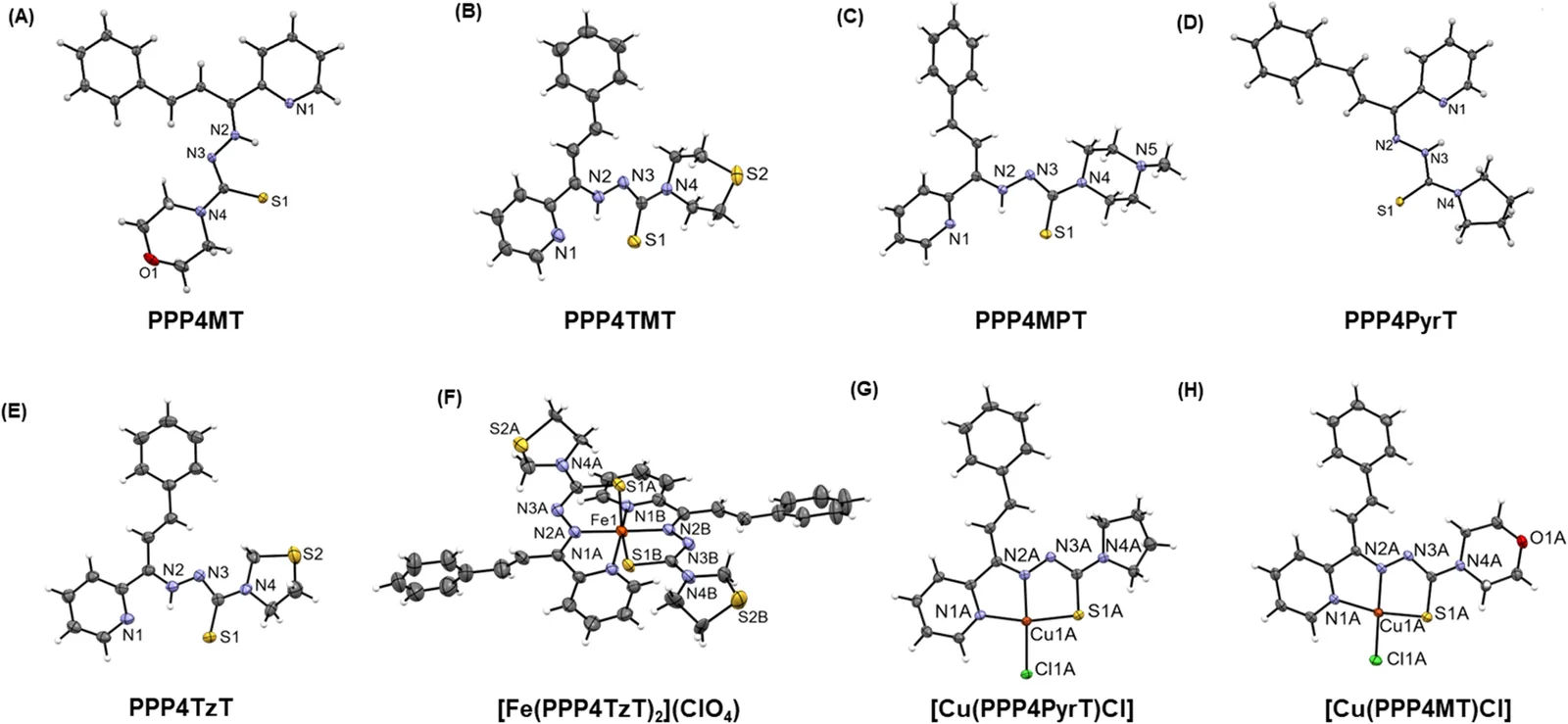
Crystal structures of: (A) PPP4MT, (B) PPP4TMT, (C) PPP4MPT, (D) PPP4PyrT, (E) PPP4TzT, and (F) [Fe(PPP4TzT)_2_](ClO_4_), selected bond lengths (Å): Fe1–N1A 1.980(4); Fe1–N2A 1.915(4); Fe1–SA 2.231(1). The ClO_4_^−^ anion is not shown for clarity. (G) [Cu(PPP4PyrT)Cl], selected bond lengths (Å): Cu1A–N1A 2.004(1); Cu1A–N2A 1.967(1); Cu1A–S1A 2.2424(5); Cu1A–Cl1A 2.2444(5), and (H) [Cu(PPP4MT)Cl] (30–50% probability ellipsoids).

Single-crystal X-ray diffraction was employed to elucidate the structures of the five heterocyclic amine-substituted thiosemicarbazone ligands (Fig. 2A–E), one Fe(iii) complex (Fig. 2F), and two Cu(ii) complexes (Fig. 2G and H). The crystal and refinement data are provided in Table S1. Unit cell packing diagrams are demonstrated for the five ligands (Fig. S1A–E), and the three complexes, [Fe(PPP4TzT)_2_]^+^ (Fig. S2A), [Cu(PPP4PyrT)Cl] (Fig. S2B), and [Cu(PPP4MT)Cl] (Fig. S2C). The structural analyses offer insight into their biological activity.

The structures of five of the thiosemicarbazone ligands are shown in Fig. 2A–E. Despite their common parent structure, tautomeric and conformational isomerism is present. Four isomeric forms can be considered (Fig. S3A–D). When N_2_ is protonated, a zwitterionic tautomer is observed with a negative charge residing on S1 and a positive charge on N2 (Fig. S3A and B). This finds N1, N2, and S1 in adjacent positions stabilized by H-bonding, giving the *E* isomeric form (of the C=N double bond). The styryl group can adopt two rotational isomers, where the pyridyl and phenyl rings are *anti* (Fig. S3A) or *syn* (Fig. S3B). The *Z*-isomer (Fig. S3C and D) adopts an entirely different structure, demonstrating that N3 is protonated and H-bonded to the pyridyl N-atom.

The structures of the ligands, PPP4MT (Fig. 2A), PPP4TMT (Fig. 2B), and PPP4TzT (Fig. 2E), adopt a common isomeric form (Fig. S3A, *E*, *anti*). In contrast, PPP4MPT (Fig. 2C) appears in the *E*, *syn* form (Fig. S3B). When the *syn* conformation of the styryl group is present, the ligand is distinctly non-planar due to H⋯H repulsion between the 3-pyridyl and nearby styrene H-atoms. The structure of PPP4PyrT (Fig. 2D) is unique in this series, with N3 being protonated, and the *Z* isomeric form present (Fig. S3C). Again, the *syn* conformation of the styryl group results in an out-of-plane distortion to avoid the clash between the pyridyl ring and the styryl group.

Examining related thiosemicarbazones,^48,49^ we previously observed that the *E* isomer (and zwitterionic tautomer) is generally favored when N4 has no H atoms attached, so the structure of PPP4PyrT is notable in opposing this trend (Fig. 2D). A small but significant elongation of the C–S1 bond is observed for the zwitterionic tautomer's, PPP4MT (1.697(2) Å), PPP4TMT (1.703(2) Å) and PPP4MPT (1.716(1) Å) compared with PPP4PyrT (1.681(2) Å) (*cf*. Fig. 2A, B, and D). This indicates an increased contribution of the ene-thiolate resonance form within the thiosemicarbazone framework. Disorder of the thiazolidine ring in PPP4TzT (not shown in Fig. 2E) reduced the precision of the bond lengths, so this structure is not included in this comparison. In solution, the *E* and *Z* isomers coexist in a slow equilibrium and give rise to complicated ^1^H NMR spectra exhibiting multiple NH resonances in the downfield region (12–15 ppm) with non-integral ratios (Fig. S13, S15, S17, S19, and S21). These signals arise from *E*/*Z* isomerism typical of thiosemicarbazones, where the different isomers interconvert slowly on the NMR timescale. Similarly, the ^13^C NMR spectra of the ligands (Fig. S14, S16, S18, S20, and S22) exhibit more resonances than there are C-atoms in each compound due to this same isomerization.

Three complexes from the PPP4HAT series were structurally characterized, namely [Fe(PPP4TzT)_2_](ClO_4_), [Cu(PPP4PyrT)Cl], and [Cu(PPP4MT)Cl], which illustrate the two main types of complexes that form with tridentate *N*,*N*,*S* ligands from this family (Fig. 2F–H). The Fe(iii) complex exhibits a distorted octahedral geometry, with Fe–N and Fe–S bond lengths consistent with a low-spin d^5^ ground state (Fig. 2F).^40,42^ As found in the structure of the free ligand, the thiazolidine ring of each ligand is disordered (not shown in Fig. 2F).

The Cu(ii) complexes, [Cu(PPP4PyrT)Cl] and [Cu(PPP4MT)Cl], both crystallize as neutral 1 : 1 Cu : ligand complexes, with each metal center coordinated by one deprotonated thiosemicarbazone ligand and one chlorido ligand (Fig. 2G and H). These structures are consistent with the 1 : 1 complexes typical of biologically active Cu(ii)-thiosemicarbazone systems.^14,40,47,50^ Both structures comprise two complexes in the asymmetric unit, and [Cu(PPP4MT)Cl] forms morpholine-bridged dimers with a weak Cu–O axial bond (Cu1B–O1A 2.564(1) Å) connecting the two monomeric units (Fig. S2C). This latter dimer probably dissociates in solution. Based on the 
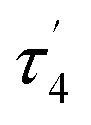
 parameter of Okuniewski *et al.*,^51^ where 
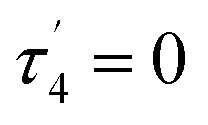
 corresponds to ideal square-planar geometry, and 
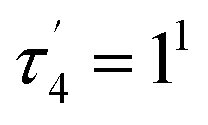
 corresponds to ideal tetrahedral geometry, both complexes are best described as distorted square planar, with 
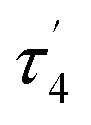
 values of 0.16 for [Cu(PPP4PyrT)Cl], and 0.19 for [Cu(PPP4MT)Cl].††
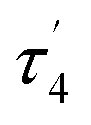
 = (*β* − *α*)/219 + (180 − *β*)/141, where *α* and *β* are the two largest coordinate angles.

Each molecule adopts an *E*, *anti*-conformation across the imine and thiosemicarbazone backbone, with the coordination sphere around Cu(ii) formed by the pyridine N, the hydrazinic N, the thione S, and a chlorido ligand (Fig. 2G and H). The coordinated ligand is present in its monoanionic form following deprotonation of the hydrazinic nitrogen (N2). The resulting negative charge is delocalized across the N–N–C=S thiosemicarbazone backbone, with a significant contribution from the thiolate resonance form, which stabilizes coordination to the Cu(ii) center. For [Cu(PPP4MT)Cl], the key bond lengths are Cu–N (pyridine) = 1.967(1) Å and 1.968(1) Å; Cu–N (hydrazinic) = 2.004(1) Å and 1.997(1) Å; Cu–S (thione) = 2.2424(5) Å and 2.2274(5) Å; Cu–Cl = 2.2444(5) Å and 2.2339(5) Å (Fig. 2H). These are comparable to the bond metrics observed in [Cu(PPP4PyrT)Cl] (Fig. 2G), indicating that substitution at the terminal amine does not significantly perturb the approximate square planar Cu(ii) coordination environment.

#### Electrochemical modulation and fine-tuning of Fe(iii) and Cu(ii) complexes of the PPP4HAT analogues *via* terminal amine substitution

The redox properties of Fe(iii) thiosemicarbazone complexes (higher positive potentials) are linked to their ability to drive biologically relevant, detrimental, oxidative processes, including the oxidation of oxy-Mb and oxy-Hb.^40,42^ In contrast, lower negative redox potentials of thiosemicarbazone Cu(ii) complexes correlate with their anti-proliferative activity.^40,42,45,50^

A detailed explanation of the electrochemistry of the Fe(iii) and Cu(ii) complexes is provided in the SI as Supporting Results and discussion, which refers to Table S2 and Fig. S4. In summary, a ligand structure/redox potential relationship was observed across both Fe(iii) and Cu(ii) complexes. For the Fe(iii) complexes, increasing electron donation and decreasing steric bulk at the terminal amine lowers the Fe^III/II^ potential, stabilizing the ferric state^40,52^ and mitigating the off-target oxidation of oxy-Mb.^40,42,45,46^ Examining the Cu(ii) complexes, stronger electron-donating heterocyclic amines shift the Cu^II/I^ couple anodically (*i.e.*, less negative). In contrast, weaker or more hindered donors shift it cathodically (*i.e.*, more negative).

The redox potentials of both Fe(iii) and Cu(ii) PPP4HAT complexes lie within the biologically relevant range of −0.4 to +0.8 V *versus* the normal hydrogen electrode (NHE), underscoring their suitability for modulating physiological redox processes.^53^ These fine-tuned analogues highlight how targeted ligand modifications can precisely tune metal redox potentials. This series of compounds provides a platform for tuning metal redox properties that may help minimize undesirable oxidative processes (*i.e.*, Fe(iii) complexes with low positive potentials) and promote anti-proliferative activity (*i.e*., Cu(ii) complexes with low negative potentials). These electrochemical trends are discussed further in relation to the anti-proliferative activity of the ligands and their metal complexes and the oxy-Mb oxidation studies presented later.

### Biological studies

#### Anti-proliferative activity of the PPP4HAT ligands in breast cancer cells

Herein, the anti-proliferative activity of the novel PPP4HAT series is examined after 24- and 72 h incubations in two breast cancer cell models, MDA-MB-231 (TNBC; aggressive phenotype) and MCF-7 (luminal A subtype; Table 1).^7,8^ In these studies, the activity of the PPP4HAT series was evaluated relative to the control compounds, DFO,^17,54^ Triapine,^55,56^ and the lead analogues of previous thiosemicarbazone series, namely Dp44mT, DpC, PPP44mT, and PPP4pT.^19,24,25,40,42^

**Table 1:** Inhibition of the proliferation (IC_50_; µM) of MDA-MB-231 and MCF-7 breast cancer cells by DFO, Triapine, Dp44mT, DpC, PPP44mT, and PPP4pT relative to the PPP4HAT series of ligands and their Fe(iii), Cu(ii), and Zn(ii) complexes. Proliferation was examined using the MTT assay after a 24- or 72 h incubation at 37 °C. Results are Mean ± SD (3 experiments)

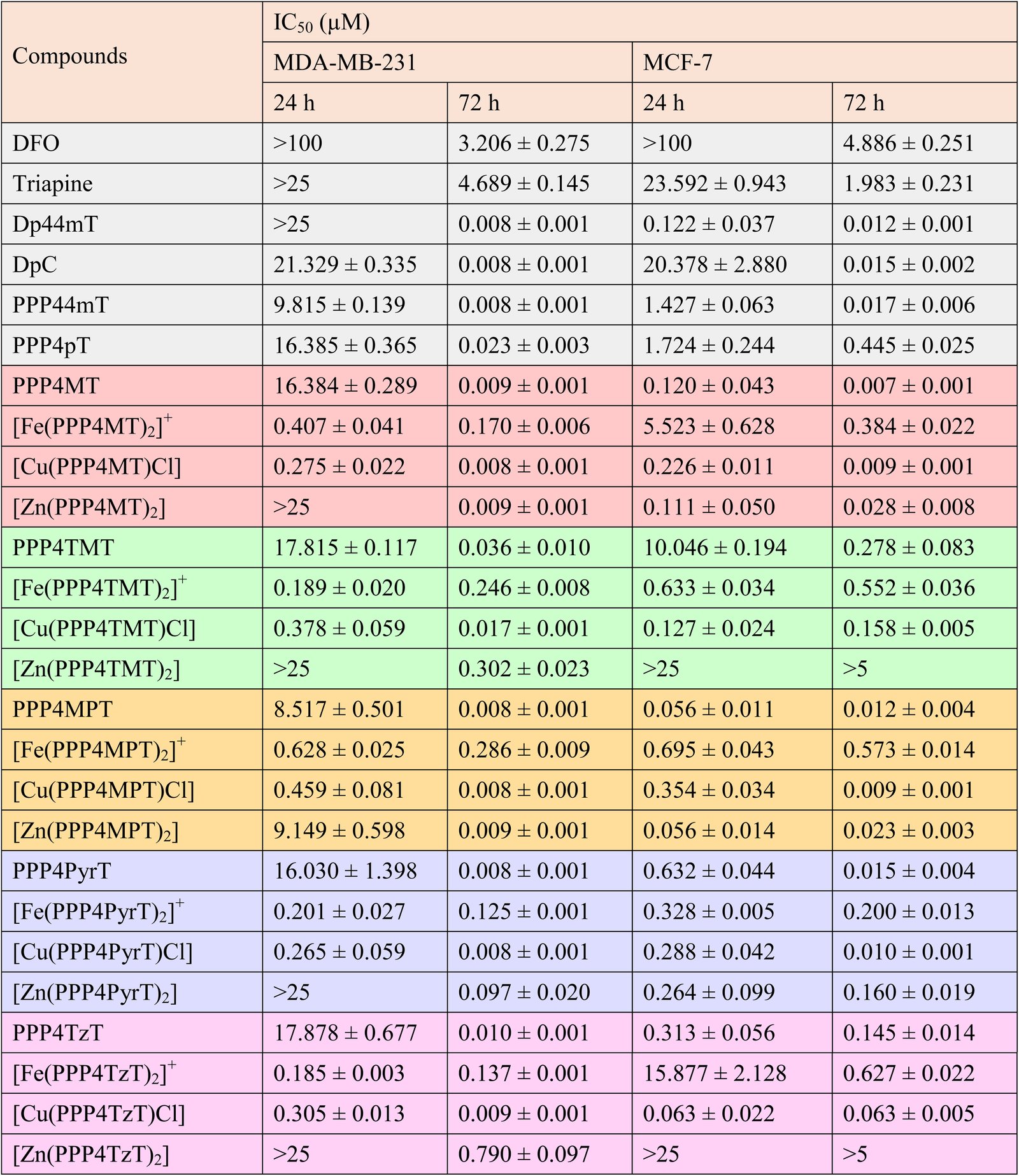

After a 24 h incubation, among the positive control ligands, DFO showed minimal activity in both cell models (IC_50_: >100 µM). Of the control thiosemicarbazones, Triapine demonstrated the poorest efficacy overall in MDA-MB-231 cells (IC_50_: >25 µM) and MCF-7 cells (IC_50_: 23.592 µM), with DpC exhibiting only slightly greater activity after 24 h (IC_50_: 21.329 µM and 20.378 µM, respectively; Table 1). PPP44mT demonstrated the highest potency among the controls in MDA-MB-231 cells (IC_50_: 9.815 µM), whereas Dp44mT remained the most active control ligand in MCF-7 cells (IC_50_: 0.122 µM) but showed little activity in MDA-MB-231 cells (>25 µM; Table 1). These findings align with the increased drug resistance observed in the TNBC phenotype compared to luminal A cells.^9,57^

Upon a 24 h incubation with the PPP4HAT series, the methyl-piperazine analogue, PPP4MPT, displayed the most potent activity in MDA-MB-231 (IC_50_: 8.517 µM) and MCF-7 cells (IC_50_: 0.056 µM; Table 1). The remaining PPP4HAT ligands showed lower efficacy, with IC_50_ values ranging from 16.030–17.878 µM in MDA-MB-231 and 0.120–10.046 µM in MCF-7 cells (Table 1). Similar to the control compounds, the more aggressive TNBC MDA-MB-231 cells demonstrated markedly greater resistance to PPP4HAT ligands than the luminal A MCF-7 subtype. Collectively, after 24 h, PPP4MPT was significantly (*p* < 0.001–0.01) more effective than clinically trialed DpC in both breast cancer cell types (Table 1).

#### Anti-proliferative activity of Fe(iii), Cu(ii), and Zn(ii) complexes in breast cancer cell types after 24 h

Coordination to Fe(iii), Cu(ii), or Zn(ii) is known to modulate the pharmacological activity of thiosemicarbazones.^40,42,45,46^ Therefore, the activity of the PPP4HAT complexes was also assessed in breast cancer cell types (Table 1). Across almost all ligands in both cell types, the 1 : 1 Cu(ii) complexes consistently demonstrated similar or significantly (*p* < 0.001–0.05) greater activity than their parent ligands after 24 h. The only exceptions were [Cu(PPP4MT)Cl] and [Cu(PPP4MPT)Cl], which demonstrated significantly (*p* < 0.01) lower anti-proliferative activity than their parent ligands, but only in MCF-7 cells after 24 h (Table 1). This general trend of activity aligns with previous thiosemicarbazone series, where the Cu(ii) complexes elicit enhanced cytotoxicity.^40,42,45,46^

After a 24 h incubation, all Fe(iii) complexes also displayed markedly and significantly (*p* < 0.001) greater activity relative to their ligands against MDA-MB-231 cells (Table 1). However, in MCF-7 cells, [Fe(PPP4MT)_2_]^+^, [Fe(PPP4MPT)_2_]^+^, and [Fe(PPP4TzT)_2_]^+^, demonstrated significantly (*p* < 0.001–0.01) reduced efficacy relative to their respective ligands. In contrast, [Fe(PPP4PyrT)_2_]^+^ and [Fe(PPP4TMT)_2_]^+^ showed significantly (*p* < 0.001) greater efficacy. After a 24 h incubation, the Zn(ii) complexes generally exhibited the lowest activity across both breast cancer cell models (Table 1). Exceptions included [Zn(PPP4MPT)_2_], which demonstrated comparable activity to its ligand in both MDA-MB-231 and MCF-7 cells after 24 h, and [Zn(PPP4MT)_2_] and [Zn(PPP4PyrT)_2_], which showed equal or greater activity than the ligand in MCF-7 cells, respectively (Table 1).

#### After 72 h, PPP4MT, PPP4MPT, and PPP4PyrT exhibited the greatest anti-proliferative activity in breast cancer cells

Over 72 h, the PPP4HAT ligands demonstrated a marked increase in potency across both breast cancer models (Table 1), consistent with earlier thiosemicarbazone generations.^40,42,45,46,58^ PPP4MT, PPP4MPT, and PPP4PyrT emerged as the most active ligands across both cell types (IC_50_: 0.007–0.015 µM), achieving activity comparable to the highly potent third-generation analogue, PPP44mT. The thiomorpholine analogue, PPP4TMT, exhibited the lowest activity among the PPP4HAT ligands after 72 h (Table 1). Notably, the resistance of the MDA-MB-231 cell type during short incubations was not observed after a 72 h incubation, indicating time-dependent anti-cancer efficacy.

#### 1 : 1 Cu(ii) Complexes of PPP4MT, PPP4MPT, and PPP4PyrT were the most effective after 72 h

Across the breast cancer models, all Cu(ii) complexes demonstrated very similar or significantly (*p* < 0.001–0.05) improved activity relative to their ligands at 72 h. The Cu(ii) complexes of PPP4MT, PPP4MPT, and PPP4PyrT, were the most potent overall (Table 1). In contrast, the Zn(ii) complexes showed a slower onset of anti-proliferative activity with [Zn(PPP4MT)_2_] and [Zn(PPP4MPT)_2_] becoming similar in efficacy relative to their Cu(ii) complexes after 72 h in MDA-MB-231 and MCF-7 cells. This latter observation suggested time-dependent transmetalation may be occurring from the Zn(ii) to the Cu(ii) complex, as observed for related thiosemicarbazones.^40,46^ The Fe(iii) complexes exhibited significantly (*p* < 0.001) less anti-proliferative activity relative to the ligands after a 72 h incubation across both cell types (Table 1).

Similar activity patterns were observed in the DMS-53 (SCLC) and H1299 (NSCLC) lung cancer cell models, with the full IC_50_ datasets provided in the SI (Table S3). Collectively, these data demonstrate that PPP4MPT is the most potent ligand of the PPP4HAT series across breast cancer and lung cancer cell models after 24- and 72 h incubations (Table 1 and S3). Multiple PPP4HAT ligands demonstrated improved anti-proliferative activity relative to the fourth-generation PPP4pT analogue and earlier thiosemicarbazone series. Notably, the 1 : 1 Cu(ii) complexes consistently showed markedly higher activity than their corresponding Fe(iii) and Zn(ii) complexes (Table 1 and S3), underscoring the central role of copper coordination in enhancing cytotoxic potency.

#### Physicochemical properties and potential bioavailability of the PPP4HAT series

Considering the anti-proliferative properties of these newly developed thiosemicarbazones, it was essential to assess their structure–activity relationships, physicochemical properties, and bioavailability as potential clinical agents (Table S4).^59–61^ All five members of the PPP4HAT series complied with Lipinski's Rule of 5 with no violations (Table S4). This is a significant improvement over the two previous thiosemicarbazone generations, the PPP4pT and PPP4pSe series, where multiple analogues had calculated partition coefficient (log *P*_calc_) exceeding 5,^42,45^ violating one of Lipinski's rules.^42,45,59^ The log *P*_calc_ is an important parameter that reflects a molecule's lipophilicity, where a lower log *P* value suggests greater solubility in aqueous solutions and permeability, leading to better bioavailability.^62,63^ The lower log *P*_calc_ values of the PPP4HAT analogues (3.07–3.62) point toward improved solubility relative to the log *P*_calc_ of the earlier-generation analogue, PPP4pT (4.04; Table S4). In this study, these analogues exhibited appreciable solubility in aqueous solvent systems.

The physicochemical properties of the metal complexes were also evaluated (Table S4). The Fe(iii) and Zn(ii) complexes of the PPP4HAT violated multiple Lipinski's rules due to their higher molecular weights (>500 Daltons) and log *P* values (>5). In contrast, the 1 : 1 ligand: Cu(ii) complexes complied. These properties may account, in part, for the greater anti-proliferative activity of the 1 : 1 ligand: Cu(ii) complexes across all cell types after 24- and 72 h incubations (Table 1 and S3).

#### Reactivity of styryl-containing ligands *versus* the non-styryl analogue with l-cysteine

Endogenous intracellular reductants such as l-Cys and GSH are key nucleophiles that regulate metal coordination and redox balance in lysosomal and cytosolic compartments.^45,64,65^ Previous studies showed that PPP4pT and PPP4pSe Cu(ii) complexes undergo thiol-dependent coordination and speciation changes in the presence of l-Cys, GSH, and l-ascorbate.^45^ However, the intrinsic thiol reactivity of the free ligands themselves has never been examined. This knowledge gap is essential to investigate because l-Cys and GSH are present at high intracellular concentrations,^66,67^ and potentially may directly modify stability, cellular behavior before metal binding occurs, or ligand structure (*e.g*., *via* adduct formation upon attack of the unsaturated styryl CH=CH bond).

Therefore, determining whether these ligands directly participate in thiol addition chemistry under physiological conditions is essential for understanding their biological activity. Given the well-established Michael reactivity of α,β-unsaturated double bonds toward nucleophiles,^68^ we hypothesized that the styryl CH=CH bond in these thiosemicarbazones could act as a Michael acceptor for physiologically abundant thiols, such as l-Cys and GSH.^67–69^

**Fig. 3:**
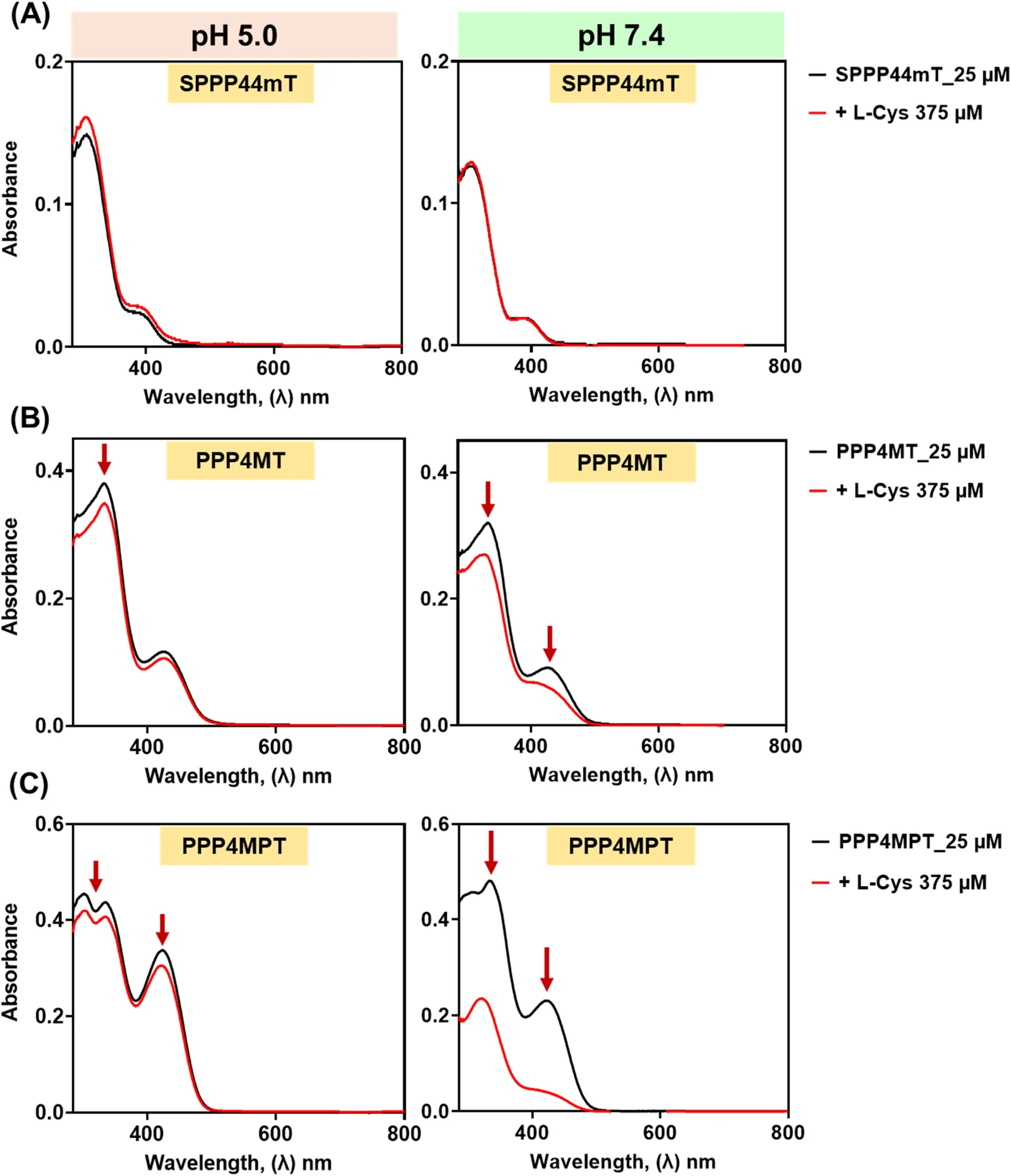
UV-vis spectra of: (A) SPPP44mT (25 µM), (B) PPP4MT (25 µM), and (C) PPP4MPT (25 µM) and l-Cys (375 µM) at lysosomal pH 5.0 and cytosolic pH 7.4 before (black curves) and after (red curves) a 6 h/20 °C incubation.

To initially test this, UV-vis spectrometry was performed under lysosomal (pH 5.0, acetate buffer) and cytosolic (pH 7.4, HEPES) conditions in a physiologically relevant 0.14 M NaCl solution.^40,45^ Ligands were prepared as 10 mM stock solutions in DMSO and diluted to 25 µM in the respective buffers (final [DMSO] = 0.25% v/v). The styryl-containing analogues, PPP4MT and PPP4MPT, were compared with the non-styryl, saturated (CH_2_–CH_2_) analogue, 3-phenyl-1-(2-pyridinyl)propan-1-one 4,4-dimethyl-3-thiosemicarbazone (SPPP44mT)^40^ (Fig. 3A–C). Each ligand (25 µM) was treated with a 15-fold excess of l-Cys (375 µM), and the spectra were recorded after 6 h/20 °C, as preliminary studies indicated equilibrium had been established after this time period.

**Fig. 4:**
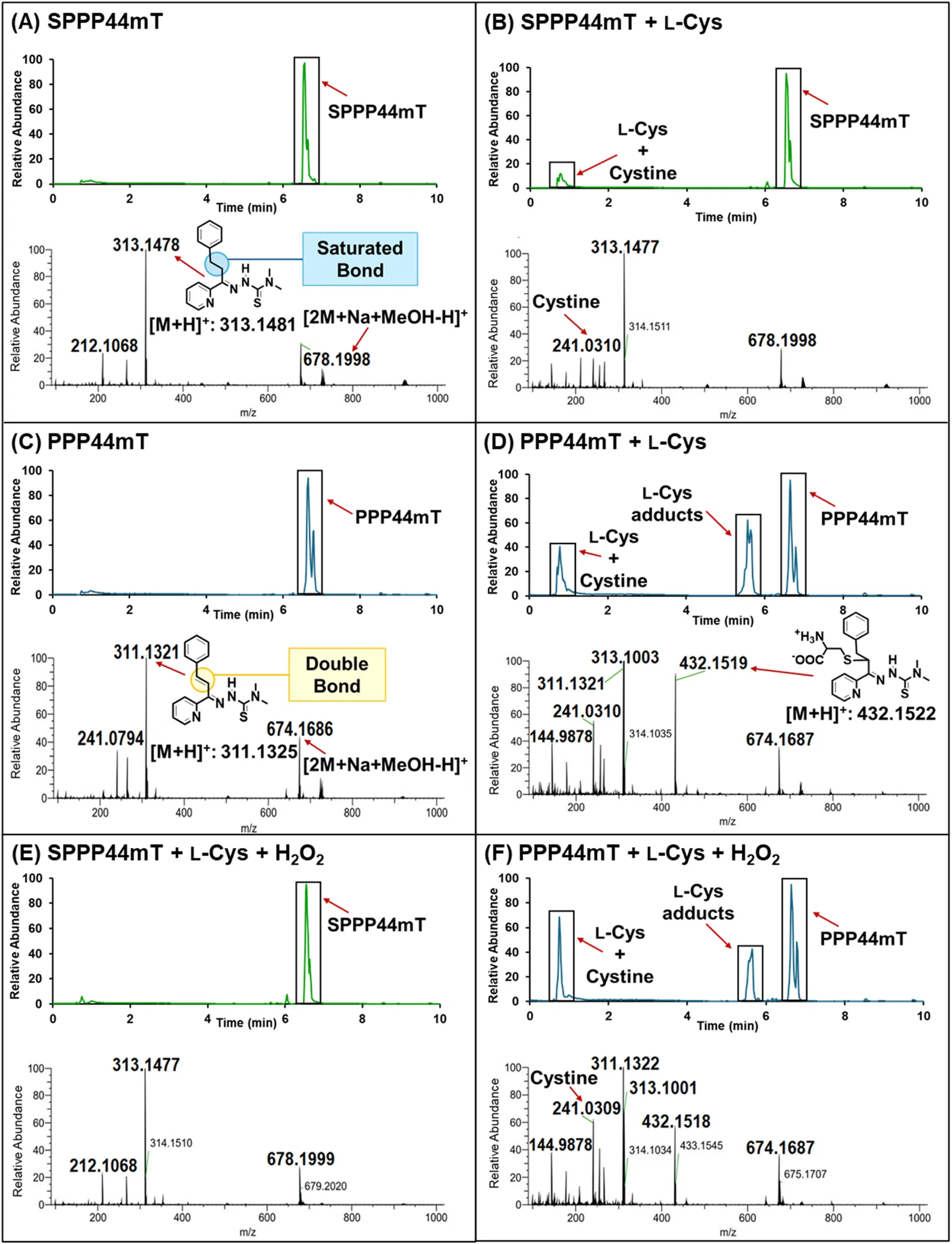
LC-MS analysis of: (A) SPPP44mT (500 µM), (B) SPPP44mT (500 µM) + l-Cys (5 mM), (C) PPP44mT (500 µM) and (D) PPP44mT (500 µM) + l-Cys (5 mM) after a 6 h/20 °C incubation in DMSO/H_2_O (7 : 3 v/v). Following this latter incubation, H_2_O_2_ (10 mM) was added to the respective solutions (E and F), and the incubation continued for 2 h/20 °C. After all incubations, the solutions were diluted 1 : 1 with MeOH prior to LC-MS analysis.

**Fig. 5:**
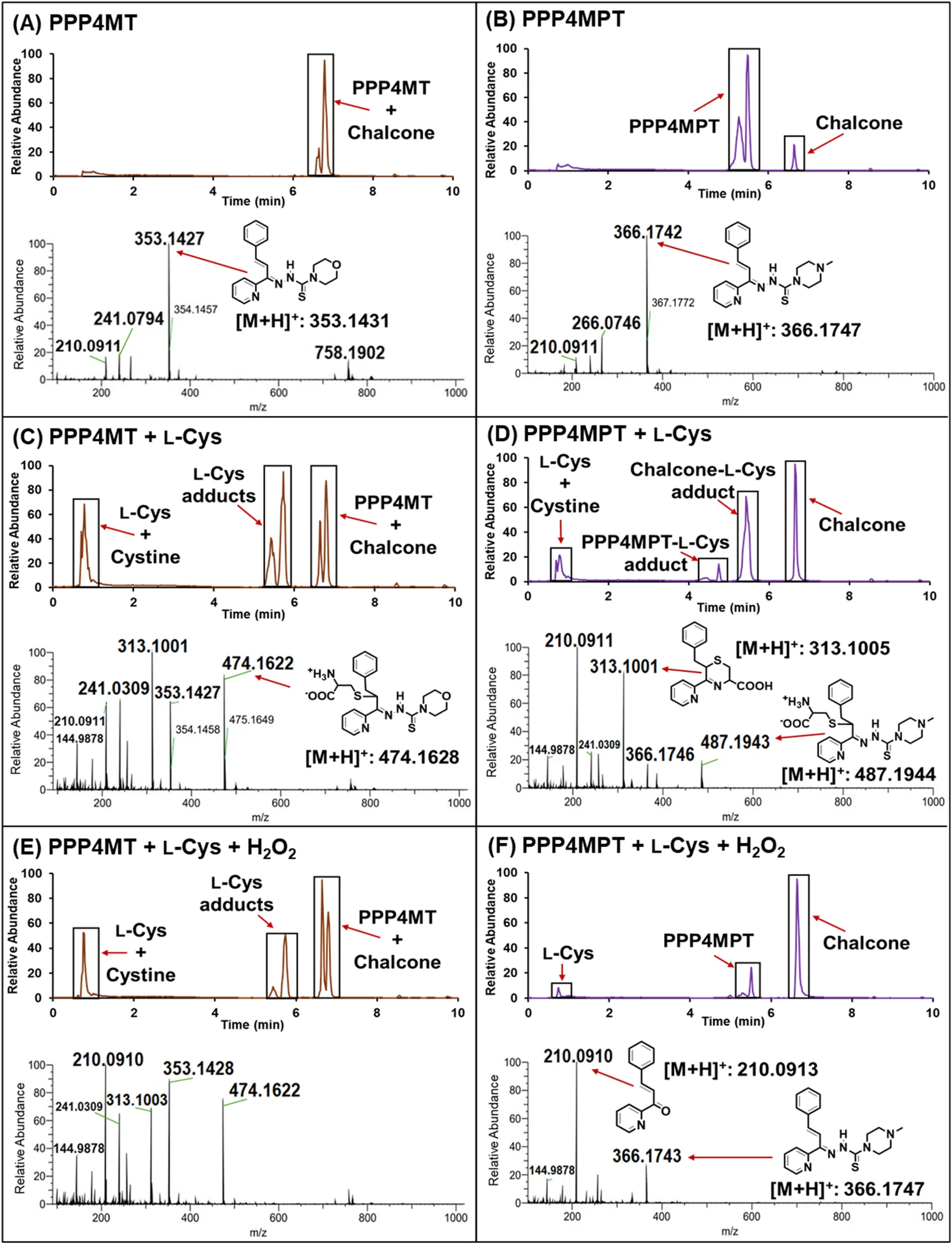
LC-MS analysis of: (A) PPP4MT (500 µM) and (B) PPP4MPT (500 µM) after a 6 h/20 °C incubation in DMSO/H_2_O (7 : 3 v/v); or (C) PPP4MT (500 µM) and (D) PPP4MPT (500 µM) after a 6 h/20 °C incubation in the presence of l-Cys (5 mM) in DMSO/H_2_O (7 : 3 v/v). Following this latter incubation, H_2_O_2_ (10 mM) was added to the respective solutions (E and F), and the incubation continued for 2 h/20 °C. After all incubations, the solutions were diluted 1 : 1 with MeOH prior to LC-MS analysis.

In these studies, LC-MS analysis was also used to define the reaction products underlying the UV-vis changes observed (Fig. 3A–C), and to identify molecular alterations (Fig. 4A–F and 5A–F).^58^ The ligands, PPP44mT, SPPP44mT, PPP4MT, and PPP4MPT, were incubated at 500 µM in DMSO : H_2_O (7 : 3 v/v), in the presence or absence of l-Cys or GSH (5 mM) for 6 h/20 °C.

Following this incubation, the samples were diluted 1 : 1 with methanol (MeOH) and analyzed by LC-MS using a mobile phase consisting of 90% MeOH, 9.9% water, and 0.1% formic acid (Fig. 4 and 5).^45,46^ Notably, LC-MS studies were not feasible under physiologically relevant aqueous conditions, as the ligands could not be dissolved at concentrations sufficient to meet the LC-MS system's detection threshold.

In UV-vis experiments (Fig. 3A), SPPP44mT exhibited well-defined absorption maxima at 307 and 398 nm, and its spectra remained essentially unchanged at both pH 5.0 and 7.4 following the addition of 15-fold excess l-Cys (375 µM). The absence of any appreciable spectral changes suggests that SPPP44mT does not readily react with l-Cys at either lysosomal or cytosolic pH. The LC-MS analysis then further corroborated this lack of reactivity (Fig. 4A). In fact, SPPP44mT eluted as a single LC peak, with a small shoulder, a pattern consistent with a slowly interconverting *E*/*Z* or *syn*/*anti* imine isomers reported for these types of ligands.^46,70^

MS detected the free SPPP44mT ligand (*m*/*z*: 313.1478) as the major species, together with a putative solvent-associated dimer, tentatively assigned as [2M + Na + MeOH − H]^+^ (*m*/*z*: 678.1998), and a minor saturated chalcone fragment (*m*/*z*: 212.1068) arising from limited imine hydrolysis during chromatography (Fig. 4A). Following incubation of SPPP44mT with l-Cys (5 mM), the LC-MS profile of SPPP44mT remained very similar (*cf.*Fig. 4A and B), as observed for the UV-Vis spectrum under aqueous conditions (Fig. 3A). Moreover, no SPPP44mT-l-Cys adduct was detected, with the only additional species being cystine (*m*/*z*: 241.0310; Fig. 4B), consistent with l-Cys oxidation rather than the covalent modification of the ligand.

In contrast to the UV-vis spectrum of SPPP44mT (Fig. 3A), the styryl-containing PPP4HAT ligands, PPP4MT (Fig. 3B), and especially PPP4MPT (Fig. 3C), demonstrated clear spectral changes upon the addition of l-Cys. PPP4MT and PPP4MPT exhibit prominent absorption bands at approximately 330 and 425 nm (Fig. 3B and C), which decrease in intensity upon the addition of l-Cys (375 µM) at pH 7.4. Notably, PPP4MPT with the *N*-methyl-piperazine substituent, displayed a more pronounced hypochromic effect, suggesting greater reactivity towards l-Cys compared to morpholine substituted PPP4MT. In contrast, only minor changes were observed for PPP4MT and PPP4MPT at pH 5.0 (Fig. 3B and C) over the same timescale (6 h). This hypochromism is consistent with a pH-dependent disruption of the conjugated styryl system *via* covalent reaction at the CH=CH bond, in line with a Michael-type addition mechanism.

Considering the greater reactivity of PPP4MPT (Fig. 3C), the kinetics of the reaction between PPP4MPT (25 µM) and l-Cys (250–1000 µM) were then investigated at the physiological temperature of 37 °C at pH 7.4 and pH 5.0 and physiological ionic strength (0.14 M; Table 2 and Fig. S5 and S6). In the absence of l-Cys, the spectrum of PPP4MPT does not change appreciably over an incubation of 6 h/37 °C. In the presence of l-Cys and at pH 7.4 ([l-Cys]/[PPP4MPT] > 10), the characteristic absorption peaks at 335 and 424 nm of the conjugated thiosemicarbazone decrease in intensity, with the final spectrum showing peaks at 316 and 390 (sh) nm (Fig. S5). These spectral changes were fit to a first-order process at each l-Cys concentration (Fig. S6, Table 2) using the program Reactlab KINETICS.^71^

**Table 2:** Observed first-order rate constants (*s*^−1^) for the reaction between PPP4MPT (25 µM) and l-Cys at pH 7.4 and 5.0. Temperature 37 °C and [NaCl] = 0.14 M to mimic physiological conditions

[l-Cys]/µM	pH 7.4	pH 5.0
0	—	—
250	1.40(1) × 10^−4^	6.93(1) × 10^−6^
500	2.74(1) × 10^−4^	1.26(1) × 10^−5^
1000	3.57(1) × 10^−4^	2.01(1) × 10^−5^

Control experiments with the amino acid, l-valine, instead of l-Cys at pH 7.4 elicited no change in the spectrum of PPP4MPT (data not shown). As such, the sulfhydryl sidechain of l-Cys could be responsible for the thiosemicarbazone spectral changes. As mentioned above, a possible explanation is thiolate addition to the conjugated double bond of PPP4MPT. Parallel kinetic studies at pH 5.0 (Table 2) demonstrated that the rate of this reaction decreased by at least an order of magnitude, which is consistent with a mechanism that involves the thiolate form of l-Cys (thiol p*K*_a_ 8.1).

Interestingly, the reactivity of other thiosemicarbazones from this family (*i.e*., PPP4MT) with l-Cys was much lower (Fig. 3). The *N*-methyl-piperazine substituent of PPP4MPT is remote from the styryl group, so the origins of its higher reactivity towards l-Cys relative to the other ligands are not known and will be the subject of future studies.

The strong pH-dependence of these responses in Fig. 3B and C is consistent with the speciation of l-Cys.^72^ At pH 7.4, a greater fraction of l-Cys exists as the nucleophilic thiolate, which can readily attack the α,β-unsaturated styryl CH=CH bond.^73,74^ In contrast, l-Cys is predominantly protonated at pH 5.0, and therefore, significantly less nucleophilic,^73,74^ resulting in a markedly slower reaction. Consequently, the enhanced thiolate population at pH 7.4 accounts for the stronger, hypochromic effects observed for PPP4MT (Fig. 3B), and most prominently, PPP4MPT at pH 7.4 (Fig. 3C). Collectively, these findings strongly support the thiolate-dependent Michael-type addition mechanism, rather than a non-specific spectral perturbation.

#### l-Cysteine adducts of the unsaturated ligand, PPP44mT, undergo a reversible Michael addition–elimination reaction

Considering the finding that the saturated ligand, SPPP44mT, demonstrated no clear thiol reactivity upon l-Cys addition in the LC-MS studies (Fig. 4A and B), analogous studies were then performed with its well-characterized unsaturated analogue, PPP44mT^40^ (Fig. 4C and D). PPP44mT appeared in the chromatogram as a single dominant LC peak with a smaller shoulder at 6.5–7.0 min (Fig. 4C), consistent with slowly interconverting *E*/*Z* imine isomers or *syn*/*anti* conformers (Fig. S3).^46,70^ LC-MS identified the free PPP44mT ligand (*m*/*z*: 311.1321, ∼100%; Fig. 4C) as the major species, together with tentative sodium-associated ligand dimer/solvent cluster (*m*/*z*: 674.1686), and a minor chalcone-like fragment (*m*/*z*: 241.0794), the latter arising from MeOH-associated in-source fragmentation during ionization.

Following incubation with l-Cys (Fig. 4D), two new chromatographic regions appeared in the PPP44mT LC trace, in addition to the ligand's original peak at 6.5–7.0 min (Fig. 4C). The earliest peak at ∼1.0 min (Fig. 4D) consisted of a major component with a very minor shoulder, consistent with unreacted l-Cys and oxidized cystine, corresponding with their high polarity on the C18 column. MS supported these assignments through the detection of an l-Cys-related ion at *m*/*z*: 144.9878 and cystine (*m*/*z*: 241.0310; Fig. 4D). The second region in the chromatogram at 5.5–5.9 min represented the major thiol-derived reaction products and included both the PPP44mT-l-Cys Michael adduct and a cyclized chalcone-l-Cys species, identified by dominant signals at *m*/*z*: 432.1519 and *m*/*z*: 313.1003, respectively (Fig. 4D). The free ligand (*m*/*z*: 311.1321) and its putative sodium/MeOH associated dimer (*m*/*z*: 674.1687) also remained detectable at 6.5–7.0 min, indicating co-existence of unreacted PPP44mT with multiple l-Cys -modified products (Fig. 4D). Minor variations in peak shape within this 6.5–7.0 min window (*cf.*Fig. 4C and D) likely reflect closely related l-Cys-adduct species that share the same *m*/*z* values but differ slightly in chromatographic behavior.

It was previously demonstrated that l-Cys adducts formed by Michael addition in other molecules exhibit high reactivity toward hydrogen peroxide (H_2_O_2_), undergoing a reversible Michael addition–elimination reaction.^75,76^ On this basis, we hypothesized that exposure of the PPP44mT-l-Cys adduct to H_2_O_2_ would promote oxidative cleavage of the C–S bond and regenerate the free ligand.^69^ To investigate this, SPPP44mT (Fig. 4E) and PPP44mT (Fig. 4F) were incubated with H_2_O_2_ (10 mM, 2 h/20 °C) following the initial 6 h/20 °C incubation with l-Cys.

For the saturated SPPP44mT analogue incubated with l-Cys, no appreciable changes in the LC trace or the MS were observed upon H_2_O_2_ treatment (Fig. 4E) relative to SPPP44mT incubated with l-Cys but not treated with H_2_O_2_ (Fig. 4B). This result was consistent with the inability of SPPP44mT to form l-Cys adducts, resulting in no UV-vis spectral alterations (Fig. 3A). In contrast, the addition of H_2_O_2_ to unsaturated PPP44mT incubated with l-Cys induced a decrease in the intensity of the l-Cys adduct peak at 5.5–5.9 min in the LC trace (Fig. 4F). This comparison is made relative to PPP44mT incubated with l-Cys, but no H_2_O_2_ (Fig. 4D). In fact, upon adding H_2_O_2_, MS analysis confirmed decreased levels of the PPP44mT-l-Cys adduct (*m*/*z*: 432.1518) and the cyclized chalcone-l-Cys species (*m*/*z*: 313.1001; *cf.*Fig. 4D and F). This latter change was accompanied by persistent signals consistent with the sodium adduct of l-Cys and/or its isotope cluster around *m*/*z* 144–145, cystine (*m*/*z*: 241.0309), and the free ligand (*m*/*z*: 311.1322; Fig. 4F). These alterations indicate partial oxidative reversal of the PPP44mT-l-Cys adduct and recovery of unmodified PPP44mT.

Considering the results above with PPP44mT, further LC-MS analysis examined the chemical species underlying the UV-vis changes observed for PPP4MT (Fig. 3B) and PPP4MPT (Fig. 3C). In these studies, the latter ligands were examined after incubation with or without l-Cys (6 h/20 °C; Fig. 5A–F). In the absence of l-Cys, PPP4MT gave a single LC trace with a small shoulder at 6.2–7.0 min (Fig. 5A), consistent with co-elution of PPP4MT and residual phenyl–chalcone. The corresponding MS showed the protonated ligand as the dominant species (*m*/*z*: 353.1427), together with phenyl–chalcone (*m*/*z*: 210.0911), a chalcone-related fragment (*m*/*z*: 241.0794), and a putative [2M + Na + MeOH − H]^+^ solvent-associated ligand dimer (*m*/*z*: 758.1902).

PPP4MPT displayed two closely migrating LC traces at 5.2–5.8 min (Fig. 5B), attributable to interconverting *E*/*Z* and *syn*/*anti* imine conformers of the ligand.^46,70^ These were followed by a single later LC trace at ∼7.0–7.5 min assigned to phenyl–chalcone. The MS confirmed these assignments with signals for PPP4MPT (*m*/*z*: 366.1747) and protonated chalcone (*m*/*z*: 210.0911), generated *in situ* under ESI conditions. A fragment ion at *m*/*z* 266.0746, consistent with a cyclized ligand fragment generated following loss of the *N*-methyl piperazine substituent, was also observed.

Thiosemicarbazones are generally stable and do not undergo spontaneous imine cleavage,^46^ so the unexpected detection of free phenyl–chalcone in the LC-MS chromatograms (Fig. 4 and 5) required further investigation. To determine whether chalcone cleavage occurs prior to LC-MS analysis or during chromatographic processing, UV-vis spectra of PPP4MPT (25 µM), and the parent chalcone were recorded under LC-MS-matched conditions (Fig. S7). Spectra were compared at 0 h and after 6 h/20 °C in DMSO/water (7 : 3 v/v). The solutions were then subjected to a 1 : 1 dilution either with (W/) or without (W/O) the LC-MS mobile phase (90% MeOH, 9.9% water, 0.1% formic acid), and incubated for an additional 10 min/20 °C.

At both 0 h and 6 h, without the mobile phase, the spectra showed no appreciable alterations. PPP4MPT consistently displayed its characteristic π → π* transition at 304 nm and the intra-ligand charge-transfer band at 453 nm, with no features indicative of chalcone formation (Fig. S7). After the 10 min/20 °C incubation with the mobile phase, the intra-ligand charge-transfer band exhibited a small but reproducible blue shift of ∼7 nm (453 → 446 nm). This subtle shift may reflect a minor solvatochromic effect or a slight spectral contribution from the chalcone, which absorbs as a single peak at 320 nm (Fig. S7). Importantly, the overall spectral profile of PPP4MPT did not evolve toward the characteristic chalcone spectrum, demonstrating that the imine linkage remains largely intact under LC-MS solvent conditions.

Collectively, these results indicate that PPP4MPT does not release chalcone in solution and remains stable during sample preparation. Thus, the chalcone peak observed in the LC-MS chromatograms most likely originate during the chromatographic processing itself, potentially through partial imine hydrolysis or MeOH-associated fragmentation on the C18 column.

After incubation with l-Cys (5 mM), PPP4MT showed three LC regions (Fig. 5C). An early doublet at ∼1.0 min corresponded to l-Cys and cystine, as indicated by a l-Cys-related ion (*m*/*z*: 144.9878) and oxidized cystine (*m*/*z*: 241.0309). A new cluster of LC peaks at 5.5–5.9 min represented l-Cys addition products, dominated by the cyclized chalcone-l-Cys adduct (*m*/*z*: 313.1001) and the PPP4MT-l-Cys Michael adduct (*m*/*z*: 474.1628). The original 6.5–7.0 min peak and shoulder in Fig. 5A of PPP4MT were retained in Fig. 5C and was consistent with unreacted PPP4MT (*m*/*z*: 353.1427), together with phenyl–chalcone (*m*/*z*: 210.0911), indicating partial conversion and co-existence of ligand and adducts.

For PPP4MPT, incubation with l-Cys resulted in four distinct LC traces (Fig. 5D). The doublet at ∼1.0 min was consistent with sodium-associated l-Cys species and cystine (*m*/*z*: 144.9878 and 241.0309). A smaller peak set at 4.2–4.8 min contained the PPP4MPT-l-Cys adduct (*m*/*z*: 487.1943), followed at ∼5.5 min by the dominant cyclized chalcone-l-Cys species (*m*/*z*: 313.1001; Fig. 5D). The final peak at ∼6.6 min was consistent with the free phenyl-chalcone (*m*/*z*: 210.0911), with only a minor contribution from unreacted PPP4MPT (*m*/*z*: 366.1746; Fig. 5D). The much weaker free-ligand peak for PPP4MPT (*m*/*z*: 366.1746) relative to PPP4MT (*m*/*z*: 353.1427; *cf.*Fig. 5C and D) indicated its greater propensity to form l-Cys adducts, consistent with the more robust UV-vis change of PPP4MPT *versus* PPP4MT at pH 7.4 (*cf.*Fig. 3B and C).

To examine the reversibility of adduct formation observed in Fig. 5C and D, H_2_O_2_ (10 mM) was added for 2 h/20 °C after the initial 6 h/20 °C incubations of PPP4MT and PPP4MPT with l-Cys (Fig. 5E and F). For PPP4MT, the 5.5–5.9 min adduct region decreased in intensity (*cf.*Fig. 5C and E), with MS demonstrating decreased PPP4MT-l-Cys adduct (*m*/*z*: 474.1622) and chalcone-l-Cys adduct (*m*/*z*: 313.1003) signals. Persistence of cystine (*m*/*z*: 241.0309) with the regeneration of the free ligand (*m*/*z*: 353.1428) and chalcone (*m*/*z*: 210.0910) was evident (Fig. 5E). In the PPP4MPT system treated with H_2_O_2_ (Fig. 5F), the LC traces of both the PPP4MPT-l-Cys adduct and chalcone-l-Cys adducts were lost (*cf.*Fig. 5D and F), leaving three main peaks assigned to l-Cys-related ion (*m*/*z*: 144.9878), free phenyl-chalcone (*m*/*z*: 210.0910), and free PPP4MPT (*m*/*z*: 366.1747). The complete disappearance of l-Cys adducts for PPP4MPT (Fig. 5F), *versus* only partial loss for PPP4MT (Fig. 5E), further supports the higher reactivity and fully reversible Michael chemistry of PPP4MPT under oxidative conditions.

Notably, when the ligands themselves, namely SPPP44mT, PPP44mT, PPP4MT, and PPP4MPT, were incubated with H_2_O_2_ (10 mM) for 2 h/20 °C, minor traces of the products generated *via* desulfurization followed by intermolecular ligand cyclization were observed (Fig. S8A–D). This might be due to the high H_2_O_2_ concentration (10 mM) used. The HPLC traces of these desulfurized products were barely detectable with SPPP44mT (<4%; Fig. S8A) and PPP44mT (<2%; Fig. S8B). In contrast, these by-products were much more prominent with PPP4MT (Fig. S8C), and particularly PPP4MPT (Fig. S8D). Considering PPP4MT, the desulfurized product was found to exist in two tautomeric forms (*m*/*z*: 319.1548 ∼7% and *m*/*z*: 321.1705 ∼5%) following cyclization (Fig. S8C). In the case of PPP4MPT (Fig. S8D), the tautomeric form lacking the double bond predominated (*m*/*z*: 334.2021, ∼17%) over the form with the double bond (*m*/*z*: 332.1867, ∼1%), which was barely detectable.

Subsequently, the reactivity of the ligands with GSH (5 mM; Fig. S9A–D) was examined under identical conditions to those examining l-Cys adduct formation after a 6 h/20 °C incubation.^69^ Neither SPPP44mT, PPP44mT, PPP4MT, nor PPP4MPT formed detectable GSH adducts (Fig. S9A–D), with only the free ligands and unreacted GSH present. This lack of reactivity likely reflects the lower acidity (p*K*_a_ = 8.7), and, therefore, reduced thiolate population of GSH *versus*l-Cys (p*K*_a_ = 8.1).^69,77^ Together with the greater steric bulk of GSH, these factors may limit its reactivity with the α,β-unsaturated bond of PPP44mT, PPP4MT, and PPP4MPT under the present experimental conditions.^77,78^

Collectively, these results in Fig. 3–5 reveal that the preserved styryl double bond is a key determinant of thiol reactivity across multiple thiosemicarbazone generations.^40,42,46^ Notably, PPP4MPT exhibits the highest susceptibility to Michael addition, which may be speculated to contribute to its superior anti-proliferative activity (Table 1 and S3). Although, the *N*-methyl-piperazine substituent is distal to the styryl group, it may indirectly influence l-Cys reactivity by altering conformational preference, protonation/solvation behavior, or electron distribution across the conjugated thiosemicarbazone framework. Despite comprehensive attempts, the cysteine adducts suffered rapid decomposition and limited solubility, which precluded characterization *via* NMR spectroscopy.

Previous studies examining chalcones with an α,β-unsaturated ketone moiety have reported that they modulate the activity of proteins such as nuclear factor kappa-light-chain-enhancer of activated B cells (NF-κB), Kelch-like ECH-associated protein 1 (Keap1)/nuclear factor erythroid 2-related factor 2 (Nrf2), and thioredoxin (Trx) *via* a Michael-type reaction.^79–83^ Certain chalcones have also demonstrated inhibition of pro-oncogenic NF-κB by modifying the cysteine residues of IκB kinases, which are a key regulator of NF-κB.^79,81^ Trx is a known anti-apoptotic protein, and a chalcone derivative can modify the selenocysteine residue in Trx reductase.^83,84^ However, future studies beyond the scope of this investigation will need to examine the biological relevance and activity of these α,β-unsaturated styryl-containing thiosemicarbazones.

#### Generation of ROS by 1 : 1 ligand : Cu(ii) PPP4HAT complexes

Given the marked anti-proliferative activity observed with these PPP4HAT analogues and their 1 : 1 Cu(ii) complexes (Table 1 and S3), their ability to generate reactive oxygen species (ROS) was investigated in the context of the established ‘double punch’ mechanism of thiosemicarbazones.^42,48,50,85^ This mechanism involves intracellular metal binding followed by Cu-dependent redox cycling, leading to cytotoxic ROS generation.^42,50,85^ Detailed experimental conditions and figures are provided in the SI (Fig. S10A–C).

Extracellular DCF assays (Fig. S10A and B) demonstrated that the free ligands were redox-inactive under both cytosolic (pH 7.4) and lysosomal (pH 5.0) conditions, whereas all 1 : 1 ligand:Cu(ii) complexes induced significant ROS generation in a Cu-dependent manner, as confirmed by suppression with the Cu chelator, tetrathiomolybdate (TM). Among the PPP4HAT series, the Cu(ii) complex of the methyl-piperazine analogue, [Cu(PPP4MPT)Cl], consistently produced the highest levels of ROS, particularly under lysosomal conditions, where its activity exceeded that of the established positive control, [Cu(DpC)Cl_2_]^40,42,50^ (Fig. S10A and B). Correlation analysis revealed a moderate positive relationship (*r* = 0.65 and 0.61 at pH 5.0 and 7.4, respectively) between extracellular ROS generation and anti-proliferative activity at 24 h, which was markedly decreased (*r* = −0.33 and −0.28 at pH 5.0 and 7.4, respectively) after a 72 h incubation (Fig. S11A–D). This likely reflects that, over 72 h, several Cu(ii) complexes reach comparable cytotoxic potency, masking early differences in ROS generation, rather than being interpreted as a loss of redox activity.

In contrast to the extracellular assays, intracellular DCF measurements in MCF-7 cells showed only very modest differences in ROS levels between individual Cu(ii) complexes (Fig. S10C). All Cu(ii) species induced a small but significant (*p* < 0.0001–0.001) increase in intracellular ROS relative to the respective ligand, which was significantly (*p* < 0.001) suppressed by TM (Fig. S10C). However, the magnitude of these changes was substantially lower than observed extracellularly (Fig. S10A, B) and showed poor correlation (*r* = 0.35 after 24 h and *r* = 0.23 after 72 h; data not shown) with anti-proliferative activity. This likely reflects efficient intracellular antioxidant buffering, including GSH and a battery of enzymatic ROS-scavenging systems.^86,87^

Collectively, these data demonstrate that the PPP4HAT Cu(ii) complexes are capable of effective Cu-dependent redox cycling, with [Cu(PPP4MPT)Cl], the Cu complex of the most potent ligand in the series (Table 1 and S3), showing prominent activity under extracellular conditions (Fig. S10A and B). However, the weak correlation between intracellular ROS levels and cytotoxicity indicates that ROS generation represents only one component of a multifactorial anti-cancer mechanism, which includes essential metal deprivation and modulation of proliferation-associated signaling pathways (see Fig. 7A and B).^28,34,50,88^

#### Oxy-Mb oxidation by Fe(iii) complexes of PPP4HAT analogues

Oxidation of oxy-Mb to deleterious met-Mb was a major drawback of the previous DpT series of ligands, including Dp44mT and DpC.^40,41^ The incorporation of a styrene moiety in the PPPT series lowered the oxidation levels compared to the DpT series ligands.^40^ Since the PPP4pT analogues, and particularly the PPP4pSe series, significantly attenuated the formation of detrimental met-Mb,^42,45^ it is of interest to investigate how structural modifications of the novel PPP4HAT analogues influence oxidation of oxy-Mb.

**Fig. 6:**
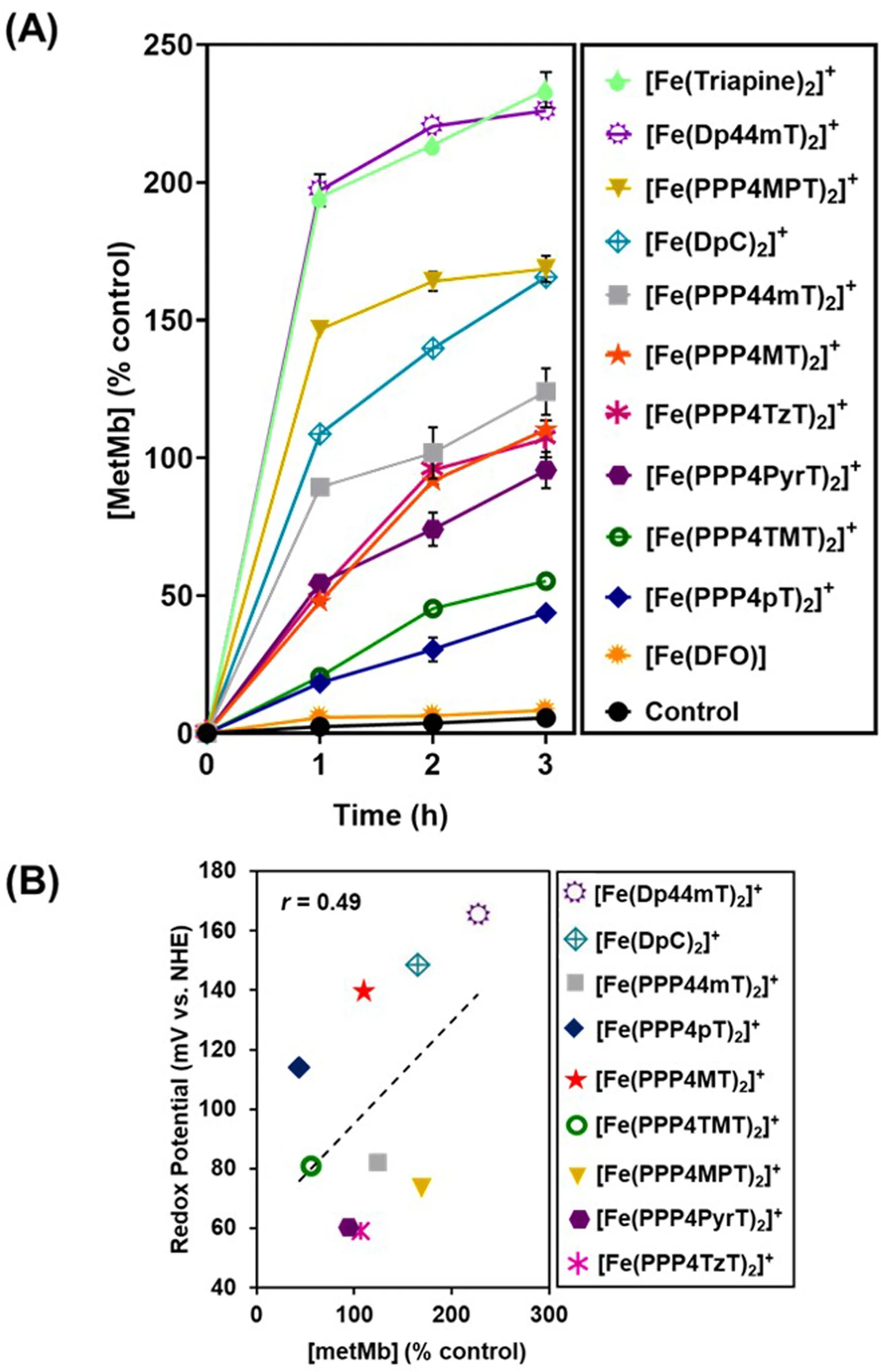
(A) The majority of 2 : 1 L : Fe(iii) complexes of the PPP4HAT analogues demonstrate appreciably less activity at oxidizing oxy-Mb (40 µM) to met-Mb than the 2 : 1 ligand: Fe(iii) complexes of Triapine, Dp44mT, and DpC. (B) The correlation between redox potential and met-Mb formation (% control) following a 3 h/20 °C incubation of oxy-Mb and the Fe(iii) complexes of the PPP4HAT analogues. Results are mean ± SEM (3 experiments).

The ability of the Fe(iii) complexes of the PPP4HAT analogues to inhibit oxy-Mb oxidation was compared to [Fe(DFO)], which was used as the negative control, along with Fe(iii) complexes of earlier thiosemicarbazones, Triapine, Dp44mT, DpC, PPP44mT, and PPP4pT, as the positive controls (Fig. 6A).^40–42^ In these studies, oxy-Mb was used preferentially to oxy-Hb due to its greater sensitivity to the oxidation by Fe(iii) complexes of thiosemicarbazones.^40–42,45^ Studies were performed using our established conditions for comparison^40–42,45^ by incubating purified oxy-Mb (40 µM) with the compounds (10 µM) for 3 h/20 °C, measuring the absorbance each hour (0–3 h).

As anticipated, redox inactive [Fe(DFO)] did not cause a significant level of oxidation compared to the control.^40–42,45^ Of the positive controls, [Fe(Triapine)_2_]^+^ induced the highest level of oxidation, comparable to [Fe(Dp44mT)_2_]^+^, which oxidized oxy-Mb to >225% of the control after 3 h/20 °C (Fig. 6A). In comparison, oxy-Mb oxidation induced by [Fe(DpC)_2_]^+^ was significantly (*p* < 0.001) lower (165% of the control) than [Fe(Dp44mT)_2_]^+^, whereas [Fe(PPP44mT)_2_]^+^ lowered the oxidation level to 125% of the control after 3 h/20 °C (Fig. 6A). Among the thiosemicarbazones examined, [Fe(PPP4pT)_2_]^+^, induced the lowest oxidation, which was <50% of the control after 3 h/20 °C.

PP4MPT was identified as the lead analogue of the PPP4HAT series based on its potent anti-proliferative activity (Table 1 and S3), together with the pronounced redox activity of its Cu(ii) complex (Fig. S10). Despite emerging as the most promising analogue in these studies, the corresponding Fe(iii) complex, [Fe(PPP4MPT)_2_]^+^, demonstrated oxy-Mb oxidation levels similar to [Fe(DpC)_2_]^+^ after a 3 h incubation (Fig. 6A), representing the highest oxy-Mb oxidation within the PPP4HAT series. Further, [Fe(PPP4MT)_2_]^+^ and [Fe(PPP4TzT)_2_]^+^ induced oxy-Mb oxidation comparable to the third generation [Fe(PPP44mT)_2_]^+^, whereas [Fe(PPP4PyrT)_2_]^+^, demonstrated slight but significantly (*p* < 0.05) lower oxy-Mb oxidation (Fig. 6A). The Fe(iii) complex of thiomorpholine analogue, [Fe(PPP4TMT)_2_]^+^, caused the lowest oxy-Mb oxidation (55% of the control) amongst the PPP4HAT analogues after 3 h/20 °C incubation, which was similar to [Fe(PPP4pT)_2_]^+^ (Fig. 6A).

Considering the relationship between the oxy-Mb oxidation *versus* the redox potentials of these Fe(iii) complexes, a positive correlation (*r* = 0.49) was observed (Fig. 6B). Previous findings for structurally related PPP44mT analogues demonstrated that the Fe(iii)/Fe(ii) redox potentials measured under aqueous conditions were positively correlated with oxy-Mb oxidation.^40,42^ In contrast, for the PPP4pT analogues, no correlation was observed, potentially due to the use of DMF as the supporting solvent in cyclic voltammetry.^40,42^ The DMF was used due to the lower solubility of the PPP4pT analogues. DMF alters ligand protonation states, metal–ligand speciation, and coordination equilibria relative to aqueous buffers. These solvent-dependent changes disrupt the physiological Fe(iii)/Fe(ii) redox couple, shifting the apparent potential, masking the intrinsic redox properties that govern biological oxidation. Therefore, the loss of correlation may reflect solvent effects rather than intrinsic redox properties.

The findings of these studies indicated that the structural modifications of the novel PPP4HAT series are suboptimal in terms of their ability to prevent the formation of deleterious met-Mb. Due to the replacement of the bulky phenyl group present in the fourth (Fig. 1F) and fifth (Fig. 1G) generation agents with smaller heterocyclic amines, the PPP4HAT analogues are structurally more similar to the third-generation PPP44mT series (Fig. 1E). Hence, the steric hindrance imposed by the PPP4HAT series analogues may be comparatively weaker, leading to increased oxy-Mb oxidation.

To gain further qualitative structural insights into predicted orientation and steric accessibility relative to the oxy-Mb heme pocket, molecular docking studies were performed for the Fe(iii) complexes of the new PPP4HAT series (Table S5 and Fig. S12). These docking simulations suggest that both electronic and steric features of the terminal amine may critically influence Fe(iii) complex interactions with oxy-Mb. Complexes featuring small, flexible, and electron-rich substituents (*e.g.*, methyl-piperazine, PPP4MPT) were predicted to access the heme edge more readily, potentially permitting closer interactions with the heme environment (Table S5 and Fig. S12). In contrast, rigid substituents (*e.g.*, thiomorpholine, PPP4TMT), appeared to hinder the approach to the heme surface, consistent with their higher binding energy and reduced reactivity. The observed trends of the molecular docking simulations may provide a qualitative structural framework that is consistent with the *in vitro* observations.

#### Effect of PPP4HAT analogues on the expression of major molecular targets in breast cancer cells

**Fig. 7:**
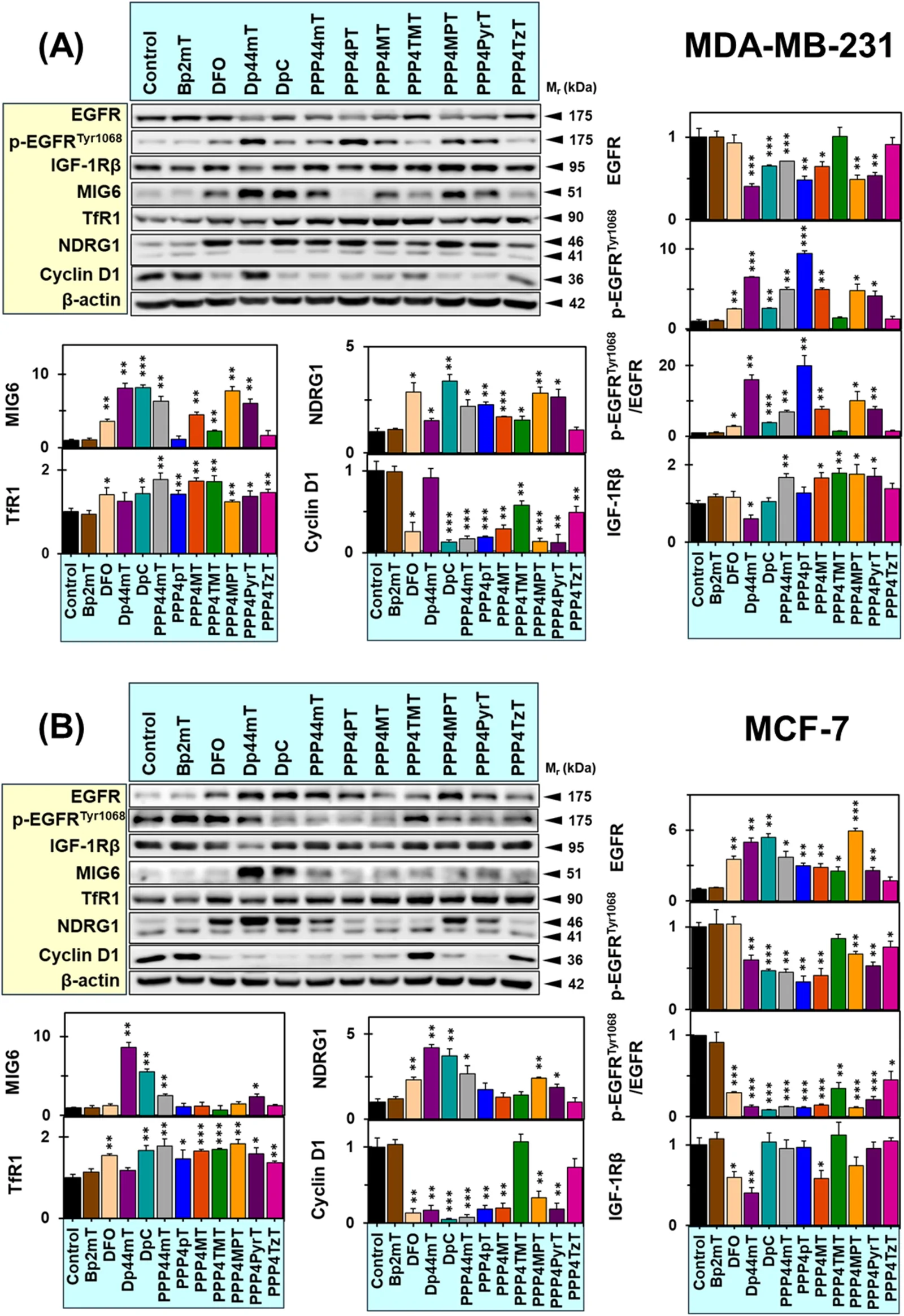
(A and B) Western analysis examining the effect of a 24 h/37 °C incubation with the PPP4HAT analogues (5 µM) relative to DFO (100 µM), Dp44mT (5 µM), DpC (5 µM), PPP44mT (5 µM), and PPP4pT (5 µM) on EGFR, *p*-EGFR^Tyr1068^, IGF-1Rβ, MIG6, TfR1, NDRG1, and cyclin D1, expression in: (A) MDA-MB-231 and (B) MCF-7 BC cells. The blots are from 3 experiments. The densitometry values are presented as mean ± SD (*n* = 3). *p < 0.05; **p < 0.01; ***p < 0.001.

Given the pronounced anti-proliferative activity of these novel morpholine derivatives in breast cancer cells (Table 1), it was important to elucidate their mechanism of action in these cell types. Hence, western blot analysis was performed comparing TNBC cells (MDA-MB-231) and luminal A breast cancer cells (MCF-7) incubated with the novel PPP4HAT analogues for 24 h/37 °C (Fig. 7A and B). The effects of these analogues on key protein expression were compared with 2-benzoylpyridine-2-methyl-3-thiosemicarbazone (Bp2mT; 5 µM), which was designed as a negative control that cannot bind metal ions,^89^ as well as with DFO (100 µM), Dp44mT, DpC, PPP44mT, and PPP4pT (all at 5 µM) as positive controls.

The regulation of receptor tyrosine kinases such as epidermal growth factor receptor (EGFR), and insulin-like growth factor 1 receptor β (IGF-1Rβ) by the PPP4HAT analogues was evaluated along with key proteins that regulate proliferation, including transferrin receptor 1 (TfR1), mitogen-inducible gene 6 (MIG6), NDRG1, and cyclin D1 (Fig. 7).^21,22^ The negative control, Bp2mT, did not significantly (*p* > 0.05) affect the expression of any of the proteins examined across both breast cancer cell types relative to the control (Fig. 7).

EGFR plays an important role in regulating critical aspects of cancer progression, such as proliferation, metastasis, and resistance to drugs, and it is known to be overexpressed in around 50% TNBC cases.^90,91^ Hence, the significant (*p* < 0.001–0.05) down-regulation of EGFR in MDA-MB-231 TNBC cells by Dp44mT, DpC, and all the PPP4HAT ligands (except PPP4TMT and PPP4TzT) was a desirable result (Fig. 7A). However, relative to the control, a significant (*p* < 0.001–0.05) increase in EGFR phosphorylation (*p*-EGFR) at Tyr1068 and the *p*-EGFR to total EGFR ratio was observed for all ligands except PPP4TMT and PPP4TzT (Fig. 7A). This latter effect may be speculated to relate to these agents forming redox-active complexes, as EGFR phosphorylation has been reported to increase after redox stress.^92,93^ However, this does not readily explain the decreased EGFR phosphorylation by the same compounds in MCF-7 cells (Fig. 7A), suggesting cell type molecular heterogeneity plays a role in the effect.

The response of EGFR and *p*-EGFR levels to the ligands in MCF-7 cells (Fig. 7B) demonstrated a generally opposite response to those in MDA-MB-231 cells (Fig. 7A). Total EGFR expression was significantly (*p* < 0.001–0.05) up-regulated by the majority of the PPP4HAT series except PPP4TzT (Fig. 7B). In contrast, phosphorylation of EGFR at Tyr1068 was significantly (*p* < 0.001–0.05) decreased relative to the control by the majority of the PPP4HAT ligands except PPP4TMT (Fig. 7B). This was reflected by the evaluation of the *p*-EGFR/total EGFR ratio, which exhibited a significant (*p* < 0.001–0.05) decrease by all the ligands compared to the control and negative control, Bp2mT (Fig. 7B). These results are consistent with previous studies examining EGFR and *p*-EGFR(Tyr1068) levels in MCF-7 cells.^21^

Prior research has explored this paradoxical phenomenon of EGFR expression between different subtypes of breast cancer.^94,95^ It has been reported that low expression of EGFR in ER-positive/HER2-negative breast cancer tumors is associated with poor outcomes, while high expression of EGFR in these tumors leads to better patient survival, which is not seen with TNBC.^95^ Considering the fact that MCF-7 cells belong to the luminal A subtype, which is ER-positive/HER2-negative, the up-regulation of EGFR by the ligands may be a favorable anti-tumor response.^95^ However, further studies beyond the scope of this investigation are required to examine this further.

Similar to EGFR, IGF-1Rβ is a key player in cancer progression, contributing to proliferation and migration.^96,97^ The expression of IGF-1Rβ was significantly (*p* < 0.05) down-regulated by Dp44mT in MDA-MB-231 cells (Fig. 7A). In contrast, PPP44mT and four of the PPP4HAT analogues, namely PPP4MT, PPP4TMT, PPP4MPT, and PPP4PyrT, significantly (*p* < 0.01–0.05) up-regulated IGF-1Rβ expression in MDA-MB-231 TNBC cells (Fig. 7A). However, examining MCF-7 cells, DFO, Dp44mT, and PPP4MT significantly (*p* < 0.01–0.05) down-regulated IGF-1Rβ compared with the control (Fig. 7B). These observations generally align with previous studies exploring the effect of Dp44mT and DpC in MCF-7 cells.^21^ Further studies are required to elucidate the up-regulation of IGF-1Rβ by most of the PPP4HAT analogues in MDA-MB-231 TNBC cells (Fig. 7A), despite the anti-proliferative activity of these agents.

In MDA-MB-231 cells, the expression of MIG6 was significantly (*p* < 0.001–0.01) up-regulated by a majority of the compounds, including DFO, Dp44mT, DpC, PPP44mT, PPP4MT, PPP4TMT, PPP4MPT, and PPP4PyrT (Fig. 7A). In contrast, examining MCF-7 cells (Fig. 7B), MIG6 was only significantly (*p* < 0.01–0.05) up-regulated by Dp44mT, DpC, PPP44mT, and PPP4PyrT. Given that MIG6 is a tumor suppressor and negative feedback regulator of EGFR protein levels,^98^ a negative correlation between MIG6 and EGFR levels was expected. Although this relationship was generally observed with MDA-MB-231 cells (Fig. 7A), the correlation between EGFR and MIG6 was less pronounced in MCF-7 cells, suggesting more complex regulation (Fig. 7B).

Expression of the TfR1, which is involved in iron uptake from transferrin and is regulated by cellular iron levels,^99^ was significantly (*p* < 0.001–0.05) up-regulated by all ligands except Dp44mT in both breast cancer cell types, indicating their effective iron-chelating capabilities. The metastasis suppressor, NDRG1, is regulated by cellular iron levels and is well known as a molecular target of thiosemicarbazones.^88,100,101^ In MDA-MB-231 cells, NDRG1 was significantly (*p* < 0.001–0.05) up-regulated by all ligands compared to the control, except PPP4TzT (Fig. 7A). In contrast, only DFO, Dp44mT, DpC, PPP44mT, PPP4MPT, and PPP4PyrT significantly (*p* < 0.01–0.05) up-regulated NDRG1 in MCF-7 cells *versus* the control (Fig. 7B). The up-regulation of NDRG1 is particularly important, given its role in suppressing multiple oncogenic signaling pathways.^88,100,101^ These include the inhibition of WNT signaling, which can down-regulate pro-proliferative cyclin D1 levels.^88,100,101^

Cyclin D1 expression was significantly (*p* < 0.001–0.05) down-regulated in both breast cancer cell types by the majority of the ligands examined (Fig. 7A and B). Assessing MDA-MB-231 cells (Fig. 7A), cyclin D1 expression was not significantly (*p* > 0.05) affected by Dp44mT, which may partly reflect its minimal anti-proliferative activity in this cell type after 24 h (Table 1). Furthermore, cyclin D1 expression was not significantly (*p* > 0.05) down-regulated by PPP4TMT and PPP4TzT in MCF-7 cells (Fig. 7B). The variable regulation of major molecular targets by the PPP4HAT analogues across the two breast cancer cell types probably reflects the molecular heterogeneity between TNBC cells (MDA-MB-231) and ER-positive luminal A cells (MCF-7).

## Conclusions

Recent studies examining thiosemicarbazone medicinal chemistry have uncovered key structure–activity relationships.^40,42,46^ The current investigation assessed the novel sixth-generation PPP4HAT thiosemicarbazone series, focusing on their structure–activity relationships and biological potency. Of this series, the methyl-piperazine-derived analogue, PPP4MPT, demonstrated the highest overall anti-proliferative activity against breast and lung cancer cell types after a 24 h incubation. In fact, depending on the cell type, PPP4MPT demonstrated activity comparable to, or significantly greater than, that of the highly potent third-generation analogue, PPP44mT.^40^

The more aggressive MDA-MB-231 TNBC and DMS-53 SCLC cell types were significantly more resistant to the PPP4HAT analogues compared to the less aggressive luminal A MCF-7 cells and H1299 NSCLC cells. After a 72 h incubation with the cells, in addition to PPP4MPT, both PPP4MT and PPP4PyrT exhibited marked anti-proliferative efficacy among the PPP4HAT series. In contrast, the thiomorpholine analogue, PPP4TMT, demonstrated the lowest activity. In terms of their mechanism of action, the majority of the PPP4HAT analogues also up-regulated the tumor suppressor, MIG6, and the metastasis suppressor, NDRG1, while down-regulating pro-oncogenic cyclin D1.

As the earlier generation of thiosemicarbazones, namely the PPP4pT series,^42^ and PPP4pSe series,^45^ possessed poor solubility, the novel PPP4HAT series was designed with hydrophilic, non-aromatic heterocyclic substitutions. Assessment of the physicochemical properties of the PPP4HAT ligands revealed that all complied with Lipinski's rules, indicating favorable bioavailability. The lower log *P*_calc_ values of the PPP4HAT analogues indicated decreased lipophilicity and consequently improved solubility. These findings highlight the success of this small-molecule design strategy in delivering enhanced anti-cancer potency and superior solubility compared to previous thiosemicarbazone generations.^42,45^

Surprisingly, the design of these novel PPP4HAT analogues resulted in less effective activity in inhibiting the oxidation of oxy-Mb. Of this series, the lowest met-Mb generation was exhibited by the Fe(iii) complex of PPP4TMT, which was comparable to the fourth-generation complex, [Fe(PPP4pT)_2_]^+^. The Fe(iii) complexes of PPP4MT, PPP4PyrT, and PPP4TzT induced oxy-Mb oxidation levels comparable to the third-generation, PPP44mT Fe(iii) complex. Further, the Fe(iii) complex of the most potent methyl-piperazine analogue, PPP4MPT, demonstrated oxy-Mb oxidation levels comparable to second-generation [Fe(DpC)_2_]^+^. Molecular docking simulations suggested the CH_2_–CH_2_ substituents in the heterocyclic rings, together with the reduced steric bulk of these analogues, particularly PPP4MPT, may favor a ligand orientation that permits a closer approach to the heme plane. Hence, despite their pronounced anti-cancer activity and enhanced solubility, the PPP4HAT series were unable to surpass the fourth- and fifth-generation compounds in inhibiting oxy-Mb oxidation.^42,45^

For the first time, we demonstrate that l-Cys reacts directly with thiosemicarbazones bearing an α,β-unsaturated double bond in the styryl moiety that may have biological relevance for modulating protein activity.^79–83^ LC-MS analysis revealed that l-Cys undergoes Michael addition in PPP44mT and the newly developed PPP4MT and PPP4MPT ligands, resulting in covalent ligand–l-Cys thioether adducts. In contrast, the saturated analogue, SPPP44mT (CH_2_–CH_2_), did not form an l-Cys adduct, confirming the α,β-unsaturated bond (CH=CH) is essential for this reactivity.

These l-Cys adducts are redox-responsive, with H_2_O_2_ cleaving the C–S bond and regenerating the free ligand. This reversible thiol-Michael addition establishes an equilibrium between the l-Cys adduct and the intact ligand. The dynamic regeneration of the active ligand species may enhance the effective chelation of Fe(iii) and Cu(ii) and promote the overall redox capacity under biological conditions. This effect could underpin the pronounced anti-proliferative efficacy observed, particularly for PPP4MPT (Table 1 and S3). In fact, the PPP4MPT Cu(ii) complex, [Cu(PPP4MPT)Cl], displayed the highest extracellular redox activity of all the PPP4HAT series ligands at pH 5.0 and particularly pH 7.4 (Fig. S10A and B). Consistent with this observation, PPP4MPT showed nearly complete formation of the l-Cys adduct with full reversal upon H_2_O_2_ treatment (*cf.*Fig. 5D and F).

In contrast to PPP4MPT, the l-Cys adduct of PPP44mT (*cf.*Fig. 4D and F) and PPP4MT (*cf.*Fig. 5C and E) exhibited only partial conversion to the ligand upon incubation with H_2_O_2_. Interestingly, when comparing the redox activity of the Cu(ii) complexes of these ligands in cells (Fig. S10C), the difference observed extracellularly (Fig. S10A, B) was not apparent, as all the ligands showed similar redox activity in cells. This is likely because the intracellular ligand–Cu equilibrium is dominated by strong endogenous thiol-trapping and ROS detoxification systems,^86,87,102^ which probably suppresses the redox differences observed in extracellular assays.

Collectively, PPP4MPT emerged as the lead analogue of the PPP4HAT series, exhibiting potent anti-proliferative activity together with improved solubility, distinctive thiol reactivity, and pronounced Cu(ii)-mediated redox activity. However, the corresponding Fe(iii) complex also exhibited the highest oxy-Mb oxidation within the series, highlighting the importance of balancing favorable biological properties with off-target redox activity. Together, these findings provide new insight into the structure–activity relationships of this class of thiosemicarbazones and will guide the continued optimization and rational design of future analogues.

## Experimental section

The following general materials and methods are included in full in the SI, namely, chemicals, general methods, synthesis of novel PPP4HAT series, crystallographic studies, cyclic voltammetry, calculation of log *P* values, cell culture, the MTT cellular proliferation assay, l-Cys reactivity studies, kinetic studies, examination of ROS generation in solution using H_2_DCF-DA, intracellular H_2_DCF-DA studies, spectral analysis of met-Mb generation from oxy-Mb, molecular docking studies, western blot analysis, and statistics. The purity of ligands and their complexes was ≥ 95% as determined by elemental analysis (C, H, N, and S). Description of the electrochemistry of the Fe(iii) and Cu(ii) PPP4HAT complexes, DCF studies, the molecular docking studies of the Fe(iii) complexes, and the accompanying oxy-Mb oxidation profiles.

## Author contributions

D. R. R., B. K., and M. D. designed studies, supervised students and staff on the project, analyzed data, and obtained grant funding. T. P. W., B. K., M. D., and D. R. R. wrote the original text. T. P. W., B. K., and M. D. synthesized and characterized compounds, analyzed data, and conducted cellular or molecular studies. T. P. W, D. R. R., B. K., and M. D. edited the text, performed cellular or molecular experiments, ordered and obtained materials, and prepared figures. P. V. B. performed X-ray diffraction experiments, kinetic measurements, and data analysis, and contributed to writing and editing sections of the text. M. N. performed additional spectroscopic analysis, plotted NMR spectra, analyzed data, and edited sections.

## Conflicts of interest

The authors declare no competing financial interest.

## Abbreviations

Bp2mT: 2-Benzoylpyridine-2-methyl-3-thiosemicarbazone
COTI-2: *N*-(6,7-dihydro-5*H*-quinolin- 8-ylideneamino)-4-pyridin-2-ylpiperazine-1-carbothioamide
DCF: 2′,7′-dichlorofluorescein
DFO: desferrioxamine
DpC: di-2-pyridylketone 4-cyclohexyl-4-methyl-3-thiosemicarbazone
DpT: di-2-pyridylketone thiosemicarbazone
Dp44mT: di-2-pyridylketone-4,4-dimethyl-3-thiosemicarbazone
EGFR: epidermal growth factor receptor
ER-α: Estrogen receptor-α
GSH: glutathione
H_2_DCFDA: 2′,7′-dichlorodihydrofluorescein diacetate
Hb: hemoglobin
HBSS: Hank's balanced salt solution
HER2: human epidermal growth factor receptor 2
H_2_O_2_: hydrogen peroxide
IGF-1Rβ: insulin-like growth factor 1 receptor *β*
LC-MS: liquid chromatography-mass spectrometry
l-Cys: l-cysteine
Mb: myoglobin
met-Hb: met-hemoglobin
met-Mb: met-myoglobin
MIG6: mitogen-inducible gene 6
MTT: 3-(4,5-dimethylthiazol-2-yl)-2,5-diphenyltetrazolium bromide
NDRG1: *N*-myc downstream regulated gene-1
NHE: normal hydrogen electrode
NSCLC, non-small cell lung carcinoma
oxy-Hb: oxy-hemoglobin
oxy-Mb: oxy-myoglobin
Pgp: *P*-glycoprotein
PBS: phosphate-buffered saline
PPP4HAT: (*E*)-3-phenyl-1-(pyridine-2-yl)prop-2-en-1-one-heterocyclic amine thiosemicarbazone
PPP4MT: (*E*)-3-phenyl-1-(pyridine-2-yl)prop-2-en-1-one-morpholine-1-thiosemicarbazone
PPP4MPT: (*E*)-3-phenyl-1-(pyridine-2-yl)prop-2-en-1-one-methylpiperazine-1-thiosemicarbazone
PPP4pSe: (*E*)-3-phenyl-1-(2-pyridinyl)-2-propen-1-one-4-phenylselenosemicarbazone
PPPT: 3-phenyl-1-(2-pyridinyl)-2-propen-1-one-thiosemicarbazone
PPP4pT: (*E*)-3-phenyl-1-(2-pyridinyl)-2-propen-1-one-4-phenylthiosemicarbazone
PPP4PyrT: (*E*)-3-phenyl-1-(pyridine-2-yl)prop-2-en-1-one-pyrrolidine-1-thiosemicarbazone
PPP4TMT: (*E*)-3-phenyl-1-(pyridine-2-yl)prop-2-en-1-one-thiomorpholine-1-thiosemicarbazone
PPP4TzT: (*E*)-3-phenyl-1-(pyridine-2-yl)prop-2-en-1-one-thiazolidine-1-thiosemicarbazone
PPP44mT: (*E*)-3-phenyl-1-(2-pyridinyl)-2-propen-1-one 4,4-dimethyl-3-thiosemicarbazone
ROS: reactive oxygen species
SCLC: small-cell lung cancer
SPPP44mT: 3-phenyl-1-(2-pyridinyl)propan-1-one 4,4-dimethyl-3-thiosemicarbazone
TM: tetrathiomolybdate
TNBC: triple negative breast cancer
TfR1: transferrin receptor
Triapine: 3-aminopyridine-2-carboxaldehyde thiosemicarbazone
ROS: reactive oxygen species

## Supplementary Material

SC-017-D6SC02195F-s001

SC-017-D6SC02195F-s002

## Data Availability

CCDC 2451388–2451394 and 2478334 contain the supplementary crystallographic data for this paper.^103*a*–*h*^ All other raw data supporting the findings of this study are available from the corresponding authors upon reasonable request. Supplementary information (SI): all compounds reported in this study were verified to possess a purity greater than 95% by elemental analysis (C, H, N, S) and further characterized by ^1^H and ^13^C NMR spectroscopy, LC-MS, UV-vis spectrophotometry, and also single-crystal X-ray crystallography where applicable. Comprehensive experimental details are provided in the SI, including materials and general methods, crystallographic procedures and refinement data, electrochemical studies, calculated physicochemical parameters, synthetic procedures, and full characterization data for the ligands and their Fe(iii), Cu(ii), and Zn(ii) complexes. The SI also includes detailed biological and mechanistic experimental protocols, including cellular proliferation assays, cysteine and glutathione reactivity studies using UV-vis and LC-MS, kinetic analysis of cysteine reactions, extracellular and intracellular ROS generation assays using H_2_DCF-DA, spectral analysis of oxy-myoglobin oxidation, molecular docking studies, and western blot analyses. Supplementary tables and figures present crystallographic data, redox potentials, anti-proliferative activity, physicochemical parameters, docking results, cyclic voltammograms, UV-vis kinetics, ROS assays, and full NMR spectra of all compounds. See DOI: https://doi.org/10.1039/d6sc02195f.
