# Supplementary material for: Fine-tuning thiosemicarbazones with heterocyclic substituents: identification of styryl-dependent cysteine reactivity and potent anti-cancer activity
Source: Chem Sci. 2026 Jul 15. Online ahead of print. doi: 10.1039/d6sc02195f (PMC13430521; doi:10.1039/d6sc02195f)
Supplement: SC-OLF-D6SC02195F-s001 [file SC-OLF-D6SC02195F-s001.pdf]

Supplementary Information (SI) for Chemical Science.
This journal is © The Royal Society of Chemistry 2026

SUPPLEMENTARY INFORMATION

Fine-Tuning Thiosemicarbazones with Heterocyclic Substituents: Identification of Styryl-Dependent Cysteine Reactivity and Potent Anti-Cancer Activity

Tharushi P. Wijesinghe,[1] Mahendiran Dharmasivam,[1]* Busra Kaya,[1] Paul V. Bernhardt,[2] Masnun Naher,[2] and Des R. Richardson,[1,3]*

[1]*Centre for Cancer Cell Biology and Drug Discovery, Institute for Biomedicine and Glycomics, Griffith University, Parklands Drive, Gold Coast, Queensland, 4222, Australia*

[2]*School of Chemistry and Molecular Biosciences, University of Queensland, Brisbane, 4072, Australia*

[3]*Department of Pathology and Biological Responses, Nagoya University Graduate School of Medicine, Nagoya 466-8550, Japan.*

*__Corresponding authors__: **Dr. Des R. Richardson and Dr. Mahendiran Dharmasivam**, Centre for Cancer Cell Biology and Drug Discovery, Institute for Biomedicine and Glycomics, Griffith University, Parklands Drive, Gold Coast, Queensland, 4222, Australia. Email: d.richardson@griffith.edu.au and m.dharmasivam@griffith.edu.au

Table of Contents

Supplementary Procedures

Chemicals……………………..…………..…..…………………………………………….….……S5

General methods……………….……… …………………………………………………….……S5

Synthesis of novel PPP4HAT series….… ………………………………………………….……S5

Crystallographic studies………………...……………...……………………………...……S13

Electrochemistry…..………………………………..…………...……………….……..……S13

Calculation of log P values (log P_{calc})…………………………………………….…………S14

Cell culture ……...……………..……………………………………………………..…………S14

Cellular proliferation assay..……………………………..……………………….…..…S14

Examination of reactivity of ligands with L-Cys under physiological conditions using UV–Vis....S15

Examination of reactivity of ligands with L-Cys and GSH using LC-MS……..…......……..……S15

Kinetic studies with L-Cys ……………………….…………………………….….….…..S16

Examination of ROS generation using H_2DCF-DA ………………………..….…………....S16

Intracellular ROS measurement using H_2DCF-DA ……………………………..…..…………...S17

Spectral analysis of the oxidation of oxy-Mb to met-Mb………..…………………..………..…..S17

Molecular docking studies..………….………….…………..……..……………………….…..S18

Western blot analysis ..……….……………………………………………...…………….……S18

Statistics…...……..……………………….…………….………..………….…….…….…..S19

Supplementary Results and Discussion

Electrochemical modulation and fine-tuning of Fe(III) and Cu(II) complexes of the PPP4HAT analogues *via* terminal amine substitution……………………………………...……………S19

Generation of ROS by 1:1 Ligand: Cu(II) PPP4HAT complexes ………………….……. ……S22

Intracellular ROS generation assessed by DCF-DA…………… …………………………………S24

Qualitative Docking Analysis of Terminal Amine Substituted Fe(III) Complexes and Their Predicted Interactions with Oxy-Mb ……….…………...…………………………………………………S25

Supplementary Tables

Table S1: Crystal and refinement data table……….…………...…………………...…......…S27, S28

Table S2: Redox potentials of the Fe(III) and Cu(II) complexes of Dp44mT, DpC, PPP44mT, and PPP4pT relative to the PPP4HAT analogues………..…………………………………………….…S29

Table S3: Anti-proliferative activity of PPP4HAT analogues and their Fe(III) Cu(II) and Zn(II) complexes in DMS-53 and H1299 lung cancer cells ……………………………………...……..…S30

Table S4: Physicochemical properties of PPP4HAT analogues and their Fe(III) Cu(II) and Zn(II) complexes……………………………………………………………………………....….….…S31

Table S5: Binding energies and distances between Fe(III) complexes and the heme plane of oxy-Mb …………………………………………………………………………………...…...……S32

Supplementary Figures

Figure S1: Unit cell packing diagrams..…………….………...……………………………………..S33

Figure S2: Unit cell packing diagrams..…….……………...……………………………………..S34

Figure S3: *E/Z* isomerism and *syn*/*anti* conformations observed in PPP4HAT analogues. …...………………………………………………………………………………S34

Figure S4: Cyclic voltammograms of Fe(III) and Cu(II) complexes of Dp44mT, DpC, PPP44mT, and PPP4pT, shown relative to the Fe(III) and Cu(II) complexes of PPP4HAT analogues.…………...S35

Figure S5: Time-dependent spectra of PPP4MPT in the presence of L-Cys ….…………………...S36

Figure S6: Observed first-order rate constants for the reaction between PPP4MPT and L-Cys …...S36

Figure S7: UV spectra of PPP4MPT with L-Cys in LC-MS solvent system....……….……………S37

Figure S8: The desulfurization followed by intermolecular cyclization of PPP4MT and PPP4MPT ……………………………………………………………..………………………………S38

Figure S9: Reactivity of PPP44mT, SPPP44mT, PPP4MT and PPP4MPT with GSH …………...S39

Figure S10: Extracellular and intracellular ROS generation of 1:1 Cu:L complexes measured using DCF-DA assay…….……………………………………………………………. …………...S40

Figure S11: Correlation between anti-proliferative activity and DCF redox activity of the 1:1 Cu:L complexes……………………………………………………………………. …………...S41

Figure S12: Molecular docking illustrations of representative Fe(III) complexes with oxy-Mb showing key interaction types and distances ………………..…………………………..…...S42

Figures S13-S14: ^{1}H and ^{13}C NMR spectra of PPP4MT in $CDCl_3$....…………….……………...S43

Figures S15-S16: ^{1}H and ^{13}C NMR spectra of PPP4TMT in $CDCl_3$.....………..………….……....S44

Figures S17-S18: ^{1}H and ^{13}C NMR spectra of PPP4MPT in $CDCl_3$.....………..…...……………..S45

Figures S19-S20: ^{1}H and ^{13}C NMR spectra of PPP4PyrT in $CDCl_3$......………..……..……..S46

Figures S21-S22: ^{1}H and ^{13}C NMR spectra of PPP4TzT in $CDCl_3$.....………..……..………..S47

<u>Supplementary References</u>

References……………………...…………………………………………………...…..S48-52

<u>Materials and Methods</u>

Chemicals

Benzaldehyde, 2-acetylpyridine, *N*-methylaniline, carbon disulfide, sodium chloroacetate, morpholine, thiomorpholine, 1-methylpiperazine, pyrrolidine, thiazolidine, hydrazine monohydrate, NaOH, $Fe(ClO_4)_3 \cdot 6H_2O$, $CuCl_2 \cdot 2H_2O$, $Zn(ClO_4)_2 \cdot 6H_2O$, 3-(4, 5-dimethylthiazol-2-yl)-2,5-diphenyltetrazolium bromide (MTT), DFO, Triapine, COTI-2, HCl, horse skeletal muscle myoglobin (95–100% purity), and all solvents were of the highest analytical grade possible and purchased from Sigma-Aldrich (St. Louis, MO).

General methods

^1H and ^{13}C NMR spectra were recorded on a Bruker Avance III 400 NMR spectrometer (Billerica, MA) using $CDCl_3$-d_6. The chemical shift (δ) was referenced using the solvent peak as an internal standard. The elemental analysis (C, H, N, S) of the ligand and complexes was performed using a Thermo Scientific FlashSmart™ CHNS/O analyzer (Waltham, MA). Liquid chromatography mass spectrometry data were collected using a Thermo Fisher Orbitrap Exploris 120 Mass Spectrometer. Electronic absorption spectra were recorded between 200–800 nm using a Shimadzu UV-1800 spectrophotometer (Kyoto, Japan).

Synthesis of the Novel PPP4HAT Series

Synthesis and characterization of Dp44mT, DpC, PPP44mT, SPPP44mT, PPP4pT, $[Fe(Dp44mT)_2]^+$, $[Fe(DpC)_2]^+$, $[Fe(PPP44mT)_2]^+$, $[Fe(PPP4pT)_2]^+$, [Cu(Dp44mT)Cl], $[Cu(DpC)Cl_2]$, [Cu(PPP44mT)Cl], and [Cu(PPP4pT)Cl], were performed by our established procedures.[1-6]

General procedure for thiosemicarbazone preparation

3-phenyl-1-(pyridin-2-yl)prop-2-en-1-one chalcone (10 mmol) and heterocyclic amine–substituted thiosemicarbazides (15 mmol) were suspended in MeOH (30 mL), and 5 drops of concentrated HCl were added. The mixture was stirred for 24 h at room temperature. After this time, an orange precipitate was formed, which was filtered off and washed with cold EtOH and Et_2O.

(E)-3-phenyl-1-(2-pyridinyl)-2-propen-1-one-morpholine-4-thiosemicarbazone (PPP4MT).

Orange needles. (1.4 g). Yield: 85%. LC-MS (positive mode) in MeOH, found mass: 727.1 (100%), 375.1 (95%), 353.1 (30%), Calcd. mass for $C_{38}H_{40}N_8NaO_2S_2$: 727.26 $[2M + Na]^+$; $C_{19}H_{20}N_4NaOS$: 375.13 $[M + Na]^+$, $C_{19}H_{21}N_4OS$: 353.14 $[M + H]^+$. ^{1}H NMR δ ppm (400 MHz, $CDCl_3$ mixture of isomers): 14.53 (s, 0.75 H), 12.99 (s, 0.19 H), 8.80 – 8.76 (m, 1H), 8.13 (d, J = 16.3 Hz, 1H), 7.93 – 7.28 (m, 9H), 7.13 – 6.93 (m, 1H), 4.19 – 4.06 (m, 4H), 3.92 – 3.77 (m, 4H). ^{13}C NMR δ ppm (100 MHz, $CDCl_3$, mixture of isomers): 186.16, 181.48, 151.54, 150.63, 149.68, 148.84, 144.14, 143.64, 137.86, 137.44, 137.30, 136.37, 136.29, 135.70, 130.17, 129.16, 128.95, 128.89, 128.77, 127.85, 127.08, 126.04, 124.91, 124.51, 124.39, 122.06, 115.90, 77.48, 77.16, 76.84, 66.92, 66.70, 50.94. Anal. Calcd. (%) for $C_{19}H_{20}N_4OS$: C 64.75, H 5.72, N 15.90, S 9.10. Found: C 64.68, H 5.74, N 15.97, S 9.19. UV-Vis in DMSO [λ_{max} (nm) (ε, M^{-1} cm^{-1})]: 306 (25300), 459 (17400).

(E)-3-phenyl-1-(2-pyridinyl)-2-propen-1-one-thiomorpholine-4-thiosemicarbazone (PPP4TMT).

Dark orange crystals. (1.74 g). Yield: 83%. LC-MS (positive mode) in MeOH, found mass: 759.1 (100%), 391.2 (70%), 369.2 (40%), Calcd. mass for $C_{38}H_{40}N_8NaS_4$: 759.22 $[2M + Na]^+$; $C_{19}H_{20}N_4NaS_2$: 391.10 $[M + Na]^+$, $C_{19}H_{21}N_4S_2$: 369.12 $[M + H]^+$. ^{1}H NMR δ ppm (400 MHz, $CDCl_3$, mixture of isomers): 14.52 (s, 0.62 H), 13.05 (s, 0.15 H), 8.77 (dd, J = 11.1, 5.0 Hz, 1H), 8.19 (d, J = 16.3 Hz, 1H), 7.94 – 7.28 (m, 8H), 7.13 – 6.94 (m, 1H), 4.47 – 4.30 (m, 4H), 2.83 – 2.74 (m, 4H). ^{13}C NMR δ ppm (100 MHz, $CDCl_3$, mixture of isomers): 185.69, 181.29, 151.54, 150.61, 149.72, 148.78, 144.18, 143.73, 137.87, 137.45, 137.15, 136.32, 135.77, 130.18, 129.18, 128.89, 128.78, 127.84, 127.07, 126.01, 124.94, 124.48, 124.41, 121.94, 115.81, 77.48, 77.16, 76.84, 58.52, 53.64, 27.60, 27.49, 18.54. Anal. Calcd. (%) for $C_{19}H_{20}N_4S_2$: C 61.92, H 5.47, N 15.20, S 17.40. Found: C 61.85, H 5.51, N 15.24, S 17.43. UV-Vis in DMSO [λ_{max} (nm) (ε, M^{-1} cm^{-1})]: 307 (27200), 459 (19700).

(E)-3-phenyl-1-(2-pyridinyl)-2-propen-1-one-methylpiperazine-1-thiosemicarbazone (PPP4MPT). Dark yellow needles. (1.3 g). Yield: 62%. LC-MS (positive mode) in MeOH, found mass: 366.1 (100%), 388.1 (35%), 753.4 (15%), Calcd. mass for $C_{20}H_{24}N_5S$: 366.14 $[M + H]^+$, $C_{20}H_{23}N_5NaS$: 388.16 $[M + Na]^+$, $C_{40}H_{46}N_{10}NaS_2$: 753.32 $[2M + Na]^+$. ^1H NMR δ ppm (400 MHz, $CDCl_3$, mixture of isomers): 14.57 (s, 0.68H), 12.89 (s, 0.22 H), 8.80 (d, J = 4.9 Hz, 1H), 8.19 (d, J = 16.3 Hz, 1H), 7.94 – 7.73 (m, 2H), 7.67 – 7.56 (m, 2H), 7.52 – 7.28 (m, 5H), 7.16 – 6.91 (m, 1H), 4.15 (d, J = 74.8 Hz, 4H), 2.58 – 2.47 (m, 4H), 2.35– 2.34 (m, 3H). ^{13}C NMR *δ* ppm (100 MHz, $CDCl_3$, mixture of isomers): 185.97, 181.26, 150.67, 149.98, 148.93, 144.06, 143.46, 137.82, 137.43, 136.83, 136.51, 135.58, 130.11, 128.93, 128.74, 127.88, 127.11, 126.31, 124.92, 124.40, 121.92, 116.06, 77.41, 77.16, 76.91, 55.09, 54.93, 50.47, 45.97. Anal. Calcd. (%) for $C_{20}H_{23}N_5S$: C 65.72, H 6.17, N 19.16, S 8.77. Found: C 65.81, H 6.34, N 19.03, S 8.58. UV-Vis in DMSO [λ_{max} (nm) (ε, M^{-1} cm^{-1})]: 306 (46000), 458 (32500).

(E)-3-phenyl-1-(2-pyridinyl)-2-propen-1-one-pyrrolidine-1-thiosemicarbazone (PPP4PyrT). Yellow crystals. (1.1 g). Yield: 88%. LC-MS (positive mode) in MeOH, found mass: 695.2 (100%), 359.1 (20%), 337.1 (10%), Calcd. mass for $C_{38}H_{40}N_8NaS_2$: 695.27 $[2M + Na]^+$; $C_{19}H_{20}N_4NaS$: 359.13 $[M + Na]^+$, $C_{19}H_{21}N_4S$: 337.15 $[M + H]^+$. ^1H NMR δ ppm (400 MHz, $CDCl_3$, mixture of isomers): δ 14.64 (s, 0.24H) 13.43 (s, 0.61), 8.83 – 8.81 (m, 1H), 8.60 (d, J = 16.6 Hz, 1H), 8.09 – 7.29 (m, 9H), 7.03 (d, J = 7 Hz, 1H), 4.07 – 3.75 (m, 4H), 2.12 – 2.03 (m, 4H). ^{13}C NMR *δ* ppm (100 MHz, $CDCl_3$, mixture of isomers): 182.94, 177.63, 176.87, 151.91, 150.37, 150.14, 148.98, 148.38, 146.32, 144.27, 143.95, 140.67, 137.79, 137.40, 136.89, 136.63, 135.56, 135.08, 134.27, 129.77, 129.57, 129.05, 128.89, 128.78, 128.47, 127.71, 127.47, 127.02, 126.62, 125.03, 124.10, 123.94, 123.64, 121.99, 120.96, 116.13, 115.52, 77.48, 77.16, 76.84, 53.09, 51.53, 49.43, 48.32, 26.57, 25.72, 24.66, 24.29. Anal. Calcd. (%) for $C_{19}H_{20}N_4S$: C 67.88, H 5.95, N 16.65, S 9.53. Found: C 67.83, H 5.99, N 16.70, S 9.37. UV-Vis in DMSO [λ_{max} (nm) (ε, M^{-1} cm^{-1})]: 301 (25200), 356 (15600), 461 (10400).

(E)-3-phenyl-1-(2-pyridinyl)-2-propen-1-one-thiazolidine-3-thiosemicarbazone (PPP4TzT).

Thiazolidine-substituted thiosemicarbazide (6.7 mmol) and chalcone (4.5 mmol) were used for the preparation of PPP4TzT. Orange needles. (0.84 g). Yield: 89%. LC-MS (positive mode) in MeOH, found mass: 377.0 (100%), 731.3 (85%), 355.1 (25%), Calcd. mass for $C_{18}H_{18}N_4NaS_2$: 377.09 $[M + Na]^+$, $C_{36}H_{36}N_8NaS_4$: 731.18 $[2M + Na]^+$, $C_{18}H_{19}N_4S_2$: 355.10 $[M + H]^+$. ^{1}H NMR δ ppm (400 MHz, $CDCl_3$, mixture of isomers): 14.40 (s, 0.59 H), 13.55 (s, 0.29 H), 8.76 (t, J = 5.5 Hz, 1H), 8.34 (d, J = 16.1 Hz, 1H), 8.01 – 7.28 (m, 8H), 7.21 – 6.95 (m, 1H), 4.96 (s, 2H), 4.15 (t, J = 6.3 Hz, 2H), 3.13 (t, J = 6.2 Hz, 2H). ^{13}C NMR *δ* ppm (100 MHz, $CDCl_3$, mixture of isomers): 183.50, 177.67, 151.69, 150.53, 149.57, 148.50, 144.81, 144.42, 137.93, 137.50, 136.77, 136.42, 136.39, 135.72, 130.22, 129.15, 128.92, 128.85, 128.71, 127.88, 127.10, 125.98, 125.03, 124.50, 124.36, 121.68, 115.44, 77.48, 77.16, 76.84, 54.11, 50.31, 30.50. Anal. Calcd. (%) for $C_{18}H_{18}N_4S_2$: C 60.99, H 5.12, N 15.80, S 18.09. Found: C 60.70, H 5.24, N 15.82, S 18.19. UV-Vis in DMSO [λ_{max} (nm) (ε, M^{-1} cm^{-1})]: 304 (25700), 462 (15700).

General synthetic procedure of Fe(III) complexes from $Fe(ClO_4)_3.6H_2O$

The ligands and $Fe(ClO_4)_3.6H_2O$ (2:1 L:M ratio) were dissolved in 30 mL of EtOH. Then, Et_3N (2.1 mmol) was added, and the mixture was refluxed for 1 h. Upon cooling to room temperature, the resulting precipitate was filtered off, washed with cold EtOH and Et_2O, and dried *in vacuo*.

$[Fe(PPP4MT)_2](ClO_4)$

PPP4MT (0.50 g, 1.4 mmol), $Fe(ClO_4)_3.6H_2O$ (0.32 g, 0.7 mmol), and Et_3N (0.15 g, 1.47 mmol) were used as reagents for the preparation of $[Fe(PPP4MT)_2](ClO_4)$. Brown powder (0.42 g). Yield: 84%. LC-MS (positive mode) in MeOH, found mass: 758.2, Calcd. mass for $[C_{38}H_{38}N_8O_2S_2Fe]^+$: 758.19 $[M – ClO_4]^+$. Anal. Calcd. (%) for $[C_{38}H_{38}ClFeN_8O_6S_2]$: C 53.18, H 4.46, N 13.06, S 7.47. Found: C 53.07, H 4.30, N 13.19, S 7.23. UV−Vis in DMSO [λ_{max} (nm) (ε, M^{-1} cm^{-1})]: 314 (48400), 428 (29600), 520 (15400), 692 (7200).

[Fe(PPP4TMT)$_2$](ClO$_4$)

PPP4TMT (0.30 g, 0.8 mmol), Fe(ClO$_4$)$_3$.6H$_2$O (0.18 g, 0.4 mmol), and Et$_3$N (0.085 g, 0.84 mmol) were used as reagents for the preparation of [Fe(PPP4TMT)$_2$](ClO$_4$). Brown-black powder (0.25 g). Yield: 83%. LC-MS (positive mode) in MeOH, found mass: 790.1, Calcd. mass for $[C_{38}H_{38}N_8S_4Fe]^+$: 790.15 $[M - ClO_4]^+$. Anal. Calcd. (%) for [$C_{38}H_{38}ClFeN_8O_4S_4$]: C 51.26, H 4.30, N 12.59, S 14.41. Found: C 51.30, H 4.43, N 12.41, S 14.29. UV−Vis in DMSO [λ_{max} (nm) (ε, M^{-1} cm^{-1})]: 312 (46500), 424 (18000), 523 (11600), 694 (10500).

[Fe(PPP4MPT)$_2$](ClO$_4$)

PPP4MPT (0.3 g, 0.82 mmol), Fe(ClO$_4$)$_3$.6H$_2$O (0.19 g, 0.5 mmol) and Et$_3$N (0.091 g, 0.9 mmol) were used as reagents for the preparation of [Fe(PPP4MPT)$_2$](ClO$_4$). Brown-black powder (0.15 g). Yield: 50%. LC-MS (positive mode) in MeOH, found mass: 392.63 (100%), 784.2 (15%), Calcd. mass for $[C_{40}H_{44}N_{10}S_2Fe]^{++}$: 392.12 $[M - ClO_4]^{++}$, $[C_{40}H_{44}N_{10}S_2Fe]^+$: 784.25 $[M - ClO_4]^+$. Anal. Calcd. (%) for [$C_{40}H_{44}ClFeN_{10}O_4S_2$]: C 54.03, H 5.02, N 15.84, S 7.25. Found: C 54.33, H 4.87, N 15.56, S 6.93. UV−Vis in DMSO [λ_{max} (nm) (ε, M^{-1} cm^{-1})]: 315 (37100), 424 (18000), 520 (12900), 700 (1500).

[Fe(PPP4PyrT)$_2$](ClO$_4$)

PPP4PyrT (0.67 g, 2 mmol), Fe(ClO$_4$)$_3$.6H$_2$O (0.46 g, 1 mmol) and Et$_3$N (0.21 g, 2.1 mmol) were used as reagents for the preparation of [Fe(PPP4PyrT)$_2$](ClO$_4$). Brown-black powder (0.63 g). Yield: 94%. LC-MS (positive mode) in MeOH, found mass: 726.2, Calcd. mass for $[C_{38}H_{38}N_8S_2Fe]^+$: 726.20 $[M - ClO_4]^+$. Anal. Calcd. (%) for [$C_{38}H_{38}ClFeN_8O_4S_2$]: C 55.24, H 4.64, N 13.56, S 7.76. Found: C 55.37, H 4.38, N 13.53, S 7.92. UV−Vis in DMSO [λ_{max} (nm) (ε, M^{-1} cm^{-1})]: 314 (46300), 430 (23700), 520 (17500), 695 (2700).

[Fe(PPP4TzT)$_2$](ClO$_4$)

PPP4TzT (0.25 g, 0.7 mmol), $Fe(ClO_4)_3.6H_2O$ (0.16 g, 0.35 mmol) and Et_3N (0.074 g, 0.73 mmol) were used as reagents for the preparation of [Fe(PPP4TzT)$_2$](ClO_4). Brown-black powder (0.23 g). Yield: 92%. LC-MS (positive mode) in MeOH, found mass: 762.1, Calcd. mass for $[C_{36}H_{34}N_8S_4Fe]^+$: 762.11 $[M - ClO_4]^+$. Anal. Calcd. (%) for $[C_{36}H_{34}ClFeN_8O_4S_4]$: C 50.15, H 3.97, N 13.00, S 14.87 Found: C 50.36, H 3.68, N 13.14, S 14.95. UV−Vis in DMSO [λ_{max} (nm) (ε, M^{-1} cm^{-1})]: 313 (60900), 431 (29900), 509 (27100), 695 (2900).

General synthetic procedure of Cu(II) complexes from $CuCl_2.2H_2O$

The ligands were dissolved in 10 mL of DMF. Then, $CuCl_2·2H_2O$ was dissolved in 10 mL of water and added to the ligands (1:1 L:M ratio) with stirring for 1 h at room temperature. The brown precipitate formed was filtered off, washed with cold EtOH and Et_2O, and dried *in vacuo.*

[Cu(PPP4MT)Cl]

PPP4MT (0.35 g, 1 mmol) and $CuCl_2·2H_2O$ (0.17 g, 1 mmol) were used as reagents for the preparation of [Cu(PPP4MT)Cl]. Brown powder (0.43 g). Yield: 83%. LC-MS (positive mode) in MeOH, found mass: 414.0, Calcd. mass for $[C_{19}H_{19}N_4OSCu]^+$: 414.06 $[M - Cl]^+$. Anal. Calcd. (%) for $[C_{19}H_{19}ClCuN_4OS]$: C 50.66, H 4.25, N 12.44, S 7.12. Found: C 50.29, H 4.51, N 12.22, S 7.06. UV−Vis in DMSO [λ_{max} (nm) (ε, M^{-1} cm^{-1})]: 301 (39900), 462 (26000).

[Cu(PPP4TMT)Cl]

PPP4TMT (0.37 g, 1 mmol) and $CuCl_2·2H_2O$ (0.17 g, 1 mmol) were used as reagents for the preparation of [Cu(PPP4TMT)Cl]. Dark brown powder (0.37 g). Yield: 76%. LC-MS (positive mode) in MeOH, found mass: 430.1, Calcd. mass for $[C_{19}H_{19}N_4S_2Cu]^+$: 430.03 $[M - Cl]^+$. Anal. Calcd. (%) for $[C_{19}H_{19}ClCuN_4S_2]$: C 48.92, H 4.11, N 12.01, S 13.75. Found: C 48.81, H 4.39, N 12.27, S 13.78. UV−Vis in DMSO [λ_{max} (nm) (ε, M^{-1} cm^{-1})]: 300 (31900), 463 (21000).

[Cu(PPP4MPT)Cl]

2PPP4MPT (0.30 g, 0.82 mmol) and $CuCl_2·2H_2O$ (0.14 g, 0.82 mmol) were used as reagents for the preparation of [Cu(PPP4MPT)Cl]. Brown powder (0.21 g). Yield: 48%. LC-MS (positive mode) in MeOH, found mass: 427.08, Calcd. mass for $[C_{20}H_{22}N_5SCu]^+$: 427.09. $[M – Cl]^+$. Anal. Calcd. (%) for $[C_{20}H_{22}ClCuN_5S]$: C 51.83, H 4.78, N 15.11, S 6.92. Found: C 51.94, H 4.50, N 15.37, S 6.95. UV−Vis in DMSO [λ_{max} (nm) (ε, M^{-1} cm^{-1})]: 300 (30800), 458 (19300).

[Cu(PPP4PyrT)Cl]

PPP4PyrT (0.34 g, 1 mmol) and $CuCl_2·2H_2O$ (0.17 g, 1 mmol) were used as reagents for the preparation of [Cu(PPP4PyrT)Cl]. Brown powder (0.40 g). Yield: 78%. LC-MS (positive mode) in MeOH, found mass: 398.0, Calcd. mass for $[C_{19}H_{19}N_4SCu]^+$: 398.06 $[M – Cl]^+$. Anal. Calcd. (%) for $[C_{19}H_{19}ClCuN_4S]$: C 52.92, H 4.41, N 12.90, S 7.38. Found: C 52.53, H 4.14, N 12.73, S 7.05. UV−Vis in DMSO [λ_{max} (nm) (ε, M^{-1} cm^{-1})]: 301 (34800), 468 (22700).

[Cu(PPP4TzT)Cl]

PPP4TzT (0.25 g, 0.71 mmol) and $CuCl_2·2H_2O$ (0.12 g, 0.71 mmol) were used as reagents for the preparation of [Cu(PPP4TzT)Cl]. Brown powder (0.31 g). Yield: 84%. LC-MS (positive mode) in MeOH, found mass: 417.1, Calcd. mass for $[C_{18}H_{18}N_4S_2Cu]^+$: 417.03 $[M – Cl + H]^+$. Anal. Calcd. (%) for $[C_{18}H_{17}ClCuN_4S_2]$: C 47.78, H 3.79, N 12.38, S 14.17. Found: C 47.53, H 3.77, N 12.44, S 14.21. UV−Vis in DMSO [λ_{max} (nm) (ε, M^{-1} cm^{-1})]: 300 (33300), 464 (22100).

General synthetic procedure of Zn(II) complexes from $Zn(ClO_4)_2.6H_2O$

The ligands and $Zn(ClO_4)_2·6H_2O$ (2:1 L:M ratio) were dissolved in 30 mL of EtOH. Then, Et_3N (2.1 mmol) was added, and the mixture was refluxed for 2 h. Upon cooling to room temperature, a precipitate formed that was filtered off, washed with cold EtOH and Et_2O, and dried *in vacuo*.

[Zn(PPP4MT)$_2$](ClO$_4$)$_2$

PPP4MT (0.50 g, 1.4 mmol), Zn(ClO$_4$)$_2$·6H$_2$O (0.27 g, 0.7 mmol), and Et$_3$N (0.15 g, 1.47 mmol) were used as reagents for the preparation of [Zn(PPP4MT)$_2$] (ClO$_4$)$_2$. Yellow powder (0.46 g). Yield: 92%. LC-MS (positive mode) in MeOH, found mass: 767.1, Calcd. mass for [$C_{38}H_{39}N_8O_2S_2Zn$]: 767.19 [M - 2ClO$_4$+ H]$^+$. Anal. Calcd. (%) for [$C_{38}H_{38}Cl_2N_8O_{10}S_2Zn$]: C 47.19, H 3.96, N 11.59, S 6.63. Found: C 47.25, H 3.65, N 11.62, S 6.68. UV−Vis in DMSO [λ_{max} (nm) (ε, M^{-1} cm^{-1})]: 303 (32300), 433 (24700).

[Zn(PPP4TMT)$_2$](ClO$_4$)$_2$

PPP4TMT (0.3 g, 0.8 mmol), Zn(ClO$_4$)$_2$·6H$_2$O (0.15 g, 0.4 mmol), and Et$_3$N (0.085 g, 0.84 mmol) were used as reagents for the preparation of [Zn(PPP4TMT)$_2$](ClO$_4$)$_2$. Dark orange powder (0.22 g). Yield: 73%. LC-MS (positive mode) in MeOH, found mass: 799.15, Calcd. mass for [$C_{38}H_{39}N_8S_4Zn$]: 799.14 [M - 2ClO$_4$ + H]$^+$. Anal. Calcd. (%) for [$C_{38}H_{38}Cl_2N_8O_8S_4Zn$]: C 45.67, H 3.83, N 11.21, S 12.83. Found: C 45.91, H 3.58, N 11.60, S 12.92. UV−Vis in DMSO [λ_{max} (nm) (ε, M^{-1} cm^{-1})]: 306 (43900), 424 (18000).

[Zn(PPP4MPT)$_2$](ClO$_4$)$_2$

PPP4MPT (0.30 g, 0.82 mmol), Zn(ClO$_4$)$_2$·6H$_2$O (0.15 g, 0.41 mmol), and Et$_3$N (0.087 g, 0.86 mmol) were used as reagents for the preparation of [Zn(PPP4MPT)$_2$](ClO$_4$)$_2$. (0.2 g). Reddish powder. Yield: 67%. LC-MS (positive mode) in MeOH, found mass: 793.26, Calcd. mass for [$C_{40}H_{45}N_{10}S_2Zn$]: 793.25. [M - 2ClO$_4$ + H]$^+$. Anal. Calcd. (%) for [$C_{40}H_{44}Cl_2N_{10}O_8S_2Zn$]: C 48.37, H 4.47, N 14.10, S 6.46. Found: C 48.23, H 4.54, N 13.85, S 6.73. UV−Vis in DMSO [λ_{max} (nm) (ε, M^{-1} cm^{-1})]: 307 (38800), 436 (24500).

[Zn(PPP4PyrT)$_2$](ClO$_4$)$_2$

PPP4PyrT (0.50 g, 1.5 mmol), $Zn(ClO_4)_2 \cdot 6H_2O$ (0.28 g, 0.7 mmol), and Et_3N (0.16 g, 1.57 mmol) were used as reagents for the preparation of $[Zn(PPP4PyrT)_2](ClO_4)_2$. Dark yellow powder (0.57 g). Yield: 86%. LC-MS (positive mode) in MeOH, found mass: 735.10, Calcd. mass for $[C_{38}H_{39}N_8S_2Zn]$: 735.20 $[M - 2ClO_4 + H]^+$. Anal. Calcd. (%) for $[C_{38}H_{38}Cl_2N_8O_8S_2Zn]$: C 48.80, H 4.10, N 11.98, S 6.86. Found: C 48.64, H 4.36, N 11.69, S 6.74. UV−Vis in DMSO [λ_{max} (nm) (ε, M^{-1} cm^{-1})]: 302 (30100), 466 (17200).

$[Zn(PPP4TzT)_2](ClO_4)_2$

PPP4TzT (0.25 g, 0.71 mmol), $Zn(ClO_4)_2 \cdot 6H_2O$ (0.13 g, 0.35 mmol), and Et_3N (0.075 g, 0.75 mmol) were used as reagents for the preparation of $[Zn(PPP4TzT)_2](ClO_4)_2$. Reddish orange powder (0.21 g). Yield: 84%. LC-MS (positive mode) in MeOH, found mass: 771.12, Calcd. mass for $[C_{36}H_{35}N_8S_4Zn]$: 771.11 $[M - 2ClO_4 + H]^+$. Anal. Calcd. (%) for $[C_{36}H_{34}Cl_2N_8O_8S_4Zn]$: C 44.52, H 3.53, N 11.54, S 13.21. Found: C 44.75, H 3.81, N 11.36, S 13.57. UV−Vis in DMSO [λ_{max} (nm) (ε, M^{-1} cm^{-1})]: 305 (33900), 430 (25600).

Crystallographic studies

X-ray crystallographic studies were performed on either a Rigaku Synergy or Bruker D8 Venture diffractometer with Cu Kα radiation (1.54184 Å). Structures were solved with SHELXS[7] and refined with the SHELXL.[8] Thermal ellipsoid plots were generated with Mercury (CCDC). All crystallographic data in CIF format have been deposited with the CCDC (deposition numbers 2451388-2451394, 2478334).

Electrochemistry

Cyclic voltammograms of relevant Fe(III) and Cu(II) complexes were obtained using a Gamry Interface 1010B Potentiostat equipped with an aqueous Ag/AgCl reference electrode, a glassy carbon working electrode, and a Pt wire auxiliary electrode. All complexes were dissolved at 100 μM in

MeCN:H_2O (7:3 *v/v*) and used Bu_4NClO_4 (0.1 M) as the supporting electrolyte. Before measurements, the solutions of complexes were subjected to comprehensive N_2 purging.

Calculation of log P values (log P_{calc})

Logarithmic partition coefficients (log P_{calc}) and physicochemical parameters were calculated using the Molinspiration cheminformatics tool (http://www.molinspiration.com/). The reported values include log *P*, topological polar surface area (TPSA), hydrogen-bond acceptors (HBA), hydrogen-bond donors (HBD), and the number of rotatable bonds. All parameters were generated directly through this platform for consistent evaluation of Lipinski's parameters.

Biological Studies

Cell culture

All cell types were purchased from the American Type Culture Collection (ATCC; Manassas, VA). DMS-53 and H1299 lung cancer cells were grown in Roswell Park Memorial Institute 1640 media (RPMI 1640; Sigma-Aldrich). The MDA-MB-231 and MCF-7 breast cancer cells were grown in Minimum Essentials Medium Eagle media (MEM; Sigma-Aldrich). All media were supplemented with 10% fetal bovine serum, non-essential amino acids (1 mM), sodium pyruvate (1 mM), L-glutamine (2 mM), penicillin (100 U/mL), streptomycin (100 U/mL), and Fungizone (0.5 µg/mL). Cells were grown at 37°C under a 5% CO_2-humidified atmosphere in an incubator using well-established methodology.[9]

Cellular proliferation assay

Proliferation was assessed using the well-established MTT assay validated by viable cell counts using standard methods.[10] The ligands and complexes were dissolved in DMSO to prepare a stock solution of 10 mM, and then diluted in culture media with 10% FCS. For cell culture experiments, the

maximum DMSO concentration did not exceed 0.5% (*v/v*), and had no influence on proliferation.[10] Cells were seeded in 96-well plates (6,000-15,000 cells/well) and were incubated with serial dilutions of the agents for 24- or 72-h/37 °C. After these incubations, MTT (5 mg/mL in PBS) was added to the cells and incubated for 2 h/37 °C, and then the overlying media was carefully aspirated from the wells. The cells were subsequently solubilized by adding DMSO (100 µL), and after shaking the plates for 5 min, the absorbance was read using a CLARIOstar microplate reader (BMG LabTech, Germany) at a wavelength of 570 nm. MTT color formation was directly correlated with cell number.[10] Data analysis was performed using MARS Data Analysis Software (BMG LabTech; version 3.30). The concentration of agents required to inhibit proliferation by 50% (IC_{50}) was then calculated.

Examination of reactivity of ligands with L-Cys under physiological conditions using UV-Vis spectrophotometry

Solutions of the ligands, SPPP44mT, PPP4MT, and PPP4MPT, were prepared at 25 µM in either 20 mM HEPES buffer (0.14 M NaCl; pH 7.4) or 150 mM acetate buffer (0.14 M NaCl; pH 5.0) to mimic the cytosolic and lysosomal environment, respectively. To this solution, L-Cys (0 or 375 µM) was added and then incubated for 6 h/20ºC. The absorbance was measured using a Shimadzu UV−Vis spectrophotometer (UV-1800; Shimadzu, Kyoto, Japan), to obtain spectra between 200 and 800 nm.

Examination of the reactivity of ligands with L-Cys and GSH

The ligands, PPP44mT, SPPP44mT, PPP4MT, and PPP4MPT (500 µM) were reacted with L-Cys (5 mM), or glutathione (GSH; 5 mM) in a DMSO: H_2O (7:3 *v/v*) solution for 6 h/20 °C. The reaction mixtures were then diluted 1:1 with methanol, incubated for 10 min/20 °C, and prepared for liquid chromatography–mass spectrometry (LC–MS) analysis. LC-MS chromatographic separation was performed on a C18 column at 0.3 mL/min using a mobile phase containing 90% methanol, 9.9% water, and 0.1% formic acid. Mass spectroscopy measurements were performed in positive-ion mode

using a Thermo Fisher Orbitrap Exploris 120 mass spectrometer (Thermo Fisher Scientific, USA), equipped with an electrospray ionization (ESI) source. Thermo Fisher's proprietary software, Xcalibur and FreeStyle, were used for data acquisition and analysis, respectively, following established methods.[11, 12]

Kinetic studies of L-Cys

A stock solution of PPP4MPT (2 mM in DMSO) was diluted to 25 μM with either HEPES buffer (20 mM, pH 7.4, 0.14 M NaCl) or acetate buffer (20 mM, pH 5.0, 0.14 M NaCl). Then, 2 mL aliquots of this ligand solution were added to a spectrophotometer cuvette (1 cm pathlength), and each solution was thermostated at 37 °C in an Agilent 8453 spectrophotometer fitted with a Huber Ministat 125 temperature controller. To each cuvette, an aliquot of a freshly prepared 200 mM solution of L-Cys was added to give final L-Cys concentrations of 250 μM (2.5 μL), 500 μM (5 μL), and 1 mM (10 μL). Each cuvette was well mixed, and spectra were measured at 180 s intervals for 6 h/37 °C. Time-dependent spectra were examined by global analysis with Reactlab KINETICS.[13]

Examination of ROS generation using H_2DCF-DA

The production of ROS was evaluated by well-characterized procedures examining H_2DCF-DA oxidation.[2, 5, 14] Solutions of $CuCl_2$, tetrathiomolybdate (TM), the ligands (DpC, PPP44mT, PPP4pT, and the PPP4HAT series analogues), and their 1:1 Cu:L complexes were prepared at 5 μM in either HBSS (pH 7.4) or 150 mM acetate buffer (pH 5.0) to mimic the cytosolic and lysosomal environment, respectively. To this solution, L-cysteine (L-Cys; 100 μM) was added as a reducing agent, followed by H_2DCF-DA (5 μM). Then, hydrogen peroxide (H_2O_2; 100 μM) was added to initiate hydroxyl radical generation. The HBSS or acetate buffer with L-Cys, H_2DCF-DA, and H_2O_2, but without the addition of ligands or metal complexes, was implemented as a control. Fluorescence was analyzed by implementing a CLARIOstar Plus microplate reader (BMG LABTECH, Australia) at $\lambda_{excitation}$ = 485 nm and $\lambda_{emission}$ = 530 nm.

Intracellular ROS measurement using H_2DCF-DA

MCF-7 cells (6×10^5) were seeded in black 96-well plates and allowed to adhere for 24 h/37 °C. The medium was then replaced with fresh medium containing 10 μM of $CuCl_2$, tetrathiomolybdate (TM), DpC, [Cu(DpC)Cl_2], PPP44mT, [Cu(PPP44mT)Cl], PPP4pT, [Cu(PPP4pT)Cl], or PPP4HAT ligands and their respective Cu(II) complexes. After 4 h/37 °C incubation, 2′,7′-dichlorodihydrofluorescein diacetate (H_2DCF-DA; 5 μM, Sigma-Aldrich) was added, and cells were incubated for 45 min/37 °C in the dark. Fluorescence was measured using a CLARIOstar Plus microplate reader (BMG LABTECH, Australia) at $\lambda_{excitation}$ = 485 nm and $\lambda_{emission}$ = 530 nm.

Spectral analysis of the oxidation of oxy-Mb to met-Mb

Mb solutions were prepared using standard procedures.[15] Briefly, reduced Mb in PBS (pH 7.2) was prepared by adding 1.2 mL of $Na_2S_2O_4$ (12 mM) to 38 mL of met-Mb solution (40 μM). [15] The Mb was then passed through a PD-10 column (Pharmacia; prepacked 8.3 mL of Sephadex G-25) equilibrated with PBS. Oxy-Mb was then generated by gently bubbling 150 mL of O_2 (from a syringe filled with air) through the reduced Mb solution to remove residual $Na_2S_2O_4$.[15]

The effect of the Fe(III) complexes of PPP4HAT series analogues were compared to [Fe(DFO)], [Fe(Triapine)$_2$]$^+$, [Fe(Dp44mT)$_2$]$^+$, [Fe(DpC)$_2$]$^+$, [Fe(PPP44mT)$_2$]$^+$, and [Fe(PPP4pT)$_2$]$^+$. All ligands were dissolved in 1,2-propanediol and diluted to 10 μM in PBS (pH 7.4), and the experiment was performed over 0–3 h. Spectra (300−700 nm) were obtained at 20°C using a Shimadzu UV−Vis spectrophotometer (UV-1800; Shimadzu, Kyoto, Japan). Concentrations of met/reduced/oxy-Mb were determined at the following wavelengths, namely met-Mb at 409 nm; reduced-Mb at 435 nm; and oxy-Mb at 544 nm, respectively.[15]

Molecular docking studies

Molecular docking studies were carried out using AutoDock 4 and AutoDock Vina.[16, 17] The structure of $[Fe(Dp44mT)_2]^+$ and the Fe(III) complexes of all 5 PPP4HAT analogues were converted into PDB format from mol format by OPENBABEL.[18] The oxy-Mb crystal structure was downloaded from the Protein Data Bank (http://www.rcsb.org./pdb; PDB ID: 1MBO). Visualization of the docked position was done using UCSF Chimera[19] and Discovery Studio molecular graphics programs.[20]

Western blot analysis

DFO (Sigma-Aldrich Chemical Co., St. Louis) was dissolved in medium to a final concentration of 100 μM, while Bp2mT, Dp44mT, DpC, PPP44mT, PPP4pT, and the PPP4HAT analogues were examined at 5 μM and incubated with the cells for 24 h/37 °C. Total protein was extracted from cells, and western analysis was performed using established methods.[21] The primary antibodies used were against: (EGFR, 1:1000; Cat # 4267; Cell Signaling Technology, MA, USA), phosphorylated EGFR at Tyr1068 (p-$EGFR^{Tyr1068}$; 1:500; Cat. # 3777; Cell Signaling Technology), insulin-like growth factor 1 receptor β (IGF-1Rβ; 1:1000; Cat. # 9750; Cell Signaling Technology), MIG6 (1:500; Cat. # ab227944; Abcam Inc., Cambridge, UK), transferrin receptor 1 (TfR1; 1:1000; Cat. # 13-6800; Invitrogen, MA, USA); NDRG1 (1:1000; Cat. # ab37897; Abcam Inc.); and cyclin D1 (1:1000; Cat. # 2978; Cell Signaling Technology).

The Sapphire Biomolecular Imaging System (Azure Biosystems, Dublin, CA) was used for protein visualization. Densitometric analysis of blots was performed using ChemiDoc Image Lab software (Bio-Rad, Hercules, CA). All proteins were normalized to the β-actin loading control using an anti-β-actin antibody (1:5000; Cat. # A5316; Sigma-Aldrich).

Statistics

Experimental data were analyzed using Student's *t*-test. Results are expressed as mean ± standard deviation (SD) or mean ± standard error of the mean (SEM), as indicated in the corresponding figure legends and tables. Results were considered statistically significant when $p < 0.05$.

Supplementary Results and Discussion

Electrochemical Modulation and Fine-Tuning of Fe(III) and Cu(II) Complexes of the PPP4HAT Analogues via Terminal Amine Substitution

Fe(III) Complexes: Our previous studies involved examination of $[Fe(Dp44mT)_2]^+$, $[Fe(DpC)_2]^+$, $[Fe(PPP44mT)_2]^+$, and $[Fe(PPP4pT)_2]^+$.[2, 3, 22, 23] We expanded this framework of compounds by preparing Fe(III) complexes of the PPP4HAT ligands. In this new family, the N^4-phenyl moiety of PPP4pT was replaced with saturated heterocycles, morpholine, thiomorpholine, methyl piperazine, pyrrolidine, and thiazolidine, to modulate the redox potential of the $Fe^{(III)}/Fe^{(II)}$ couple through steric and electronic influences (**Table S2, Figure S4A**). Progressive ligand modification through the series, $[Fe(Dp44mT)_2]^+$, $[Fe(DpC)_2]^+$, $[Fe(PPP44mT)_2]^+$, and $[Fe(PPP4pT)_2]^+$, led to a progressive decrease in redox potential[2, 3, 22, 23] (**Table S2**). This effect mitigated off-target oxidation of oxy-Mb and oxy-Hb and also improved anti-proliferative efficacy.[3]

To quantify these redox changes, cyclic voltammetry was performed for all Fe(III) complexes in a MeCN:H_2O (7:3 *v/v*) solvent system, which was optimized and validated in-house to ensure uniform solubility across the analogues.[2] All Fe(III) complexes exhibited quasi-reversible one-electron redox events, with formal potentials calculated from the average of the anodic and cathodic peak potentials (**Figure S4A; Table S2**). The benchmark complexes $[Fe(Dp44mT)_2]^+$ and $[Fe(DpC)_2]^+$ displayed redox potentials of +166 mV and +149 mV *versus* the normal hydrogen electrode (NHE), respectively, consistent with their electron-withdrawing pyridine groups.[2] Replacing one pyridine donor in $[Fe(Dp44mT)_2]^+$ with the electron-donating styrene group in $[Fe(PPP44mT)_2]^+$, substantially lowered the potential to +82 mV (**Table S2**). Conversely, replacing the terminal methyl group in PPP44mT with a less electron-donating phenyl moiety in $[Fe(PPP4pT)_2]^+$ increased the redox potential to +114 mV (**Table S2**).[3]

We next investigated how variation in the terminal *N*-substituent influences $Fe^{(III)}/Fe^{(II)}$ redox behavior of the PPP4HAT series (**Main paper Figure 1H**). These ligands differ in donor atom identity (O, S, or N), ring size, and electron-donating character, allowing dissection of steric and inductive contributions to redox control.[24-26] All new complexes exhibited lower redox potentials than $[Fe(Dp44mT)_2]^+$ and $[Fe(DpC)_2]^+$ (**Table S2**). Of the novel PPP4HAT Fe(III) complexes, $[Fe(PPP4MT)_2]^+$ exhibited the highest potential (+140 mV *versus* the NHE), consistent with the moderate donor strength of the ether oxygen (**Main paper Figure 1H**).

Substitution of the oxygen atom with sulfur of thiomorpholine in $[Fe(PPP4TMT)_2]^+$ markedly reduced the potential to +81 mV, reflecting the stronger electron-donating nature of sulfur and its capacity to stabilize the Fe(III) oxidation state (**Table S2**). Examining the methyl-piperazine-substituted complex, $[Fe(PPP4MPT)_2]^+$ (+74 mV), the presence of two nitrogen electron donors, one *N*-methylated (**Main paper Figure 1H**), further enhanced electron density at the metal center, resulting in an additional decrease in the redox potential.[2, 3, 27, 28]

The pyrrolidine-substituted complex, $[Fe(PPP4PyrT)_2]^+$ (+60 mV; **Table S2**), displayed the lowest potential of the series. This can be attributed to the small, conformationally flexible five-membered pyrrolidine ring, which donates electron density strongly while minimizing steric hindrance.[2, 3] A comparable redox shift was observed in the thiazolidine-substituted complex, $[Fe(PPP4TzT)_2]^+$ (+69 mV; **Table S2**), which features both nitrogen and sulfur donors (**Main paper Figure 1H**). The slightly higher potential of $[Fe(PPP4TzT)_2]^+$ relative to $[Fe(PPP4PyrT)_2]^+$ (+60 mV), likely reflects subtle differences in donor geometry or bonding interactions.

Cu(II) Complexes: The benchmark complexes [Cu(Dp44mT)Cl], $[Cu(DpC)Cl_2]$, [Cu(PPP44mT)Cl] and [Cu(PPP4pT)Cl], together with the Cu(II) complexes of the new PPP4HAT series, were evaluated for their $Cu^{(II)}/Cu^{(I)}$ redox couples (**Table S2, Figure S4B**). All complexes exhibited quasi-reversible, one-electron reduction waves in MeCN: H_2O (7:3 *v/v*). Both [Cu(Dp44mT)Cl] and $[Cu(DpC)Cl_2]$

showed cathodic potentials of –160 mV and –141 mV, respectively, reflecting their electron-withdrawing pyridine moieties (**Table S2**). This was in good agreement with our prior studies.[2, 5, 23]

Replacement of pyridine by the electron-donating styrene moiety in [Cu(PPP44mT)Cl] shifted the potential markedly more negative (–250 mV), indicating diminished stabilization of Cu(II), as we demonstrated previously.[2] Incorporation of a phenyl group in [Cu(PPP4pT)Cl] relative to the methyl at the N-terminal of the ligand in [Cu(PPP44mT)Cl], resulted in less electron donation, moving the potential anodically to –208 mV *versus* the NHE.[3]

Across this PPP4HAT series, the ligands systematically tuned the Cu(II)/Cu(I) redox potential. Incorporation of morpholine in [Cu(PPP4MT)Cl] (–148 mV) and a methyl-piperazine substituent in [Cu(PPP4MPT)Cl] (–163 mV) shifted the potential anodically to more positive values relative to [Cu(PPP4pT)Cl] (–208 mV; **Table S2**). These results were consistent with enhanced electron donation by oxygen and tertiary nitrogen donors, respectively, in the morpholine and methyl piperazine analogues (**Main paper Figure 1H**). Replacing morpholine with thiomorpholine in [Cu(PPP4TMT)Cl] (–179 mV) induced a moderate cathodic shift, reflecting sulfur's stronger electron donor strength.[29, 30] The most cathodic potentials were observed for pyrrolidine ([Cu(PPP4PyrT)Cl], –188 mV) and thiazolidine ([Cu(PPP4TzT)Cl], –181 mV), which are correlated with their compact, highly electron-donating 5-member ring systems (**Table S2; Figure S4B**).

Compared to [Cu(Dp44mT)Cl] (–160 mV), the morpholine derivative [Cu(PPP4MT)Cl] (–148 mV) is anodically shifted by 12 mV, while the methyl-piperazine analogue [Cu(PPP4MPT)Cl] (–163 mV) remains essentially unchanged, and the thiomorpholine, thiazolidine, and pyrrolidine complexes shift cathodically by 19–28 mV. Relative to [Cu(DpC)Cl_2] (–141 mV), all PPP4HAT analogues exhibit cathodic shifts, ranging from 7 mV for morpholine to 47 mV for pyrrolidine (**Table S2; Figure S4B**).

Generation of ROS by 1:1 Ligand: Cu(II) PPP4HAT Complexes

To assess redox activity, a well-established fluorescence-based assay was used that measures oxidation of non-fluorescent, 2',7'-dichlorodihydrofluorescein-diacetate (H_2DCF-DA), to highly fluorescent, 2',7'-dichlorofluorescein (DCF).[3, 5, 31] To simulate intracellular environments, the redox activity of the 1:1 Cu:L complexes was evaluated under cytosolic (pH 7.4; HBSS buffer) and lysosomal (pH 5.0; acetate buffer) conditions (**Figure S10A, B**).[3, 12]

All agents (5 μM) were added to these buffers, followed by a reducing agent, L-cysteine (100 μM). Subsequently, non-oxidized H_2DCF-DA (5 μM) was added, followed by the final addition of H_2O_2 (100 μM) to trigger redox activity. As positive controls, 1:1 Cu:L complexes of the previous generation of thiosemicarbazones, [Cu(DpC)Cl_2], [Cu(PPP44mT)Cl], and [Cu(PPP4pT)Cl] were used, considering their known ability to induce redox activity.[2, 3] Conversely, the ligands of these complexes, DpC, PPP44mT, and PPP4pT, were utilized as negative controls, considering their inherent lack of redox activity.[2, 3] As an additional control, the Cu chelator, tetrathiomolybdate (TM), was included to remove Cu from the Cu(II) complexes and assess its contribution to redox activity.[2, 3, 12]

At both pH 7.4 and pH 5.0, TM and the ligands, including DpC, PPP44mT, and PPp4pT, along with the PPP4HAT analogues, demonstrated negligible levels of fluorescence, indicating that they are redox inactive by themselves (**Figure S10A, B**). A significant ($p < 0.001$) increase in fluorescence was observed with $CuCl_2$ under both cytosolic and lysosomal conditions relative to the control, while the addition of TM substantially suppressed this effect (**Figure S8A, B**). All 1:1 Cu:L complexes led to a significant increase in fluorescence under both conditions, while [Cu(PPP4TzT)Cl] was the only exception, as it did not induce a marked level of fluorescence under cytosolic conditions (**Figure S10A**).

At pH 7.4, [Cu(DpC)Cl_2] induced the highest level of DCF fluorescence amongst all the Cu(II) complexes. Of the novel PPP4HAT series, the 1:1 Cu:L complex of the methyl-piperazine analogue, [Cu(PPP4MPT)Cl], demonstrated the greatest DCF fluorescence, which was significantly ($p < 0.001$) greater than fluorescence induced by the other 1:1 Cu:L complexes (**Figure S10A**). The Cu(II) complexes, [Cu(PPP4MT)Cl] and [Cu(PPP4PyrT)Cl], demonstrated a slight, but significant ($p <0.05$) increase in fluorescence over the 1:1 Cu:L complexes of thiomorpholine and thiazolidine analogues (**Figure S10A**).

Surprisingly, under lysosomal pH, the highest level of fluorescence was induced by [Cu(PPP4MPT)Cl], which was significantly ($p < 0.001$) greater than [Cu(DpC)Cl_2] (**Figure S10B**), which has consistently produced the highest fluorescence in previous studies.[2, 3, 12] Fluorescence intensity shown by the 1:1 Cu:L complex of the morpholine analogue, [Cu(PPP4MT)Cl], was similar to [Cu(DpC)Cl_2] and significantly greater compared to the rest of the 1:1 Cu(II) complexes of the novel heterocyclic amine-derived thiosemicarbazone series (**Figure S10B**).

After plotting the redox activity after a 24 h/37 °C incubation of these 1:1 Cu(II) complexes against their anti-proliferative activity in breast cancer cells (**Table 1**) and lung cancer cells (**Table S3**), positive correlations of $r = 0.65$ (pH 5.0; **Figure S11A**) and $r = 0.61$ (pH 7.4; **Figure S11B**) were observed. However, after a 72 h/37 °C incubation, only a weak negative correlation was observed at pH 5.0 (**Figure S11C**) and pH 7.4 (**Figure S11D**). This observation may be because most of the Cu(II) complexes exhibited much greater and more comparable anti-proliferative activity after 72 h *versus* 24 h.

Intracellular ROS Generation Assessed by DCF-DA

The intracellular ROS levels induced by the PPP4HAT ligands and their Cu(II) complexes (10 μM) were quantified relative to the control and the Cu chelator, TM (10 μM), using MCF-7 breast cancer cells incubated for 4 h/37 °C followed by a 45 min/37 °C incubation with H_2DCF-DA (5 μM; **Figure**

S10C). This latter cell type was used as all the Cu(II) complexes exhibited marked anti-proliferative activity upon their incubation (**Table 1**).

All Cu(II) complexes and $CuCl_2$ produced a slight and significant (p < 0.0001–0.001) increase in intracellular DCF fluorescence relative to the untreated control, consistent with their redox-active nature.[2, 3, 32, 33] Co-treatment with TM markedly suppressed this increase in fluorescence across $CuCl_2$ and all Cu(II) complexes, confirming that it is predominantly Cu-dependent.[2, 3, 32, 33]

However, in contrast to the extracellular DCF assay at pH 7.4 and pH 5.0 (**Figure S10A, B**), studies using the cellular DCF assay demonstrated only slight differences in redox activity among the individual Cu(II) complexes (**Figure S10C**). Notably, treatment of the control with TM alone also significantly (p < 0.0001) altered cellular ROS levels and confirmed TM's ability to bind intracellular Cu pools.[34, 35] The relatively low intracellular ROS levels induced by all Cu(II) complexes could be explained by the batteries of cellular antioxidant enzymes and GSH, which probably act to decrease intracellular ROS levels.[36, 37]

Unlike the extracellular redox activity of the Cu(II) complexes (**Figure S11A, B**), there was little correlation between cellular redox activity plotted against the IC_{50} after a 24-h incubation (r = 0.35; data not shown). Similarly, after a 72-h incubation, poor correlation (r = 0.23; data not shown) was also demonstrated. These latter data suggest the relationship between anti-proliferative activity and ROS generation is complex, with other factors also probably playing a role, such as the ability of thiosemicarbazones to modulate proliferation-related signaling pathways.[38-40]

Qualitative Docking Analysis of Terminal Amine Substituted Fe(III) Complexes and Their Predicted Interactions with Oxy-Mb

To gain further qualitative structural insights into the predicted orientation and accessibility of the Fe(III) complexes relative to the oxy-Mb heme pocket, molecular docking studies were performed

for the new PPP4HAT series. These analogues were evaluated alongside the previously reported complexes, $[Fe(Dp44mT)_2]^+$, $[Fe(PPP44mT)_2]^+$, and $[Fe(PPP4pT)_2]^+$, to assess how variations at the amine terminus influence their predicted orientation and accessibility to oxy-Mb heme pocket (**Table S5; Figure S12**).[2, 3] Docking results from both AutoDock 4 and AutoDock Vina demonstrated that $[Fe(Dp44mT)_2]^+$, bearing a compact dimethylamino group, exhibits the lowest binding energy (+3.32 to +6.60 kcal/mol) and the closest interaction with the heme plane (2.51–2.75 Å). The predicted orientation includes π-anion electrostatic interactions, and a C–H···O hydrogen bond near the heme propionate group (**Figure S12A**).[2, 3] In contrast, $[Fe(PPP44mT)_2]^+$, which retains the same dimethylamino group but substitutes the pyridyl core with a bulkier styrene moiety, exhibits a markedly weaker interaction (+17.8 to +24.5 kcal/mol). As such, $[Fe(PPP44mT)_2]^+$ is located farther from the heme surface (5.78–6.12 Å; **Table S5**), qualitatively consistent with its lower experimentally observed oxy-Mb oxidation.[2]

Replacing the dimethylamino group in $[Fe(PPP44mT)_2]^+$ with a phenyl ring in $[Fe(PPP4pT)_2]^+$ further reduced oxy-Mb oxidation.[3] This shift is reflected in both increased binding energies (+28.43–34.4 kcal/mol) and greater distances from the heme plane (7.01–7.30 Å; **Table S5**). These docking results suggest that the planar, electron-withdrawing phenyl group introduces significant steric and electronic hindrance, preventing interaction with the heme plane.[3]

To dissect the impact of non-aromatic, flexible, and heterocyclic amine substituents, we next investigated the five novel PPP4HAT analogues. Notably, the methyl-piperazine complex $[Fe(PPP4MPT)_2]^+$ showed considerable interactions, including a carbon–hydrogen bond (2.89 Å) near the heme surface and a short distance (2.89–2.99 Å) comparable to $[Fe(Dp44mT)_2]^+$ (**Figure S12B; Table S5**). These structural features qualitatively consistent with the experimentally observed oxy-Mb oxidation profile of $[Fe(PPP4MPT)_2]^+$.

In contrast, complexes containing morpholine ($[Fe(PPP4MT)_2]^+$) and thiazolidine ($[Fe(PPP4TzT)_2]^+$) engaged in π–alkyl hydrophobic interactions at 5.26 Å and 5.31 Å, respectively (**Figure S12C, D**), which, although at intermediate distances from the heme plane, reflect limited approach to the heme edge. These predicted orientations are qualitatively consistent with the experimentally observed oxy-Mb oxidation trends (**Figure 6A**).

Lastly, thiomorpholine and pyrrolidine complexes exhibited distinct behaviors (**Table S5**). The pyrrolidine-based $[Fe(PPP4PyrT)_2]^+$, with its compact, electron-rich ring, approached within ~5.09–5.37 Å of heme, exhibiting moderate binding energies and oxidation activity. In contrast, $[Fe(PPP4TzT)_2]^+$, bearing a bulkier fused thiazolidine–thiophene moiety, showed higher docking scores (+21.14–25.9 kcal/mol) and a larger separation (4.99–5.31 Å) from heme. As such, these docking simulations suggest reduced accessibility of the complex to the heme environment (**Figure S12D; Table S5**).

Table S1. Crystal and refinement data for the PPP4HAT series of ligands evaluated.

	**PPP4MT**	**PPP4TMT**	**PPP4MPT**	**PPP4PyrT**	**PPP4TzT**
Empirical formula	$C_{19}H_{20}N_4OS$	$C_{19}H_{20}N_4S_2$	$C_{20}H_{23}N_5S$	$C_{19}H_{20}N_4S$	$C_{18}H_{18}N_4S_2$
M.W	352.45	368.51	365.49	336.45	354.48
Crystal system	Monoclinic	Monoclinic	Monoclinic	Monoclinic	Monoclinic
Space group	$C2/c$	$C2/c$	$P2_1/c$	$P2_1/c$	$C2/c$
a (Å)	31.661(2)	25.631(1)	11.3196(8)	10.3403(9)	30.246(3)
b (Å)	5.5950(4)	6.3474(3)	19.4334(13)	10.2598(8)	5.5473(5)
c (Å)	24.693(2)	23.052(1)	8.6478(6)	16.0187(12)	24.428(2)
α (°)	90	90	90	90	90
β (°)	125.549(2)	103.139(4)	99.746(3)	90.740(3)	123.433(3)
γ (°)	90	90	90	90	90
V (Å^3)	3559.0(4)	3652.1(3)	1874.9(2)	1699.3(2)	3420.3(6)
T (K)	150(2)	150(2)	150(2)	150(2)	150(2)
Z	8	8	4	4	8
R_1 (obs. data)	0.0514	0.0380	0.0299	0.0442	0.0528
wR_2 (all data)	0.1681	0.1035	0.0759	0.1365	0.1636
GOF	1.039	1.042	1.064	1.044	0.990
CCDC No.	2451388	2451389	2451390	2451391	2451392

Table S1 (continued). Crystal and refinement data for the Fe(III) complex of PPP4TzT and Cu(II) complexes of PPP4PyrT and PPP4MT.

	**[Fe(PPP4TzT)$_2$](ClO$_4$)**	**[Cu(PPP4PyrT)Cl]**	**[Cu(PPP4MT)Cl]**
Empirical formula	$C_{36}H_{34}ClFeN_8O_4S_4$	$C_{19}H_{19}ClCuN_4S$	$C_{19}H_{19}ClCuN_4OS$
M.W	862.25	434.43	450.43
Crystal system	Triclinic	Monoclinic	Monoclinic
Space group	$P\bar{1}$	$P2_1/c$	$P2_1/n$
a (Å)	8.4454(2)	13.6433(5)	7.5085(3)
b (Å)	14.4395(4)	18.5544(7)	24.0808(9)
c (Å)	16.9690(5)	14.9852(5)	21.4062(9)
α (°)	80.095(2)	90	90
β (°)	82.840(1)	98.705(2)	91.130(1)
γ (°)	82.971(1)	90	90
V (Å^3)	2011.8(1)	3749.7(2)	3869.7(3)
T (K)	100(2)	150(2)	150(2)
Z	2	8	8
R_1 (obs. data)	0.0650	0.0222	0.0272
wR_2 (all data)	0.1920	0.0625	0.0700
GOF	1.074	1.017	1.020
CCDC No.	2451393	2451394	2478334

Table S2. Redox potentials of the Fe(III) and Cu(II) complexes of Dp44mT, DpC, PPP44mT, and PPP4pT relative to the PPP4HAT analogues (100 µM), as determined by cyclic voltammetry in MeCN/H_2O 7:3 (*v/v*) + 0.1 M Bu_4NClO_4 at a concentration of 100 μM. All potentials were calculated *versus* the NHE.

Complexes	Redox potential (mV *versus* NHE)
$[Fe(Dp44mT)_2]^+$	+166
$[Fe(DpC)_2]^+$	+149
$[Fe(PPP44mT)_2]^+$	+82
$[Fe(PPP4pT)_2]^+$	+114
$[Fe(PPP4MT)_2]^+$	+140
$[Fe(PPP4TMT)_2]^+$	+81
$[Fe(PPP4MPT)_2]^+$	+74
$[Fe(PPP4PyrT)_2]^+$	+60
$[Fe(PPP4TzT)_2]^+$	+69
[Cu(Dp44mT)Cl]	–160
$[Cu(DpC)Cl_2]$	–141
[Cu(PPP44mT)Cl]	–250
[Cu(PPP4pT)Cl]	–208
[Cu(PPP4MT)Cl]	–148
[Cu(PPP4TMT)Cl]	–179
[Cu(PPP4MPT)Cl]	–163
[Cu(PPP4PyrT)Cl]	–188
[Cu(PPP4TzT)Cl]	–181

Table S3. Inhibition of the proliferation (IC_{50}; μM) of DMS-53 SCLC and H1299 NSCLC cells by DFO, Triapine, Dp44mT, DpC, PPP44mT, and PPP4pT relative to the PPP4HAT series of ligands and their Fe(III), Cu(II), and Zn(II) complexes. Proliferation was examined using the MTT assay after a 24- or 72-h incubation at 37°C. Results are Mean ± SD (3 experiments).

Compounds	IC_{50} (μM)			
	DMS-53		H1299	
	24 h	72 h	24h	72h
DFO	>100	12.093 ± 0.798	>100	10.738 ± 0.586
Triapine	>25	1.228 ± 0.057	>25	0.024 ± 0.010
Dp44mT	2.667 ± 0.022	0.005 ± 0.001	1.928 ± 0.543	0.005 ± 0.001
DpC	11.184 ± 1.071	0.005 ± 0.001	19.229 ± 0.297	0.005 ± 0.001
PPP44mT	9.372 ± 0.857	0.006 ± 0.001	8.280 ± 0.132	0.005 ± 0.001
PPP4pT	15.283 ± 0.934	0.093 ± 0.012	1.019 ± 0.326	0.031 ± 0.007
PPP4MT	17.121 ± 0.457	0.006 ± 0.001	7.603 ± 0.348	0.005 ± 0.001
$[Fe(PPP4MT)_2]^+$	0.604 ± 0.029	0.205 ± 0.010	0.266 ± 0.019	0.242 ± 0.008
[Cu(PPP4MT)Cl]	0.402 ± 0.016	0.006 ± 0.001	0.642 ± 0.056	0.005 ± 0.001
$[Zn(PPP4MT)_2]$	>25	0.006 ± 0.001	>25	0.005 ± 0.001
PPP4TMT	9.073 ± 0.123	0.115 ± 0.019	7.206 ± 0.396	0.526 ± 0.077
$[Fe(PPP4TMT)_2]^+$	9.878 ± 1.235	0.373 ± 0.008	0.877 ± 0.166	0.226 ± 0.007
[Cu(PPP4TMT)Cl]	0.238 ± 0.014	0.075 ± 0.002	0.317 ± 0.026	0.013 ± 0.002
$[Zn(PPP4TMT)_2]$	>25	0.281 ± 0.016	>25	0.125 ± 0.005
PPP4MPT	4.803 ± 0.650	0.005 ± 0.001	0.647 ± 0.075	0.005 ± 0.001
$[Fe(PPP4MPT)_2]^+$	1.054 ± 0.204	0.405 ± 0.013	0.655 ± 0.020	0.469 ± 0.007
[Cu(PPP4MPT)Cl]	0.580 ± 0.023	0.005 ± 0.001	0.709 ± 0.017	0.005 ± 0.001
$[Zn(PPP4MPT)_2]$	5.308 ± 0.672	0.005 ± 0.001	3.225 ± 0.069	0.005 ± 0.001
PPP4PyrT	14.026 ± 1.540	0.006 ± 0.001	16.730 ± 0.515	0.005 ± 0.001
$[Fe(PPP4PyrT)_2]^+$	1.391 ± 0.078	0.261 ± 0.005	0.304 ± 0.022	0.122 ± 0.002
[Cu(PPP4PyrT)Cl]	0.279 ± 0.016	0.007 ± 0.001	0.338 ± 0.001	0.005 ± 0.001
$[Zn(PPP4PyrT)_2]$	>25	0.015 ± 0.003	>25	0.007 ± 0.001
PPP4TzT	8.994 ± 1.207	0.047 ± 0.003	1.080 ± 0.256	0.006 ± 0.001
$[Fe(PPP4TzT)_2]^+$	7.525 ± 1.572	0.218 ± 0.006	0.359 ± 0.009	0.225 ± 0.012
[Cu(PPP4TzT)Cl]	0.225 ± 0.022	0.027 ± 0.002	0.393 ± 0.011	0.005 ± 0.001
$[Zn(PPP4TzT)_2]$	>25	0.153 ± 0.014	>25	0.034 ± 0.004

Table S4. Physicochemical properties of Dp44mT, DpC, PPP44mT, and PPP4pT, relative to the five PPP4HAT analogues (PPP4MT, PPP4TMT, PPP4MPT, PPP4PyrT, and PPP4TzT) and their Fe(III) Cu(II) and Zn(II) complexes. Calculated using cheminformatics (http://www.molinspiration.com/).[2]

**Compounds**	**MW (KDa)**	**log *P* (calc.)**	**HBA (N + O)**	**HBD (NH + OH)**	**Rot. bonds**	**TPSA (Å²)**
Dp44mT	285.37	1.29	5	1	5	53.41
DpC	353.48	3.20	5	1	6	53.41
PPP44mT	310.42	3.22	4	1	6	40.52
PPP4pT	358.46	4.04	4	2	7	49.31
PPP4MT	352.46	3.07	5	1	6	49.76
$[Fe(PPP4MT)_2]^+$	758.75	6.17	10	0	6	63.45
[Cu(PPP4MT)Cl]	450.45	3.33	5	0	3	31.73
$[Zn(PPP4MT)_2]$	768.30	7.18	10	0	6	63.45
PPP4TMT	368.53	3.61	4	1	6	40.52
$[Fe(PPP4TMT)_2]^+$	790.89	7.26	8	0	6	44.98
[Cu(PPP4TMT)Cl]	466.52	3.88	4	0	3	22.49
$[Zn(PPP4TMT)_2]$	800.44	8.23	8	0	6	44.98
PPP4MPT	365.51	3.11	5	1	6	43.76
$[Fe(PPP4MPT)_2]^+$	784.84	6.27	10	0	6	51.46
[Cu(PPP4MPT)Cl]	463.50	3.38	5	0	3	25.73
$[Zn(PPP4MPT)_2]$	794.39	7.27	10	0	6	51.46
PPP4PyrT	336.46	3.62	4	1	6	40.52
$[Fe(PPP4PyrT)_2]^+$	726.76	7.29	8	0	6	44.98
[Cu(PPP4PyrT)Cl]	434.45	3.89	4	0	3	22.49
$[Zn(PPP4PyrT)_2]$	736.30	8.25	8	0	6	44.98
PPP4TzT	354.50	3.59	4	1	6	40.52
$[Fe(PPP4TzT)_2]^+$	762.84	7.23	8	0	6	44.98
[Cu(PPP4TzT)Cl]	452.50	3.86	4	0	3	22.49
$[Zn(PPP4TzT)_2]$	772.38	8.21	8	0	6	44.98
**Required**	**< 500**	**< 5**	**< 10**	**< 5**	**< 10**	**< 140**

Required parameters to fulfill physicochemical properties important for bioavailability as judged appropriate according to Lipinski's Rules: molecular weight (MW) ≤ 500 KDa, partition coefficient (log P_{calc}) ≤ 5, number of hydrogen bond acceptors (HBA) ≤10, number of hydrogen bond donors (HBD) ≤5, number of rotatable bonds ≤10, and topological polar surface area (TPSA) ≤140 Å.[41-43]

Table S5. Binding energies and distances between Fe(III) complexes and the heme plane of oxy-Mb as calculated by AutoDock 4 and AutoDock Vina. Docking simulations were performed to evaluate the interaction of Fe(III) complexes bearing various right-terminal amine substituents, including dimethylamino, morpholine, thiomorpholine, methyl-piperazine, pyrrolidine, and thiazolidine. Binding energies (in kcal/mol) and the distances (in Å) between the complex and the heme plane are shown. Lower binding energies and shorter distances reflect closer and potentially more favorable interactions with oxy-Mb, leading to its oxidation.

**Compounds**	**Binding energy (kcal/mol)**		**Distances between complexes and heme plane (Å)**	
	AutoDock 4	AutoDock Vina	AutoDock 4	AutoDock Vina
$[Fe(Dp44mT)_2]^+$	+3.32	+6.60	2.75	2.51
$[Fe(DpC)_2]^+$	+10.79	+12.0	2.84	3.18
$[Fe(PPP44mT)_2]^+$	+17.8	+24.5	5.78	6.12
$[Fe(PPP4pT)_2]^+$	+28.43	+34.4	7.01	7.30
$[Fe(PPP4MT)_2]^+$	+23.01	+27.31	5.26	5.43
$[Fe(PPP4TMT)_2]^+$	+26.12	+31.13	5.96	5.68
$[Fe(PPP4MPT)_2]^+$	+26.91	+30.87	2.89	2.99
$[Fe(PPP4PyrT)_2]^+$	+23.08	+29.4	5.09	5.37
$[Fe(PPP4TzT)_2]^+$	+21.14	+25.9	5.31	4.99

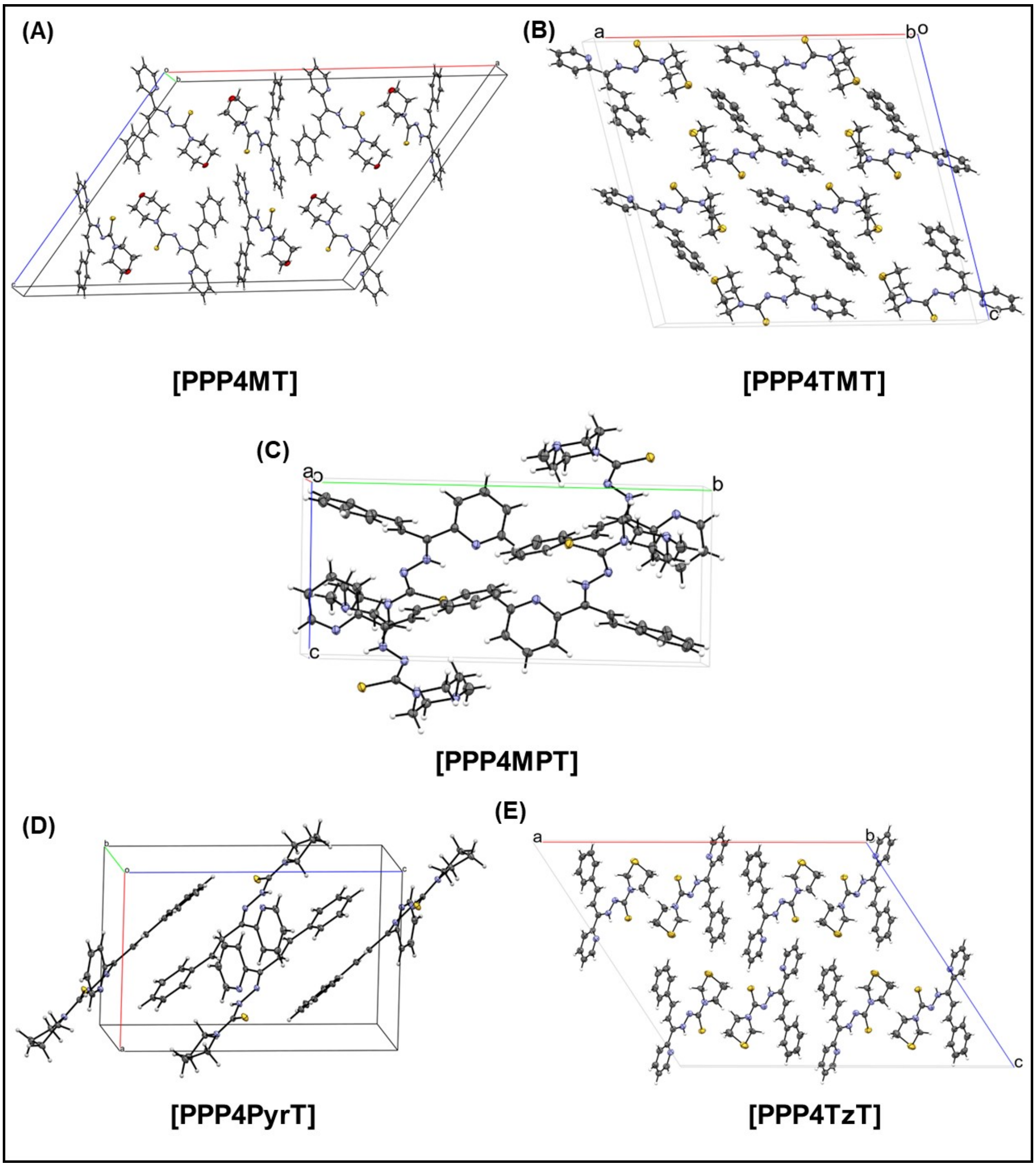

Figure S1. Unit cell packing diagrams) for the ligands, namely: **(A)** PPP4MT; **(B)** PPP4TMT; **(C)** PPP4MPT; **(D)** PPP4PyrT; and **(E)** PPP4TzT. The H-atoms are omitted for clarity.

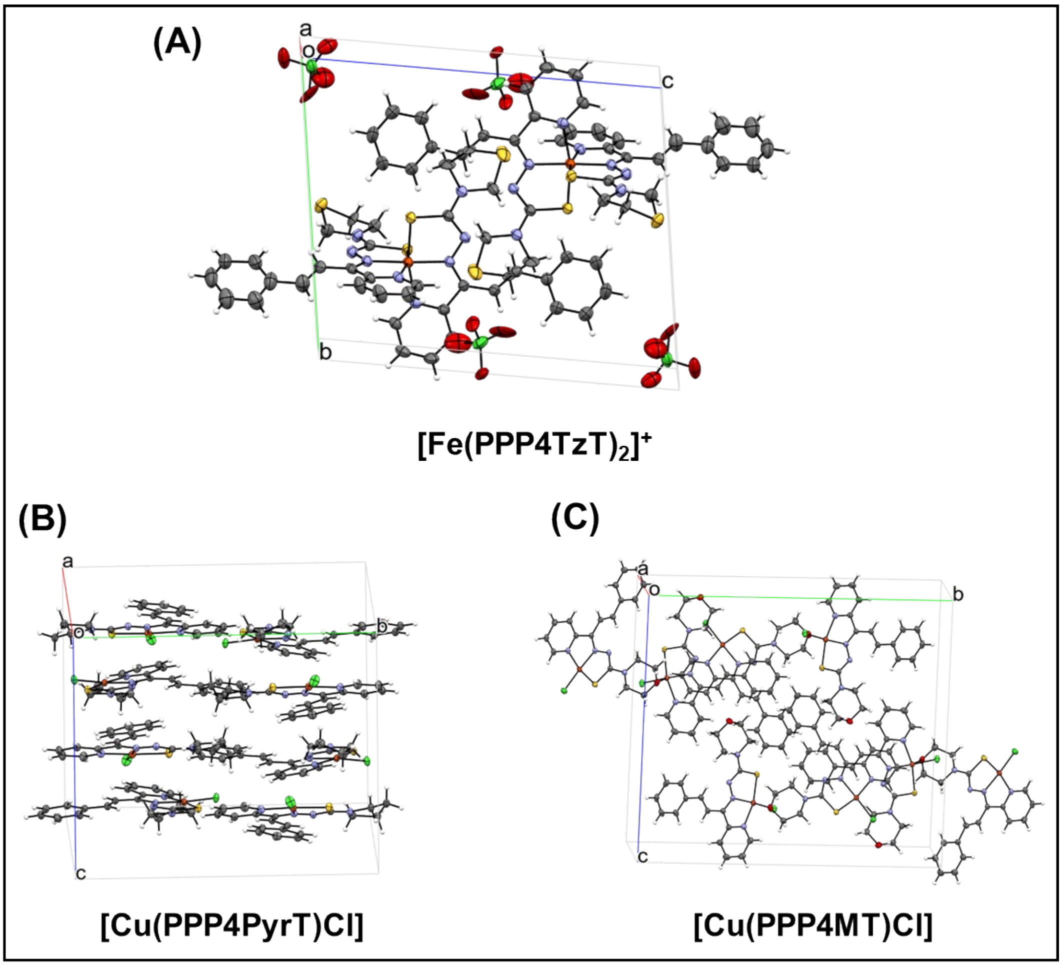

Figure S2. Unit cell packing diagrams for the complexes, namely: **(A)** [Fe(PPP4TzT)$_2$]$^+$; **(B)** [Cu(PPP4PyrT)Cl]; and **(C)** [Cu(PPP4MT)Cl]. H-atoms and disordered atoms are not shown for clarity.

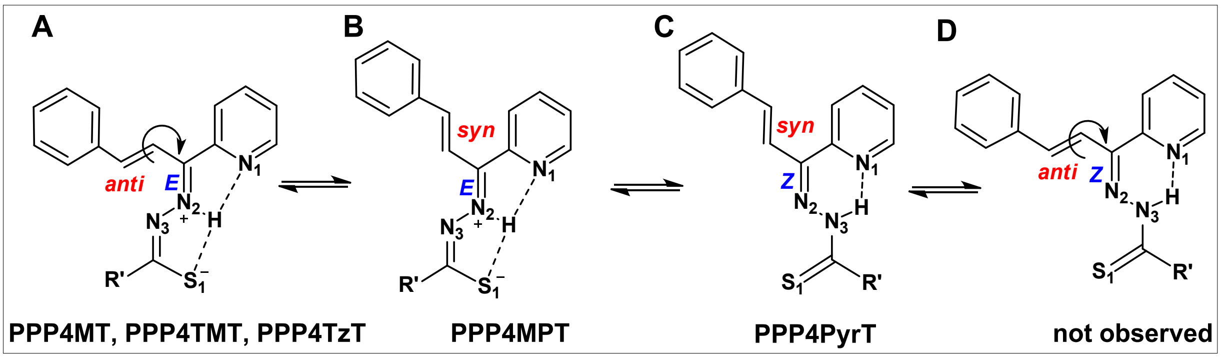

Figure S3. *E/Z* isomerism and *syn*/*anti*-conformations observed in the PPP4HAT analogues. **(A)** Structures of PPP4MT, PPP4TMT, and PPP4TzT showing the *E*-isomer with *anti*-conformation about the thiosemicarbazone bond. **(B)** Structure of PPP4MPT adopting the E-isomer with *syn*-conformation. **(C)** PPP4PyrT preferentially adopts the Z-isomer with a *syn*-conformation. **(D)** The *Z*-isomer with *anti*-conformation was not observed in any of the studied analogues.

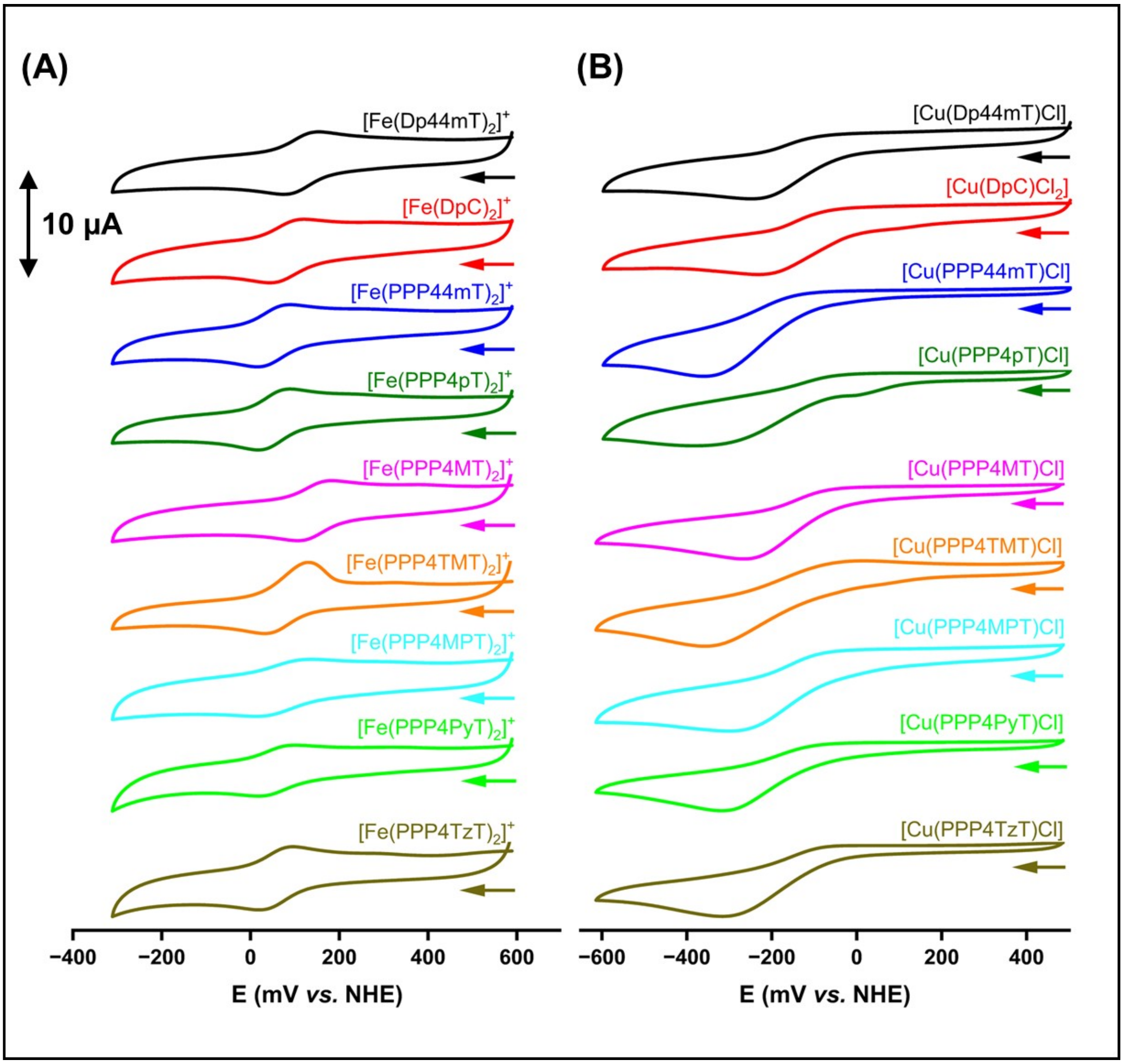

Figure S4 (A, B). Cyclic voltammograms of Fe(III) and Cu(II) complexes of Dp44mT, DpC, PPP44mT, and PPP4pT, shown relative to the newly synthesized Fe(III) and Cu(II) complexes of PPP4MT, PPP4TMT, PPP4MPT, PPP4PyrT, and PPP4TzT bearing terminally modified amines. All cyclic voltammetry was conducted using the complex (100 μM) in MeCN: H_2O (7:3 *v/v*) containing Bu_4NClO_4 (0.1 M) as supporting electrolyte. The sweep rate was 100 mV s^{-1}, and all scans were performed in the direction indicated by the arrows. Redox potentials are measured relative to the normal hydrogen electrode (NHE).

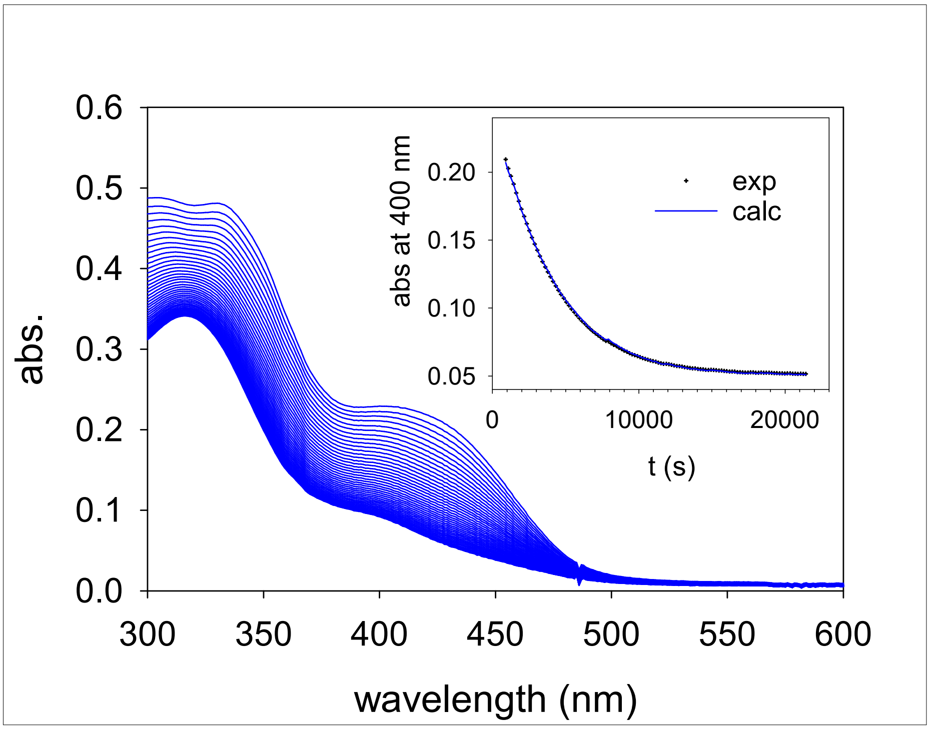

Figure S5. Time-dependent spectra (180 s intervals) of PPP4MPT (25 μM) in the presence of L-Cys (500 μM). The inset shows the fit to a first-order process (k_{obs} 2.74 ×10^{-4} s^{-1}). T = 37 °C, pH 7.4, [NaCl] = 0.14 M.

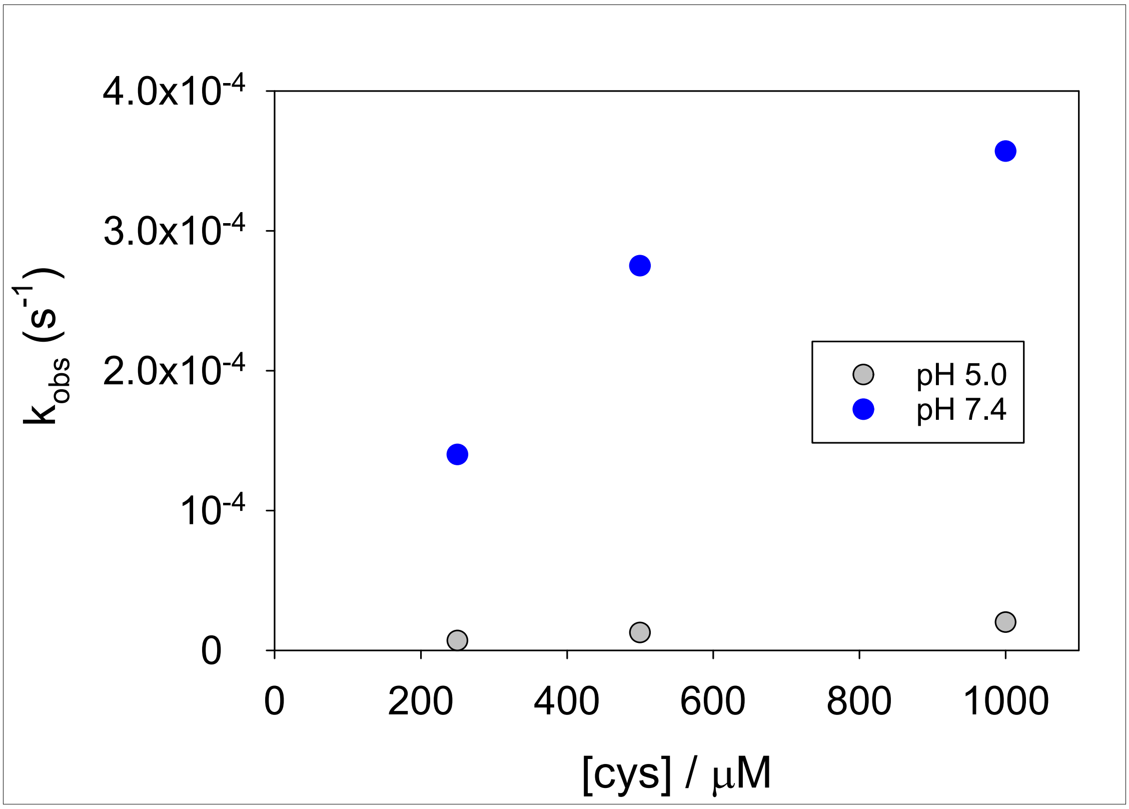

Figure S6. Observed first-order rate constants for the reaction between PPP4MPT (25 μM) and L-Cys at pH 7.4 and 5.0. Temperature 37 °C and [NaCl] = 0.14 M.

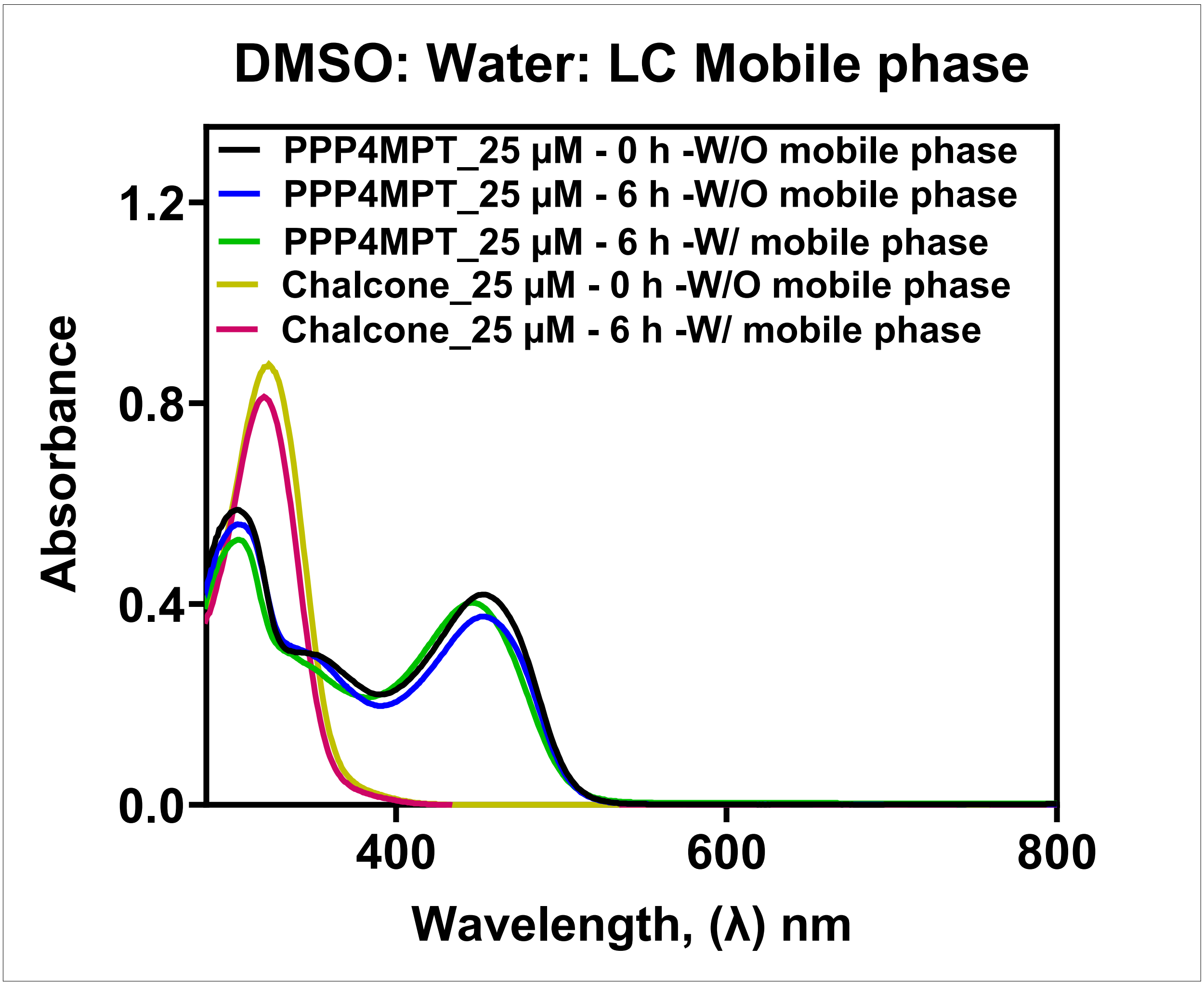

Figure S7. UV-Vis spectra of PPP4MPT (25 µM) and its respective chalcone compared at 0 h and after a 6 h/20 °C incubation in DMSO: water (7:3 *v/v*). This solution was then diluted 1:1 either with (W/) or without (W/O) the LC-MS mobile phase (90% MeOH, 9.9% water, and 0.1% formic acid) and incubated for a further 10 min/20 °C.

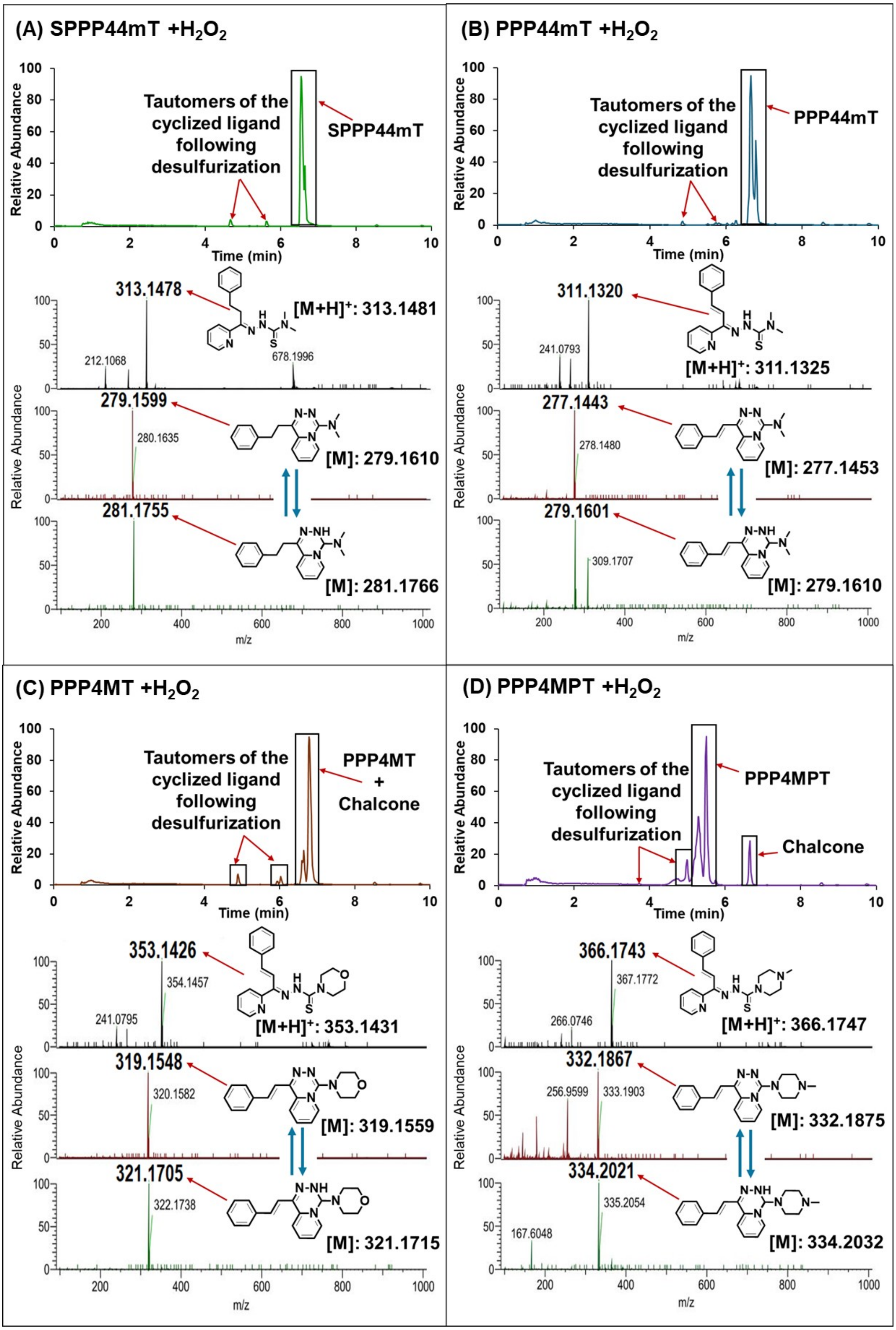

Figure S8. The desulfurization followed by intermolecular cyclization of: **(A)** SPPP44mT (500 µM), **(B)** PPP44mT (500 µM), **(C)** PPP4MT (500 µM), and **(D)** PPP4MPT (500 µM) following a 2 h/20 ºC incubation with H_2O_2 (10 mM) in the DMSO: H_2O (7:3 *v/v*) solvent system. For clarity, three chromatograms representing the ligand, and the two cyclized tautomers have been selected, and their corresponding MS spectra are shown to illustrate the reactions observed.

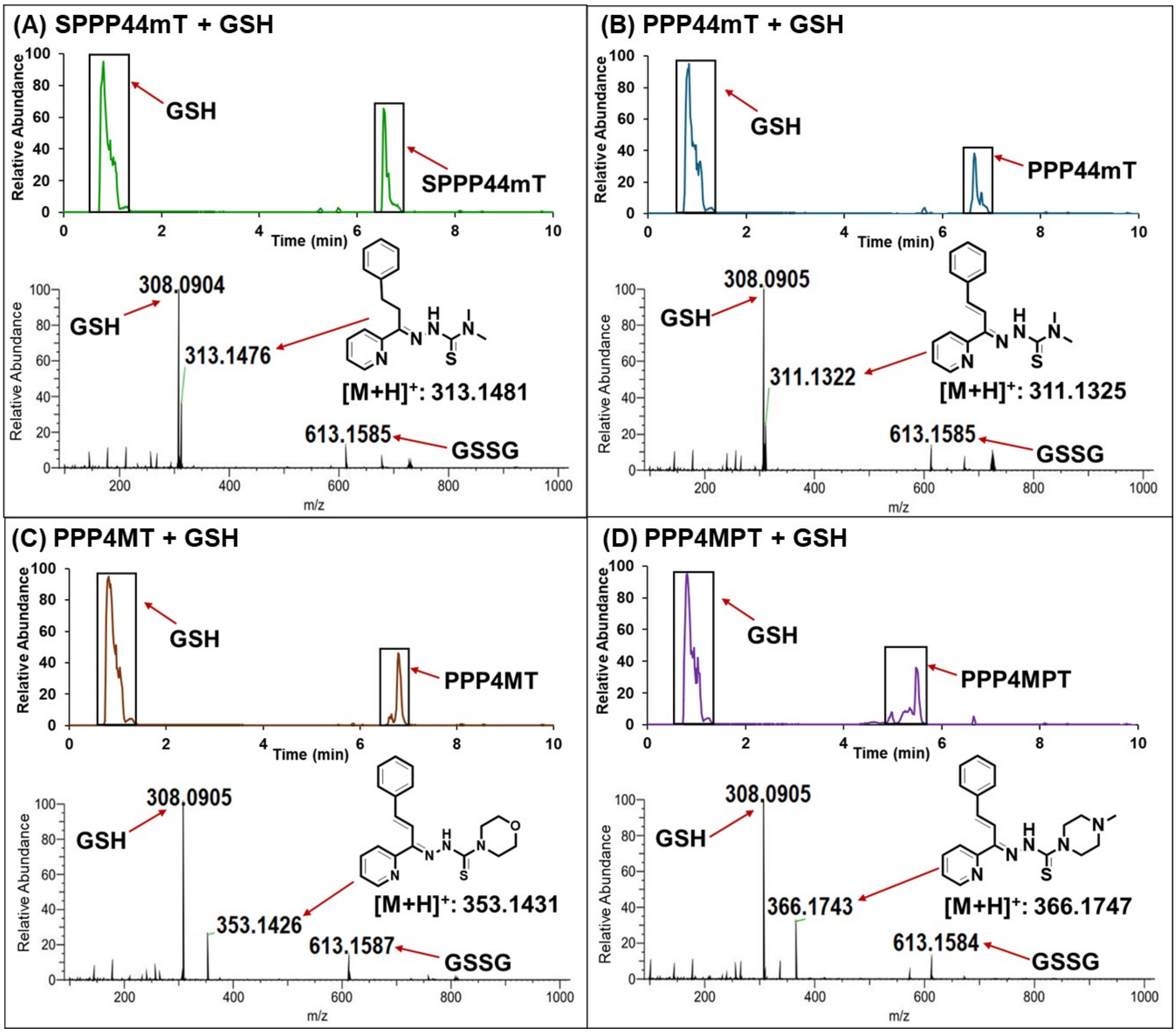

Figure S9. LC-MS analysis of: **(A)** SPPP44mT (500 μM), **(B)** PPP44mT (500 μM), **(C)** PPP4MT (500 μM), and **(D)** PPP4MPT (500 μM) after a 6 h/20 ºC incubation in the presence of GSH (5 mM) in DMSO/H_2O (7:3 *v/v*).

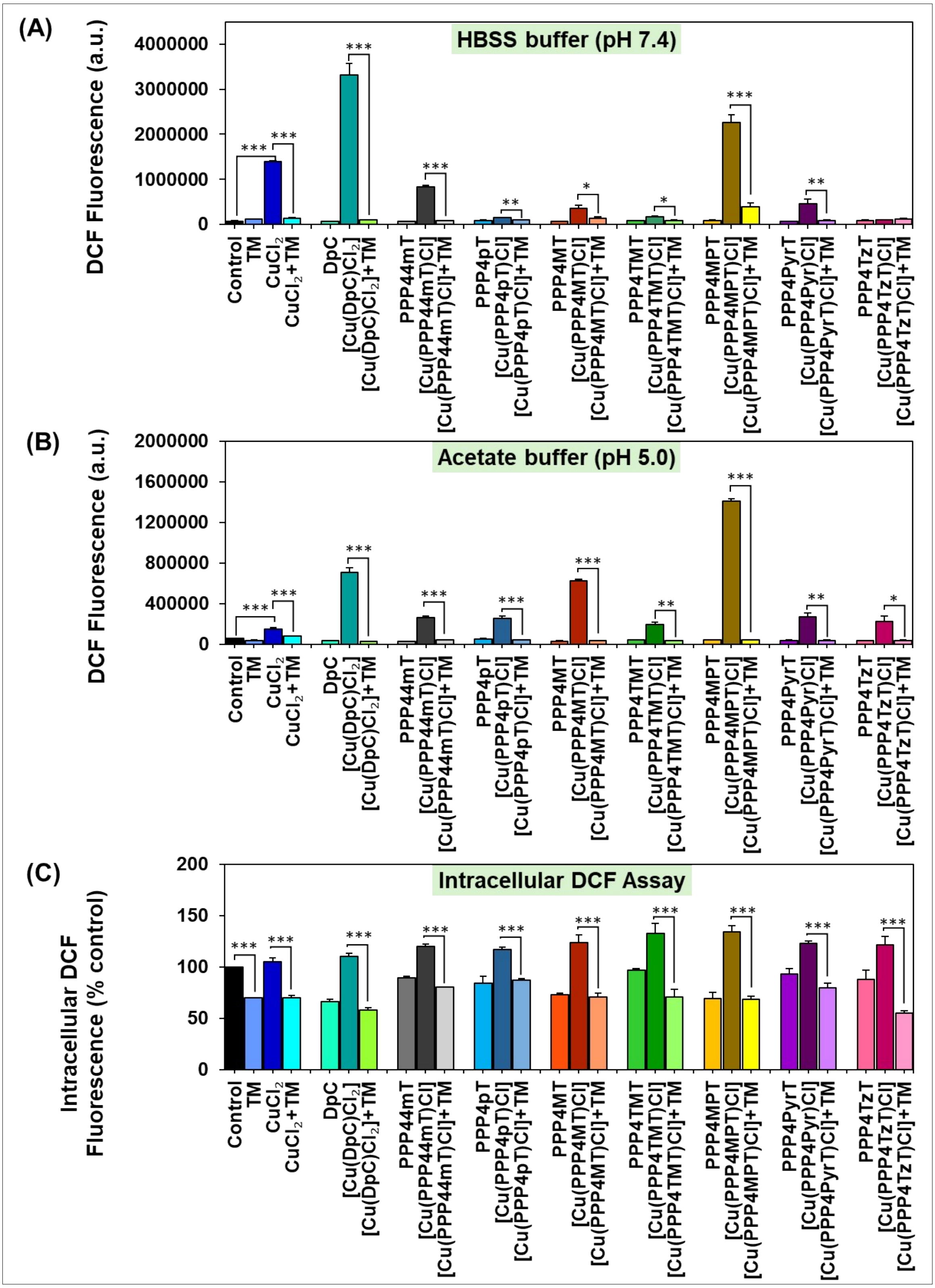

Figure S10. Extracellular redox activity of the 1:1 L:Cu(II) complexes of the PPP4HAT analogues compared with DpC, PPP44mT, and PPP4pT at: **(A)** pH 7.4 and **(B)** pH 5.0. Each agent (5 μM) was introduced into the respective buffers, followed by L-cysteine (100 μM). H_2DCF-DA (5 μM) was then added, and H_2O_2 (100 μM) was used to initiate the redox response. **(C)** Intracellular ROS generation measured by DCF-DA fluorescence following a 4 h treatment with the Cu(II) complexes of the PPP4HAT series (10 μM), in the absence or presence of tetrathiomolybdate (TM; 10 μM). After treatment, cells were incubated with H_2DCF-DA (5 μM) for 45 min/37 °C in the dark (covered with aluminum foil). Results are presented as mean ± SEM (n = 3).

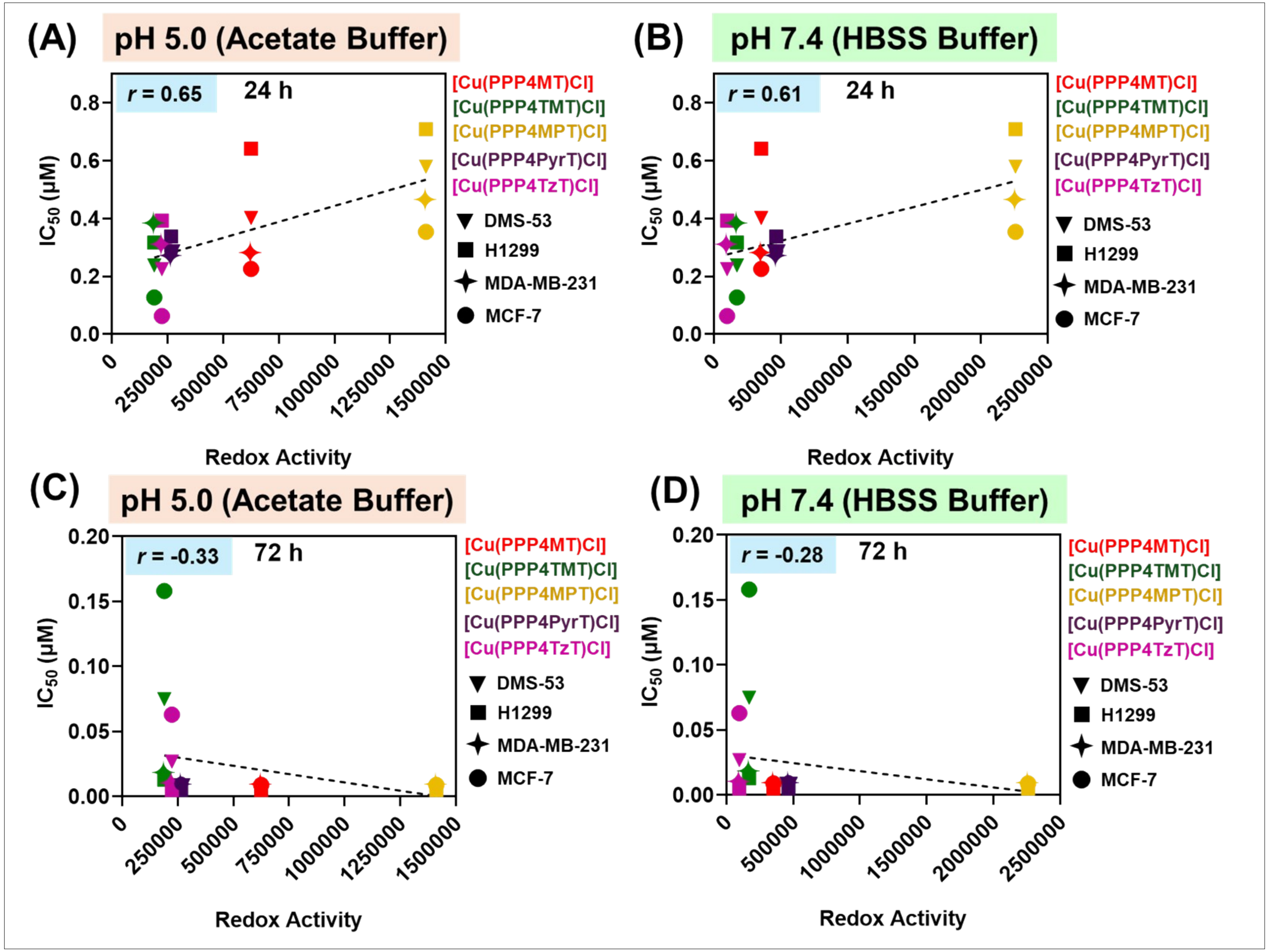

Figure S11. Plots of the DCF redox activity at pH 5.0 and pH 7.4 *versus* the anti-proliferative activity (IC_{50}) of the 1:1 Cu:L complexes of the PPP4HAT analogues against breast cancer cells (from **Table 1**) and lung cancer cells (**Table S3**) after an incubation of 24 h (**A, B**) or 72 h (**C, D**) at 37 °C. Results are means from 3 experiments.

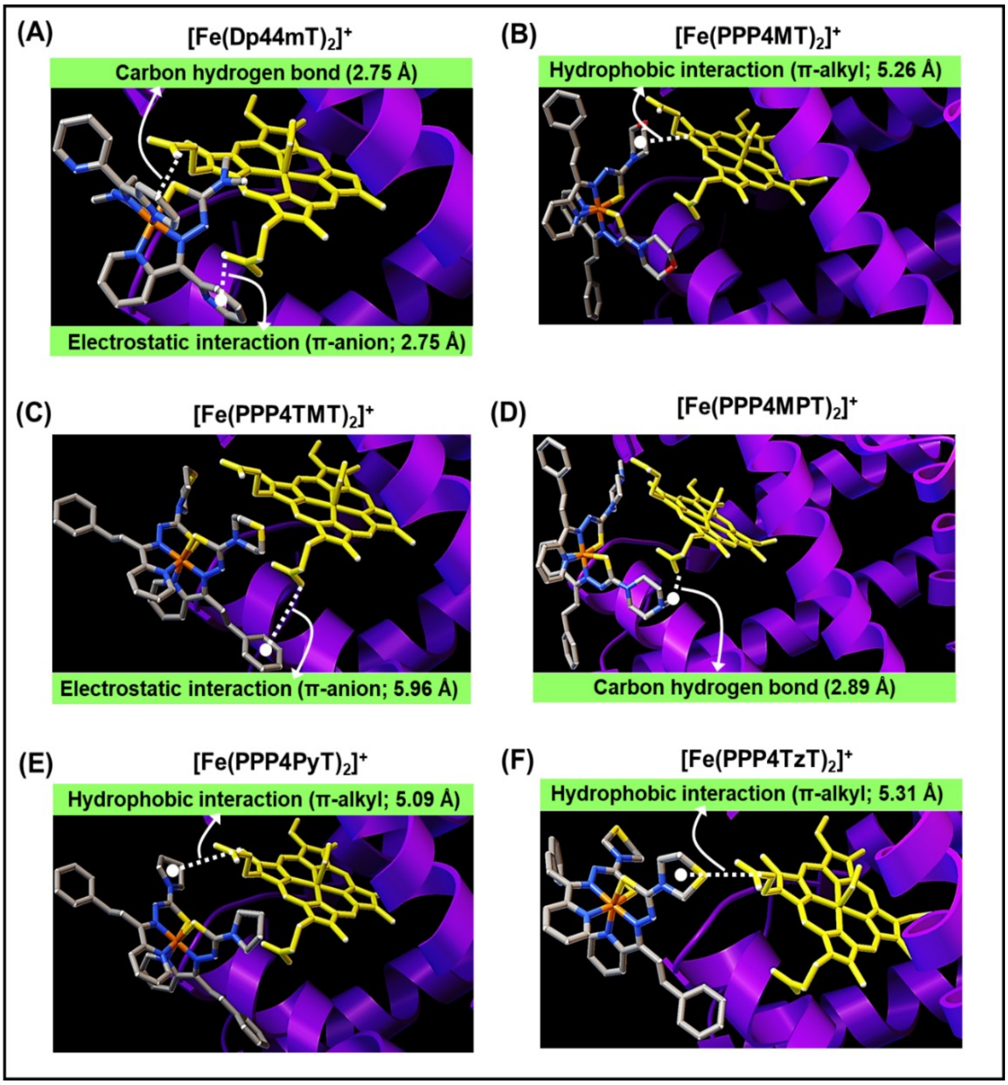

Figure S12. (A-F) Molecular docking illustrations of representative Fe(III) complexes with oxy-Mb showing key interaction types and distances. **(A)** $[Fe(Dp44mT)_2]^+$ engages in a π-anion interaction and a carbon-hydrogen bond (2.75 Å), enabling close heme association. **(B)** $[Fe(PPP4MT)_2]^+$ forms a π–alkyl hydrophobic interaction (5.26 Å) that limits heme approach. **(C)** $[Fe(PPP4TMT)_2]^+$ engages in a π-anion interaction (5.96 Å). **(D)** $[Fe(PPP4MPT)_2]^+$ exhibits a carbon-hydrogen bond (2.89 Å), consistent with strong docking proximity. **(E)** $[Fe(PPP4PyrT)_2]^+$ shows a π–alkyl hydrophobic interaction (5.09 Å). **(F)** $[Fe(PPP4TzT)_2]^+$ shows a π–alkyl hydrophobic interaction (5.31 Å), indicating partial steric hindrance. Docking simulations were visualized with the heme shown in yellow, the protein backbone in purple ribbons, and Fe(III) complexes in stick representation.

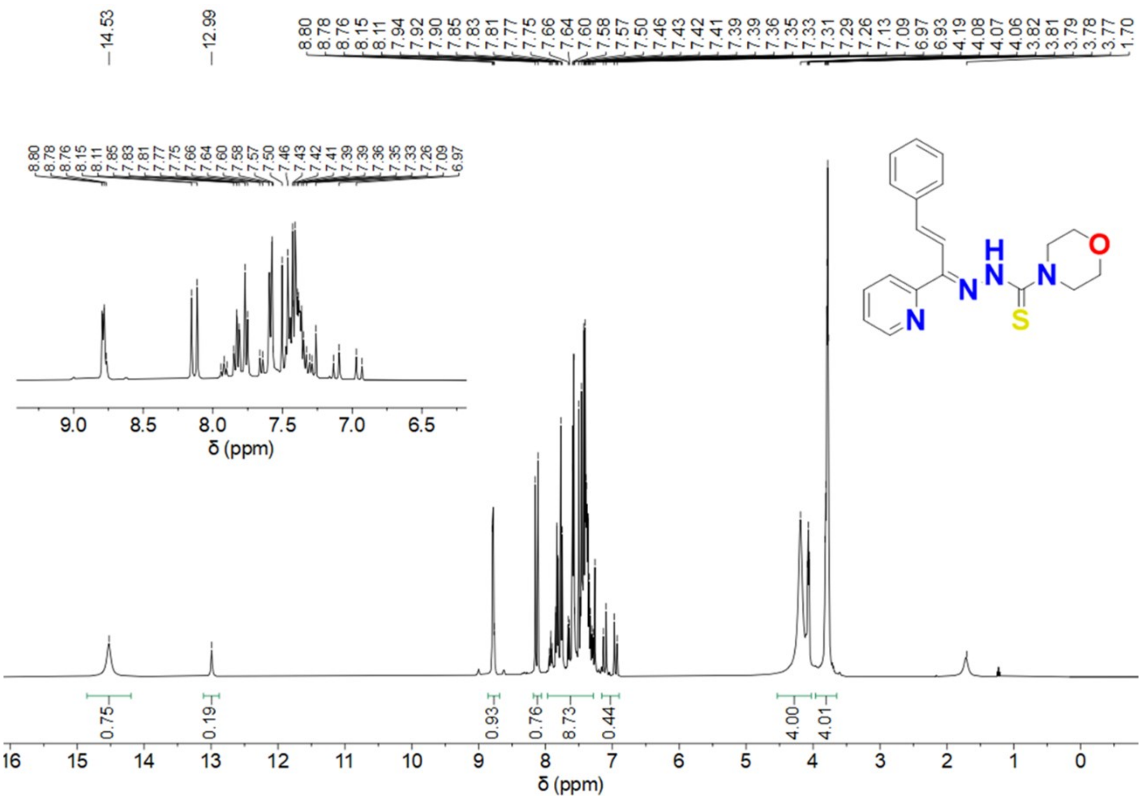

Figure S13. ^{1}H (400 MHz) NMR spectrum of PPP4MT in $CDCl_3$.

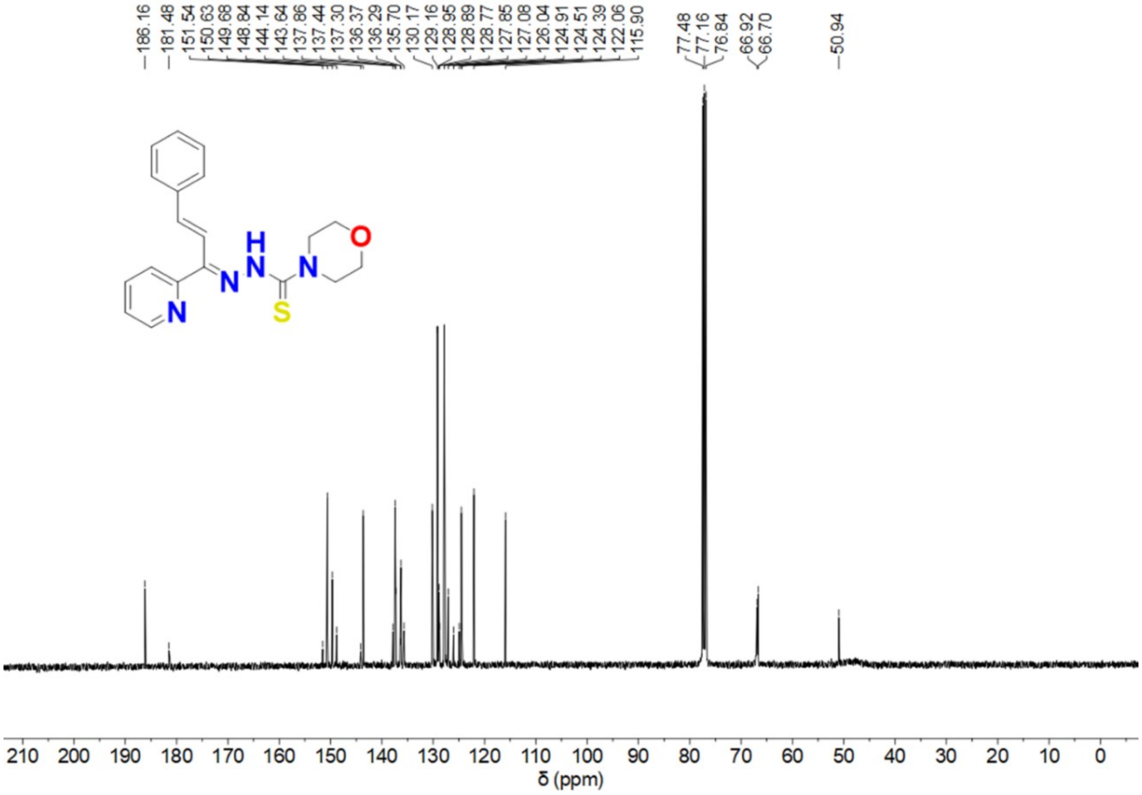

Figure S14. $^{13}C\{^1H\}$ (100 MHz) NMR spectrum of PPP4MT in $CDCl_3$

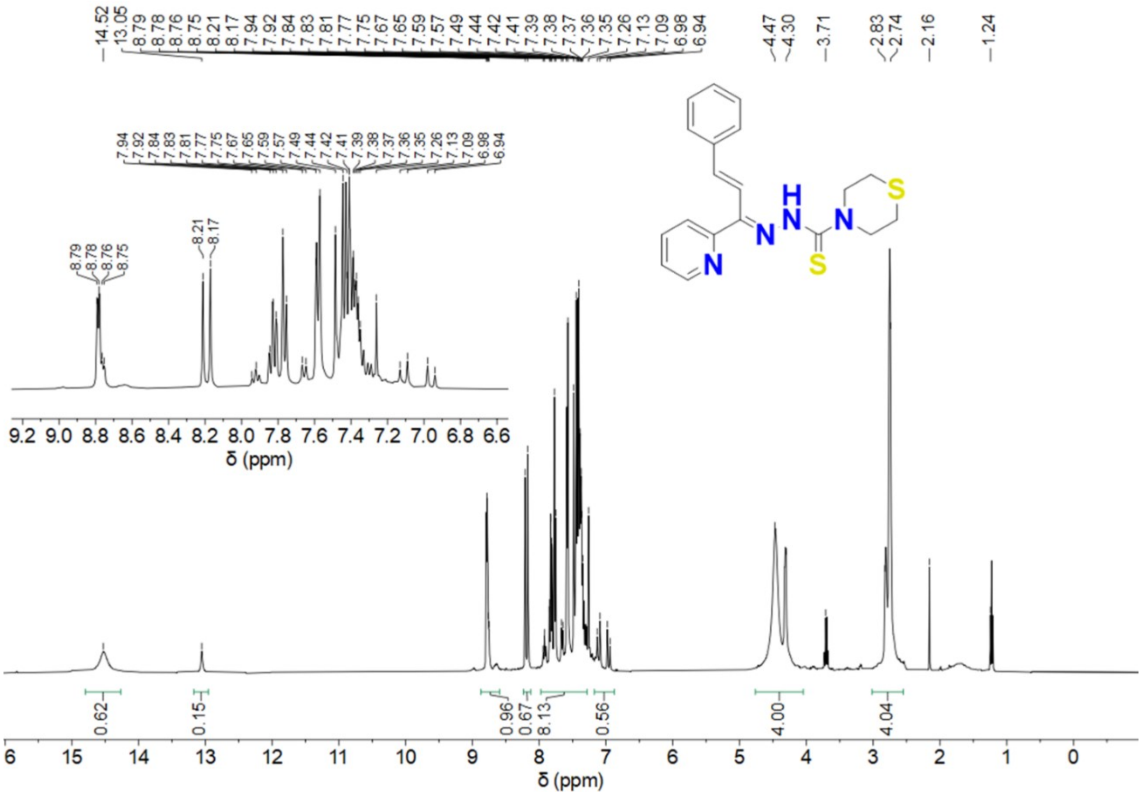

Figure S15. ^{1}H (400 MHz) NMR spectrum of PPP4TMT in $CDCl_3$.

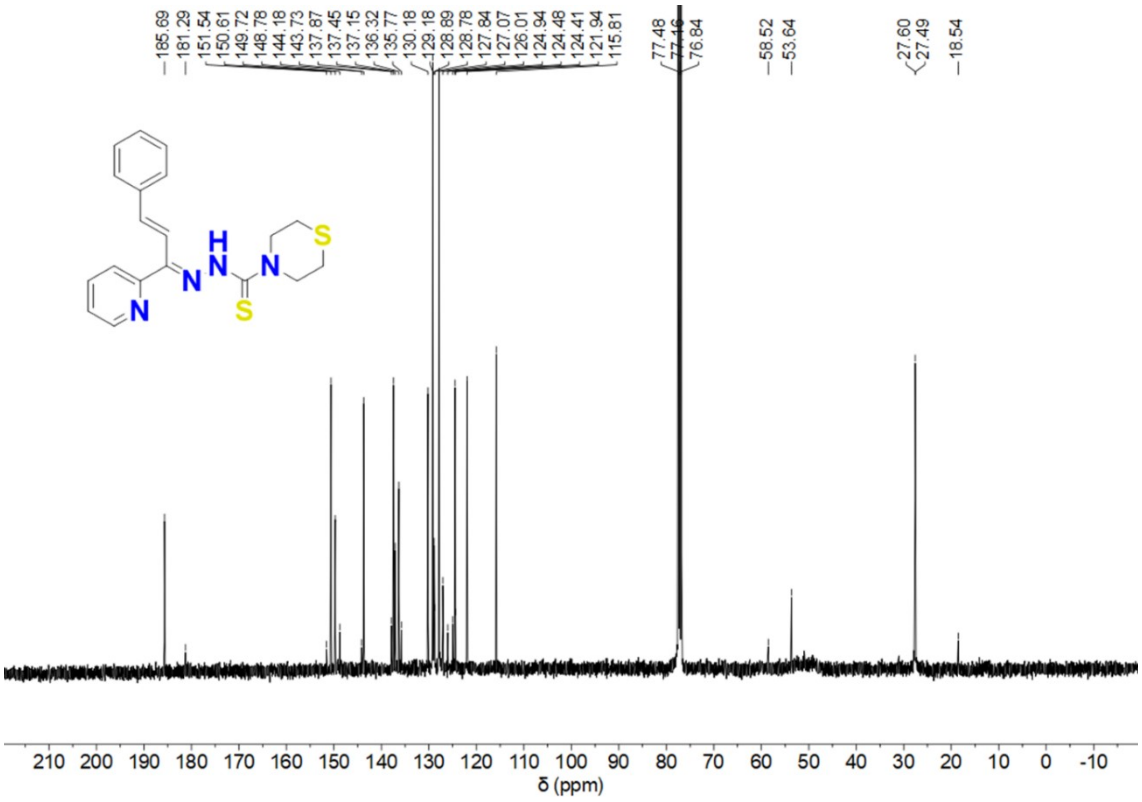

Figure S16. $^{13}C\{^{1}H\}$ (100 MHz) NMR spectrum of PPP4TMT in $CDCl_3$.

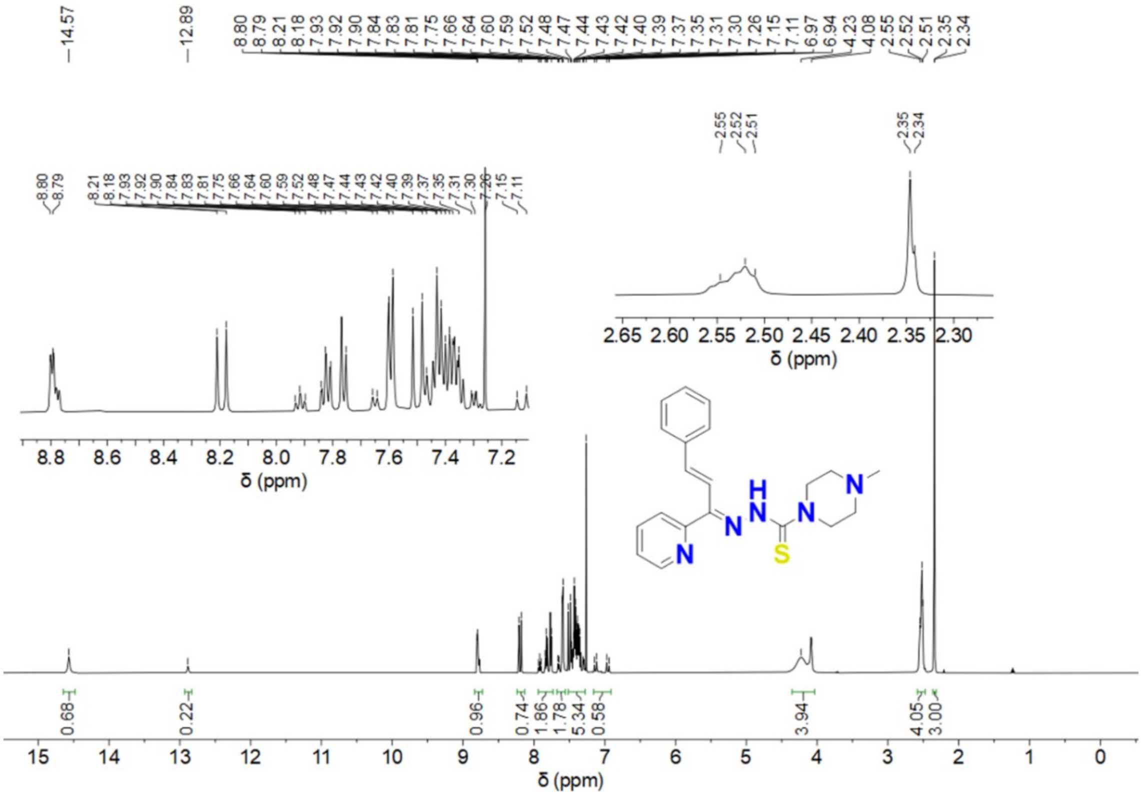

Figure S17. ^{1}H (400 MHz) NMR spectrum of PPP4MPT in $CDCl_3$.

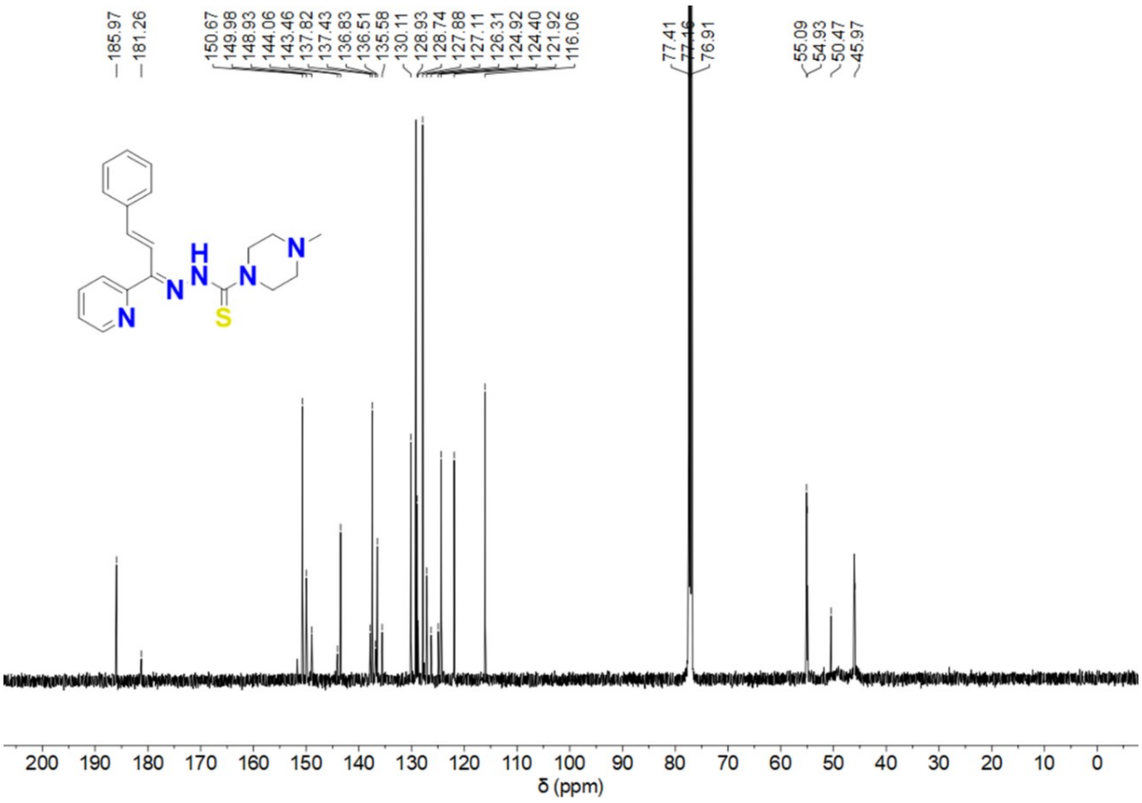

Figure S18. $^{13}C\{^{1}H\}$ (100 MHz) NMR spectrum of PPP4MPT in $CDCl_3$.

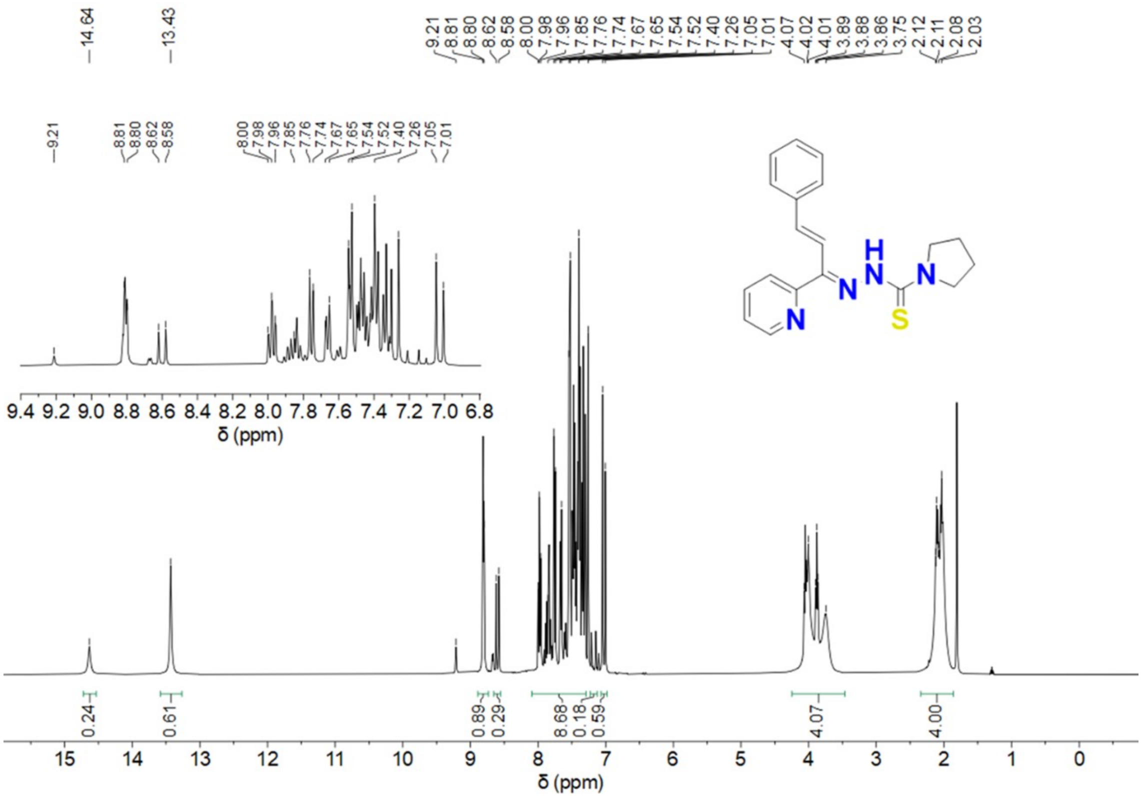

Figure S19. ^{1}H (400 MHz) NMR spectrum of PPP4PyrT in $CDCl_3$.

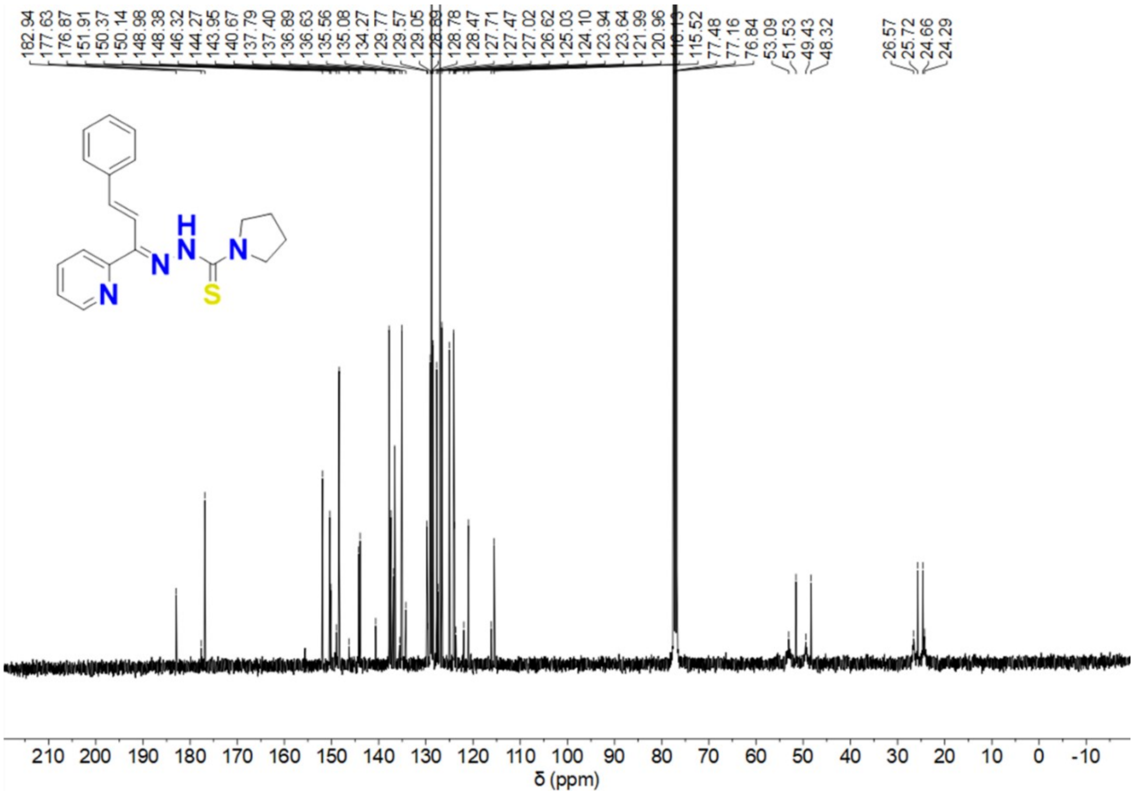

Figure S20. $^{13}C\{^{1}H\}$ (100 MHz) NMR spectrum of PPP4PyrT in $CDCl_3$.

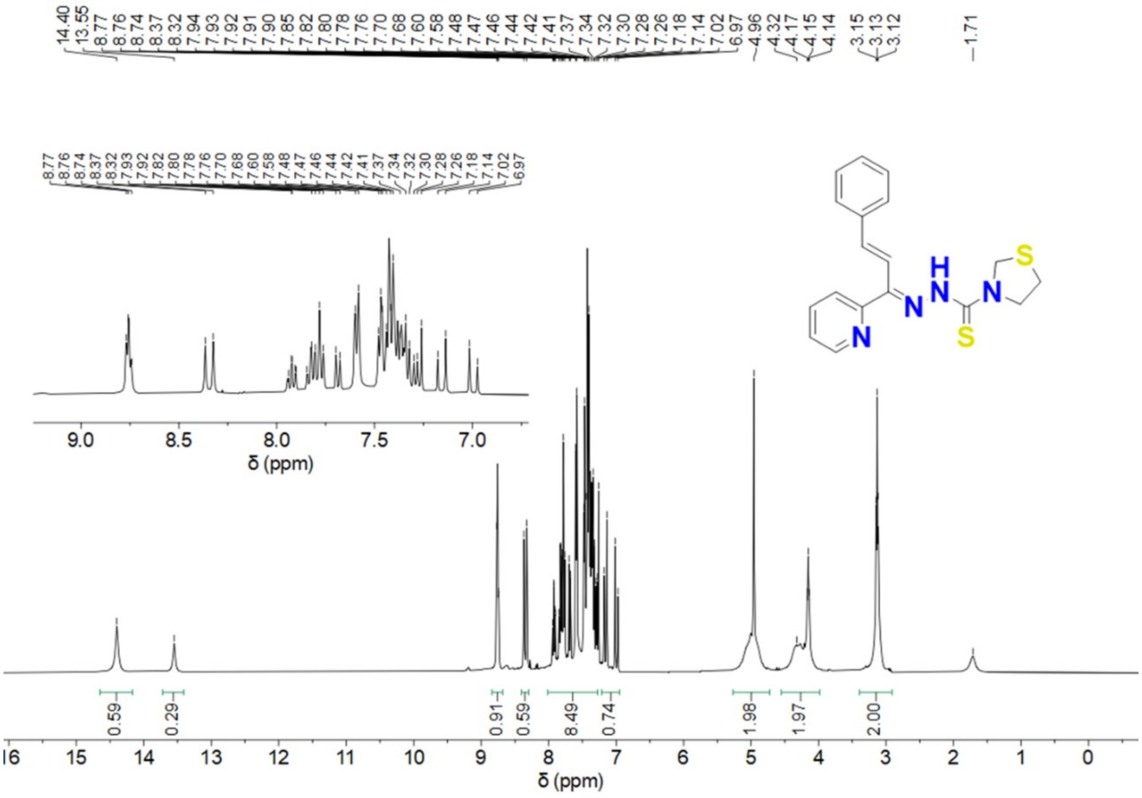

Figure S21. ^{1}H (400 MHz) NMR spectrum of PPP4TzT in $CDCl_3$.

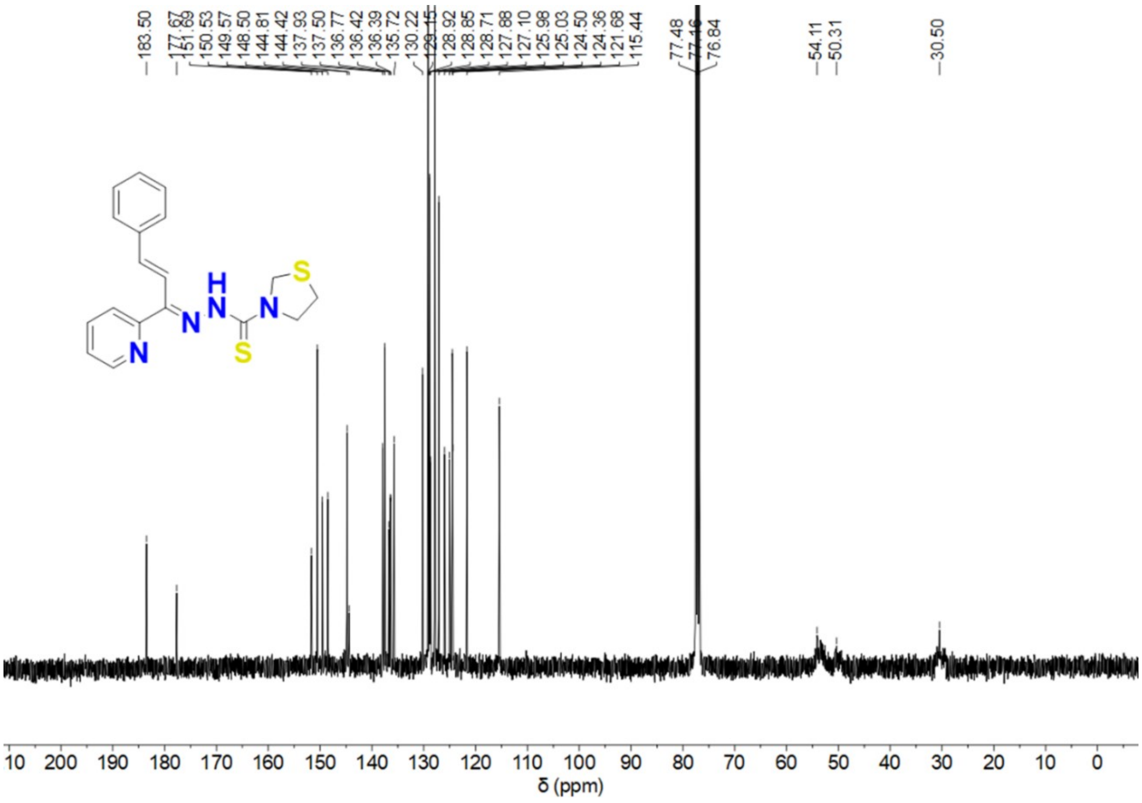

Figure S22. $^{13}C\{^{1}H\}$ (100 MHz) NMR spectrum of P PP4TzT in $CDCl_3$.

References

1. D. B. Lovejoy, D. M. Sharp, N. Seebacher, P. Obeidy, T. Prichard, C. Stefani, M. T. Basha, P. C. Sharpe, P. J. Jansson and D. S. Kalinowski, *J. Med. Chem.*, 2012, **55**, 7230-7244.

2. M. Dharmasivam, B. Kaya, T. Wijesinghe, M. Gholam Azad, M. A. Gonzalvez, M. Hussaini, J. Chekmarev, P. V. Bernhardt and D. R. Richardson, *J. Med. Chem.*, 2023, **66**, 1426-1453.

3. T. P. Wijesinghe, B. Kaya, M. A. Gonzalvez, J. R. Harmer, M. Gholam Azad, P. V. Bernhardt, M. Dharmasivam and D. R. Richardson, *J. Med. Chem.*, 2023, **66**, 15453-15476.

4. D. R. Richardson, P. C. Sharpe, D. B. Lovejoy, D. Senaratne, D. S. Kalinowski, M. Islam and P. V. Bernhardt, *J. Med. Chem.*, 2006, **49**, 6510-6521.

5. P. J. Jansson, P. C. Sharpe, P. V. Bernhardt and D. R. Richardson, *J. Med. Chem.*, 2010, **53**, 5759-5769.

6. A. E. Stacy, D. Palanimuthu, P. V. Bernhardt, D. S. Kalinowski, P. J. Jansson and D. R. Richardson, *J. Med. Chem.*, 2016, **59**, 8601-8620.

7. G. M. Sheldrick, *Acta Crystallogr. A*, 2008, **64**, 112-122.

8. G. M. Sheldrick, *Acta Crystallogr. C Struct. Chem.*, 2015, **71**, 3-8.

9. D. R. Richardson and E. Baker, *Biochim. Biophys. Acta*, 1990, **1053**, 1-12.

10. D. R. Richardson, E. H. Tran and P. Ponka, *Blood*, 1995, **86**, 4295-4306.

11. B. Kaya, M. Gholam Azad, M. Suleymanoglu, J. R. Harmer, T. P. Wijesinghe, V. Richardson, X. Zhao, P. V. Bernhardt, M. Dharmasivam and D. R. Richardson, *J. Med. Chem.*, 2024, **67**, 12155-12183.

12. M. Dharmasivam, S. Zhang, X. Zhao, V. Richardson, T. P. Wijesinghe, M. Suleymanoglu, M. Gholam Azad, P. V. Bernhardt, B. Kaya and D. R. Richardson, *J. Med. Chem.*, 2025, **68**, 9594-9622.

13. M. Maeder and P. King, *Jplus Consulting Pty Ltd, East Fremantle, WA, Australia*, 2009.

14. A. E. Stacy, D. Palanimuthu, P. V. Bernhardt, D. S. Kalinowski, P. J. Jansson and D. R. Richardson, *J. Med. Chem.*, 2016, **59**, 4965-4984.

15. J. A. Bailey, *J. Chem. Educ.*, 2011, **88**, 995-998.

16. G. M. Morris, R. Huey, W. Lindstrom, M. F. Sanner, R. K. Belew, D. S. Goodsell and A. J. Olson, *J. Comput. Chem.*, 2009, **30**, 2785-2791.

17. N. T. Nguyen, T. H. Nguyen, T. N. H. Pham, N. T. Huy, M. V. Bay, M. Q. Pham, P. C. Nam, V. V. Vu and S. T. Ngo, *J. Chem. Inf. Model.*, 2019, **60**, 204-211.

18. N. M. O'Boyle, M. Banck, C. A. James, C. Morley, T. Vandermeersch and G. R. Hutchison, *J. Cheminform.*, 2011, **3**, 33.
19. E. F. Pettersen, T. D. Goddard, C. C. Huang, G. S. Couch, D. M. Greenblatt, E. C. Meng and T. E. Ferrin, *J. Comput. Chem.*, 2004, **25**, 1605-1612.
20. M. A. Al-Sha'er, H. A. Basheer and M. O. Taha, *Mol. Divers.*, 2023, **27**, 443-462.
21. J. Gao and D. R. Richardson, *Blood*, 2001, **98**, 842-850.
22. M. Whitnall, J. Howard, P. Ponka and D. R. Richardson, *P. Natl. Acad. Sci. U.S.A.*, 2006, **103**, 14901-14906.
23. D. B. Lovejoy, D. M. Sharp, N. Seebacher, P. Obeidy, T. Prichard, C. Stefani, M. T. Basha, P. C. Sharpe, P. J. Jansson, D. S. Kalinowski, P. V. Bernhardt and D. R. Richardson, *J. Med. Chem.*, 2012, **55**, 7230-7244.
24. S. Schwan, D. Schroder, H. A. Wegner, J. Janek and D. Mollenhauer, *ChemSusChem*, 2020, **13**, 5480-5488.
25. D. C. Ashley and E. Jakubikova, *Inorg. Chem.*, 2018, **57**, 9907-9917.
26. S. K. Marvadi, V. S. Krishna, D. Sriram and S. Kantevari, *Eur. J. Med. Chem.*, 2019, **164**, 171-178.
27. R. Guo and R. W. Murray, *J. Am. Chem. Soc.*, 2005, **127**, 12140-12143.
28. N. Vo, N. L. Haworth, A. M. Bond and L. L. Martin, *Chemelectrochem*, 2018, **5**, 1173-1185.
29. K. Suzuki and K. Yamasaki, *J. Inorg. Nucl. Chem.*, 1962, **24**, 1093-1103.
30. F. Bernardi, I. G. Csizmadia, H. B. Schlegel and S. Wolfe, *Can. J. Chem.*, 1975, **53**, 1144-1153.
31. T. B. Chaston, R. N. Watts, J. Yuan and D. R. Richardson, *Clin. Cancer Res.*, 2004, **10**, 7365-7374.
32. D. B. Lovejoy, P. J. Jansson, U. T. Brunk, J. Wong, P. Ponka and D. R. Richardson, *Cancer Res.*, 2011, **71**, 5871-5880.
33. M. Dharmasivam, B. Kaya, T. P. Wijesinghe, V. Richardson, J. R. Harmer, M. A. Gonzalvez, W. Lewis, M. G. Azad, P. V. Bernhardt and D. R. Richardson, *Chem. Sci.*, 2024, **15**, 974-990.
34. Z. Wang, Y. H. Zhang, C. Guo, H. L. Gao, M. L. Zhong, T. T. Huang, N. N. Liu, R. F. Guo, T. Lan, W. Zhang, Z. Y. Wang and P. Zhao, *Front. Aging Neurosci.*, 2018, **10**, 9.
35. H. M. Alvarez, Y. Xue, C. D. Robinson, M. A. Canalizo-Hernandez, R. G. Marvin, R. A. Kelly, A. Mondragon, J. E. Penner-Hahn and T. V. O'Halloran, *Science*, 2010, **327**, 331-334.
36. E. E. Battin and J. L. Brumaghim, *Cell Biochem. Biophys.*, 2009, **55**, 1-23.
37. C. H. Foyer and G. Noctor, *Plant Cell Environ.*, 2016, **39**, 951-964.

38. M. G. Azad, T. Russell, X. Gu, X. Zhao, V. Richardson, T. P. Wijesinghe, G. Babu, X. Guo, B. Kaya, M. Dharmasivam, Z. Deng and D. R. Richardson, *J. Biol. Chem.*, 2025, **301**, 110230.
39. S. V. Menezes, S. Sahni, Z. Kovacevic and D. R. Richardson, *J. Biol. Chem.*, 2017, **292**, 12772-12782.
40. F. Shehadeh-Tout, H. H. Milioli, S. Roslan, P. J. Jansson, M. Dharmasivam, D. Graham, R. Anderson, T. Wijesinghe, M. G. Azad, D. Richardson and Z. Kovacevic, *Pharmacol. Res.*, 2023, **193**, 106806.
41. C. A. Lipinski, *Drug Discov. Today Technol.*, 2004, **1**, 337-341.
42. C. A. Lipinski, F. Lombardo, B. W. Dominy and P. J. Feeney, *Adv. Drug Deliv. Rev.*, 2012, **64**, 4-17.
43. D. F. Veber, S. R. Johnson, H.-Y. Cheng, B. R. Smith, K. W. Ward and K. D. Kopple, *J. Med. Chem.*, 2002, **45**, 2615-2623.
